# Intermolecular hydrogen‐bonded associates of BODIPYs drive controllable aggregates and enhanced NIR phototherapeutic performance

**DOI:** 10.1002/smo2.70041

**Published:** 2026-03-11

**Authors:** Siying Gou, Zhe Xun, Peijun Yang, Xin Li, Dongxiang Zhang, Jianjun Du, Shuo Li, Xin‐Dong Jiang, Gaowu Qin

**Affiliations:** ^1^ Institute for Strategic Materials and Components Liaoning & Shenyang Key Laboratory of Functional Dye and Pigment Shenyang University of Chemical Technology Shenyang China; ^2^ Department of Biochemistry and Molecular Biology School of Life Sciences China Medical University Shenyang Liaoning China; ^3^ State Key Laboratory of Fine Chemicals Dalian University of Technology Dalian China

**Keywords:** associate, BODIPY, controllable aggregation, intermolecular hydrogen‐bond, NIR

## Abstract

This study herein presents the rational design and synthesis of a series of *meso*‐CF_3_‐substituted BODIPY dyes to achieve controllable molecular aggregation and enhanced near‐infrared (NIR) phototherapeutic performance. By constructing a D‐A‐D architecture and introducing furan/thiophene groups, the resulting BODIPYs (**OBB**, **OHB**, **SBB** and **SHB**) form distinct intermolecular aggregates. Single‐crystal X‐ray diffraction analyses reveal that specific hydrogen‐bonding interactions (C‐H⋯F), with precise distances ranging from 2.336 Å to 2.542 Å, induce self‐assembly to form hydrogen‐bonded‐mediated *J* aggregates. This controlled aggregation induces significant bathochromic shifts, extending absorption into the NIR‐I and NIR‐II regions (up to 937 nm in film), and generates broad absorption bands beneficial for laser matching. Among the self‐assembled nanoparticles (NPs), **OBB** NPs demonstrate superior properties, including a high photothermal conversion efficiency of 49.7%, a large Stokes shift (87 nm) in water, and type‐I reactive oxygen species generation. In vitro studies against ovarian cancer cells confirm that **OBB** NPs exhibit low dark toxicity but potent photo‐triggered cytotoxicity, effectively inducing cell apoptosis and necrosis through an ROS‐mediated mechanism. This work underscores the critical role of intermolecular hydrogen bonding in manipulating aggregation‐induced optical properties and provides a fundamental strategy for developing highly effective organic phototherapeutic agents.

## Graphical abstract

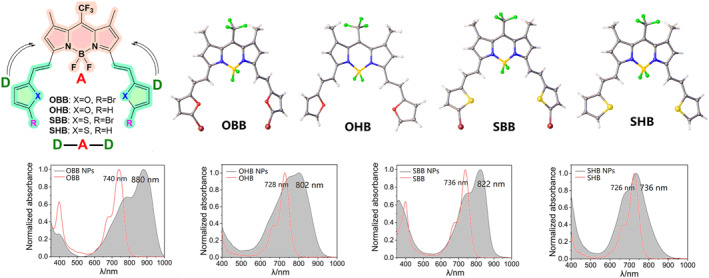

## 1 INTRODUCTION

Long‐wavelength dyes exhibit unique advantages in deeper tissue penetration, higher spatial resolution, reduced autofluorescence background with an enhanced signal‐to‐noise ratio, as well as the ability to utilize higher imaging power levels while inducing lower phototoxicity.[^1^, ^2^, ^3^] Therefore, the development of long‐wavelength dyes is of great importance in biological diagnosis and treatment. The classic strategies to induce spectral bathochromic shifts of dyes are as follows: extending the conjugated system,[^4^, ^5^, ^6^] constructing a push‐pull electron system (D‐A),[^7^, ^8^, ^9^] introducing heteroatoms,[^10^, ^11^] and forming *J*‐aggregation or intermolecular hydrogen‐bonded associates (IHBAs).^[12]^

Non‐covalent interactions of *J*‐aggregates or IHBAs serve as the cornerstone for constructing complexity and functionality. Especially, hydrogen bonding stands out as an intermolecular stronger force with unique strength, directionality, and selectivity.[^13^, ^14^] It is not only fundamental to the existence and maintenance of biological systems but also a powerful tool in materials science for designing novel functional materials.[^15^, ^16^] Although the importance of hydrogen bonding is widely recognized, precisely predicting and controlling the associative behavior of specific molecular systems in complex environments (e.g., self‐assembled nanomaterials) remains a major challenge in the field.^[17]^ This study focuses on IHBAs, which refer to extended two‐dimensional aggregates formed through the spontaneous and highly directional self‐assembly of multiple identical molecules via hydrogen bonds.[^18^, ^19^] The properties of these materials, including their optical characteristics, are directly governed by the mode and strength of their intermolecular associations.

Moreover, in D‐A‐D architectures, slip‐stacked packing motifs are favored, where the central acceptor moiety in one molecule interacts with the donor moieties in neighboring molecules.[^20^, ^21^, ^22^] The particular arrangement of these interactions can also result in conventional *J*‐aggregation, CT‐coupled *J*‐aggregation, or hydrogen‐bonded intermolecular associates.[^23^, ^24^, ^25^] Fluorescent dyes, including cyanine, perylenediimide, squaranine and BODIPY, exhibit *J*‐aggregation behavior in the aggregate.[^26^, ^27^] However, the narrow absorption peak of *J*‐aggregation meets with an unsatisfactory matching between the laser wavelength and absorption peak, making the selection of appropriate lasers for phototherapy difficult.[^28^, ^29^] Herein, intermolecular hydrogen‐bonded couplings in *J* aggregate produce spectral broadening, which not only improves the matching with laser wavelengths but also provides a critical pathway toward polychromatic light absorption.

Controlling the stacking direction and angle of molecules is a crucial and challenging task. Thus, this work further systematically investigates the formation mechanisms, structural features, and functional properties of hydrogen‐bonded intermolecular associates of BODIPYs. By integrating spectroscopic analysis, crystal structure determination, and theoretical computational simulations, we sought to elucidate the driving forces and principles underlying hydrogen bond association in model molecular systems. This study is expected not only to deepen the understanding of hydrogen bonding as a fundamental intermolecular force but also to provide a solid theoretical foundation and design principles for developing novel smart materials based on hydrogen bonds, such as self‐assembled nanoparticles. Moreover, the IHBAs of BODIPYs were demonstrated to be applicable to photodynamic‐photothermal therapy for ovarian cancer cells in the NIR region.

## 2 RESULTS AND DISCUSSION

**FIGURE 1:**
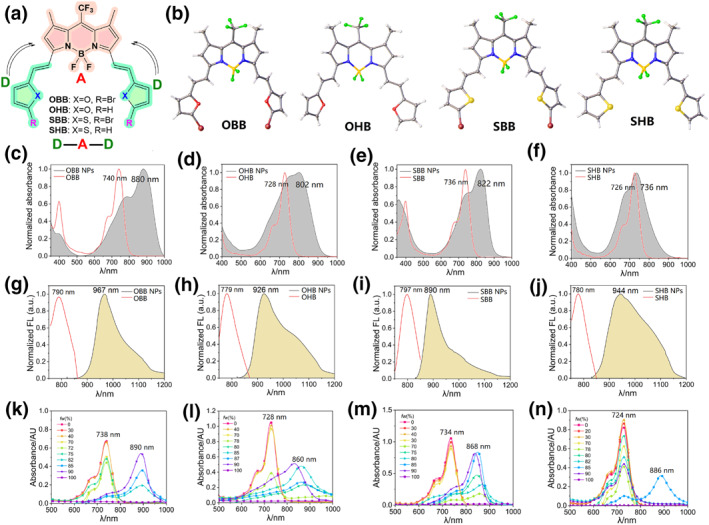
(a) Molecular structures of BODIPYs **OBB**, **OHB**, **SBB** and **SHB**. (b) Oak Ridge thermal ellipsoid plot (ORTEP) views of **OBB** (CCDC 2490362), **OHB** (CCDC 2490363), **SBB** (CCDC 2490364) and **SHB** (CCDC 2490365) (displacement ellipsoids at the 30% probability level). (c–f) Absorption spectra of **OBB**, **OHB**, **SBB** and **SHB** in CH_2_Cl_2_ and absorption spectra of **OBB** NPs, **OHB** NPs, **SBB** NPs and **SHB** NPs in water. (g–j) Emission spectra of **OBB**, **OHB**, **SBB** and **SHB** in CH_2_Cl_2_ and emission spectra of **OBB** NPs, **OHB** NPs, **SBB** NPs and **SHB** NPs in water. Absorption spectra of (k) **OBB**, (l) **OHB**, (m) **SBB** and (n) **SHB** in different proportions of THF‐H_2_O for the aggregation effect.

In this study, a two‐step synthetic strategy was employed to prepare the target dyes BODIPYs (Figure 1a). Firstly, a precursor bearing a trifluoromethyl (−CF_3_) rotator moiety at the *meso*‐site was prepared, using 2,4‐dimethylpyrrole as the starting material.[^30^, ^31^] Subsequently, furfural derivatives were selected as condensation agents, and BODIPYs with the D‐A‐D system were obtained via the Knoevenagel condensation (Figure 1a and Scheme S1).^[32]^ The chemical structures of all dyes were characterized by NMR spectroscopy and high‐resolution mass spectrometry in Supporting Information. Furthermore, the structures of the furan/thiophene‐substituted BODIPYs were unequivocally confirmed by X‐ray analysis (Figure 1b). Through the side view of the single crystal, it can be seen that the BF_2_ group of **OBB** deviates significantly from the parent nucleus of BODIPY and the extremely severe distortion compared to the other three dyes (Figure S1). So, we are curious about what force causes it and how it affects the spectral performance.

**FIGURE 2:**
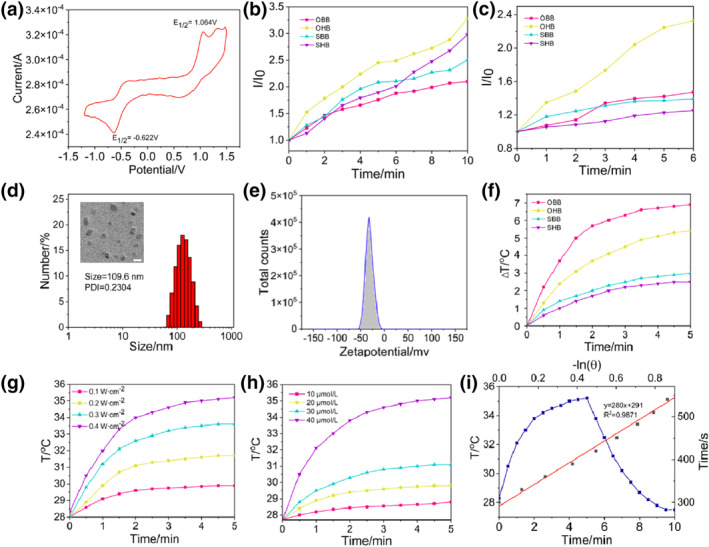
(a) Cyclic voltammograms of 4.0 mM **OBB** measured in CH_2_Cl_2_ containing 0.1 M TBAPF_6_ as the supporting electrolyte at room temperature. Glassy carbon electrode as a working electrode, and the scan rate at 100 mV s^−1^. Monitoring of reactive oxygen species (ROS) generation from **OB‐BDP**, **OH‐BDP**, **SB‐BDP** and **SH‐BDP** (5 μM) in DMSO under continuous 808 nm laser (0.3 W/cm^2^) irradiation for 10 min using (b) DCFH (10 μM) or (c) DHR123 (10 μM) as an indicator. (d) Dynamic light scattering (DLS) and transmission electron microscopy (TEM) of self‐assembled **OBB** NPs, Scale bar: 100 nm. (e) Zeta potential of 40 μM **OBB** NPs. (f) Temperature difference performance of **OB‐BDP**, **OH‐BDP**, **SB‐BDP** and **SH‐BDP** NPs (40 μM) under 0.4 W cm^−2^ light irradiation. (g) Photothermal conversion of **OBB** NPs (40 μM) under 808 nm laser irradiation with different power density (0.1–0.4 W cm^−2^). (h) Temperature changes of **OBB** NPs at different concentrations (10–40 μM) under 808 nm laser irradiation (0.4 W/cm^2^). (i) Temperature response curves of **OBB** NPs in aqueous solutions under irradiation and natural cooling, and linear fitting of –Ln*θ* and time.

First, we gained insight into their spectral performance. In the organic solvent CH_2_Cl_2_ (Figures S2–S5, Table S1), the maxima absorption of the single molecule of four dyes are located in the first near‐infrared (NIR‐I) range of 726–740 nm, with **OBB** showing the largest absorption value and **SHB** showing the smallest one, mainly due to the electron‐donating group (Figure 1c–f). Next, we examined the absorption spectra of self‐assembled nanoparticles (see ESI) and the film state to investigate their aggregation behavior. DSPE‐PEG_2000_ and BODIPYs were self‐assembled into micellar nanoparticles (NPs) (Seeing the following Figure 2d).[^33^, ^34^, ^35^] Dynamic light scattering analysis of the resulting **OBB** NPs indicated a hydrodynamic diameter in the range of 60–200 nm (Figure 2d), with an average size of approximately 109.6 nm and a polydispersity index (PDI) of 0.23.[^36^, ^37^, ^38^] The morphology of the nanoparticles was further examined using transmission electron microscopy,[^39^, ^40^, ^41^] which confirmed their well‐defined nanostructural shape (Figure 2d). Additionally, zeta potential measurements showed a surface charge of −32.7 mV for **OBB** NPs (Figure 2e), a value within the range typically associated with good colloidal stability.^[42]^ This highly negative zeta potential suggests strong electrostatic repulsion between individual nanoparticles, effectively preventing aggregation and promoting dispersion stability in the colloidal system.^[43]^ The maximum absorption wavelength of **OBB** NPs in water was observed at 880 nm, representing a red shift of 140 nm compared to that (λ_abs_ = 740 nm) in CH_2_Cl_2_ (Figure 1c–f). Similarly, **OHB** NPs and **SBB** NPs exhibited red shifts of 74 nm (λ_abs_ = 802 nm) and 86 nm (λ_abs_ = 822 nm), respectively (Figure 1c–f). In contrast, **SHB** NPs showed only a minor redshift of 10 nm, with absorption maxima at 736 nm in water compared to 728 nm in CH_2_Cl_2_ (Figure 1c–f). A comparable red shift was also evident in the absorption spectra of the film state; for instance, **OBB** displayed a maximum absorption at 937 nm (Figure S6). Based on the theoretical absorption spectra of the monomer and dimer of dye **OBB**, we found that the dimer exhibited a new characteristic peak shape. This indicates that the aggregate by intermolecular hydrogen‐bonding interactions caused a spectral redshift (Figure S7, Table S2).

**FIGURE 3:**
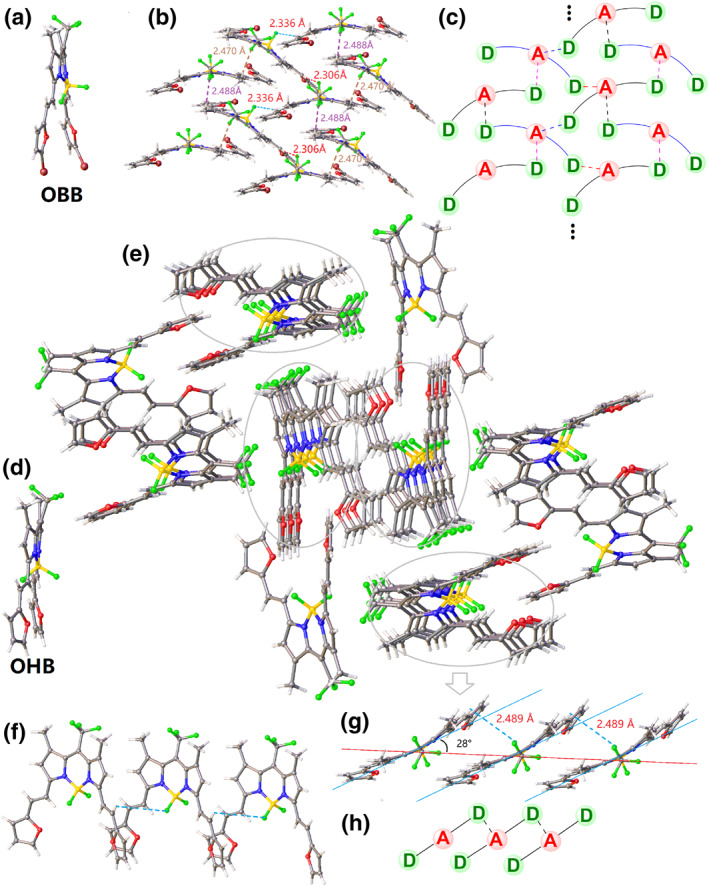
X‐ray single‐crystal structures of **OBB** and **OHB**. (a) Side view of the crystal packing of **OBB**. (b) Perspective view of the crystal packing of **OBB**. (c) Fish bone diagram based on Figure 3b (d) Side view of the crystal packing of **OHB**. (e) Perspective view of the crystal packing of **OHB**. (f–g) Illustration of the 1D domino‐like arrangement of **OHB**. (h) Fish bone diagram based on Figure 3g.

With their single crystal structures successfully obtained, we have gained a comprehensive understanding of their aggregation morphology in the crystalline state (Figure 3, Figures S8 and S9). This insight allows us to propose a plausible explanation for the observed spectroscopic redshift. In the crystal packing of **OBB** (Figure 3a), we identified three types of strong hydrogen bonds between adjacent molecules, with bond distances measuring 2.336 Å (C–H⋯F(BF_2_)), 2.470 Å (C–H⋯F(CF_3_)), and 2.488 Å (C–H⋯F(CF_3_)), respectively (Figure 3b). These interactions indicate the formation of IHBAs (Figure 3c), which contribute to the redshift behavior.^[44]^ For **OHB** (Figure 3d), top‐view analysis of the crystal packing shows that every three molecules assemble into a unit adopting a *J*‐aggregation configuration (Figure 3e–h). The presence of a short C–H⋯F(BF_2_) contact distance of 2.489 Å indicates strong intermolecular hydrogen bonding (Figure 3g), which plays a key role in enforcing this ordered packing motif and modulating the electronic interactions between adjacent molecules.^[45]^ This packing arrangement correlates well with the broadened and redshifted absorption features observed experimentally. Similarly, **SBB** also exhibits the *J*‐aggregate behavior, though with a slightly longer hydrogen bond distance (C–H⋯F(BF_2_): 2.542 Å) (Figure S8). In contrast, the crystal packing of **SHB** showed no significant intermolecular interactions (Figure S9), consistent with the single‐molecule behavior. Overall, these structural observations confirm that the D–A–D strategy effectively promotes diverse molecular aggregation modes and modulates intermolecular interactions.

Next, we characterized their fluorescence spectra (Figure 1g–j). While the four dyes exhibited a relatively narrow emission range (λ_em_ = 779–797 nm) in organic solvents, their nanoparticle forms showed the emission maxima across a broader range (λ_em_ = 890–967 nm). The fluorescence quantum yield of the *J* aggregates was found to be lower than that of the monomer, due to aggregation‐caused quenching (Table S3). Notably, the brominated **OBB** NPs displayed a Stokes shift of 87 nm in water, significantly larger than that (50 nm) observed in organic solvents. We also found that the non‐brominated **OHB** NPs exhibited an even larger Stokes shift of 124 nm in aqueous solution. It is worth noting that the Stokes shift of **SHB** NPs reached 208 nm in water.

To further gain insight into the aggregation behavior of **OBB**, **OHB**, **SBB** and **SHB**, their absorption properties were studied in THF–H_2_O mixed solvents (Figure 1k–n).^[46]^ In pure THF, **OBB** showed a sharp absorption band at around 738 nm. As the water fraction (f_w_) increased, the absorbance gradually decreased and underwent a red shift, reaching 890 nm at *f*_w_ = 90% (Figure 1k). Similarly, the other three dyes exhibited absorption maxima in the range of 860–880 nm at *f*_w_ = 85% (Figure 1l–n). These spectral shifts are attributed to restricted molecular motion and enhanced intermolecular interactions within the aggregated structures.

To unravel the optical properties of **OBB**, **OHB**, **SBB** and **SHB**, theoretical simulations were performed based on density functional theory (DFT) and time‐dependent density functional theory methods.[^47^, ^48^, ^49^] The primary absorption and emission peaks of these four molecules both correspond to the S_0_‐S_1_ transition, which is composed of the transition between the highest occupied molecular orbital and the lowest unoccupied molecular orbital, and the MO plots are presented in Figures S10 and 11. The experimental and theoretical maxima absorptions λ_DCM_ of **OBB**, **OHB**, **SBB** and **SHB** single molecules in dichloromethane (DCM) show good agreement, which confirms the reliability of our results, as shown in Table 1. Meanwhile, all the dyes possess high molar extinction coefficients, which provide a foundation for efficiently absorbing photons. Notably, the experimental maxima absorptions (λ_NP_) of **OBB**, **OHB**, **SBB** and **SHB** NPs exhibit significant bathochromic shifts (220, 169, 160 and 97 nm) compared to the theoretical maxima absorptions (λ_NAN_) of their monomeric counterparts in solvent‐free conditions (Table 1). These pronounced redshifts demonstrate that the molecular aggregation (via intermolecular hydrogen bonding interactions) substantially alters the electronic transitions in these systems, likely due to enhanced intermolecular charge delocalization.

**TABLE 1:** Experimental and theoretical optical parameters of **OBB**, **OHB**, **SBB** and **SHB**, where ΔE_g_ is the energy gap between the LUMO and HOMO, and S_1‐DCM_ and S_1‐NAN_ are the energy of excited state S_1_ of **OBB**, **OHB**, **SBB** and **SHB** single molecules in dichloromethane (DCM) and solvent‐free conditions, respectively. λ_DCM_ and λ_NAN_ are the maxima absorption of **OBB**, **OHB**, **SBB** and **SHB** single molecules in DCM and solvent‐free conditions, respectively. ε (M^−1^ cm^−1^) denotes the molar extinction coefficient determined from the UV–vis absorption spectra. λ_NP_ is the maximum absorption of **OBB**, **OHB**, **SBB** and **SHB** nanoparticles in water.

Dye	ΔE_g_/eV	S_1‐DCM_/eV	λ_DCM_/nm		ε/(M^−1^cm^−1^)	S_1‐NAN_/eV	λ_NAN_/nm	λ_NP_/nm
			Theoretical	Experimental				
**OBB**	1.88	1.69	733	740	145,000	1.88	660	880
**OHB**	1.85	1.73	719	728	156,000	1.96	633	802
**SBB**	1.85	1.70	730	736	148,000	1.87	662	822
**SHB**	1.87	1.73	717	726	158,000	1.94	639	736

*Note*: The light gray and light orange zones denote the theoretical data calculated by Orca and experimental data, respectively.

Abbreviations: HOMO, highest occupied molecular orbital; LUMO, lowest unoccupied molecular orbital.

Moreover, the electrochemical behavior of **OBB** was investigated using cyclic voltammetry. The measurements revealed a reversible reduction potential at −0.622 V and an oxidation potential at 1.064 V (Figure 2a). According to the relationship *Δ*E_HL_ ≈ ΔE × *e*,^[50]^ the HOMO–LUMO energy gap of **OBB** is close to the difference between the redox potentials (1.686 V), supporting the validity of the MO calculations. Both redox processes were found to be electrochemically reversible.

Next, the overall reactive oxygen species (ROS) generation capabilities of the four dyes were explored by using 2′,7′‐dichlorodihydrofluorescein (DCFH).^[51]^ Based on the fluorescence changes of DCFH (Figure 2b), a certain amount of ROS could be produced upon photoinduction. To further identify the type of ROS, the efficiency of BODIPYs in producing singlet oxygen (^1^O_2_) was investigated based on the absorbance attenuation of 1,3‐diphenylisobenzofuran (DPBF) as a singlet oxygen indicator.^[52]^ As shown in Figures S12–S15, the absorbance of DPBF at 416 nm only showed a little decrease for **OHB** and **SHB** with increasing irradiation time and almost no singlet oxygen was produced. Next, the generation of type‐I ROS was assessed using dihydrorhodamine 123 (DHR123).^[53]^ As depicted in Figure 2c, all four dyes induced changes in the DHR123 fluorescence intensity over a 6‐min irradiation period, indicating efficient type‐I ROS generation. This is attributed to the twisting‐induced ISC.

Next, the photothermal performances of four nanoparticles, **OBB** NPs, **OHB** NPs, **SBB** NPs and **SHB** NPs (Figure 2d,e, Figure S16), were evaluated by monitoring the temperature as a function of irradiation time under varying laser power densities and nanoparticle concentrations. Among the four NPs evaluated, **OBB** NPs exhibited the most robust photothermal performance across all tested conditions (Figure 2f), including temperature elevation under varying power densities, cycling stability, and concentration‐dependent effects (Figure S17). As shown in Figure 2g, the temperature of the solutions of **OBB** NPs exhibited a positive correlation with the increasing laser power density (ranging from 0.1 to 0.4 W cm^−2^) over a 5‐min irradiation period. Upon the irradiation of 0.1 W·cm^−2^, only a modest temperature rise was observed. In contrast, under 0.4 W cm^−2^ irradiation, the temperature increased markedly, reaching approximately 35.1°C. These results demonstrate that higher power densities significantly enhance the photothermal conversion efficiency (PCE) of **OBB** NPs. As shown in Figure 2h, the influence of **OBB** NPs concentration (10–40 μmol/L) on the photothermal response was investigated. Higher concentrations led to more pronounced temperature increases, with the 40 μmol/L solution reaching a significantly elevated temperature compared to the 10 μmol/L solution within 5 min. Nevertheless, an optimal concentration range may need to be identified for practical biomedical applications. Figure S17b shows the cyclic photothermal performance of **OBB** NPs under periodic laser irradiation. The temperature rose rapidly during laser‐on phases and returned to baseline during the off periods, demonstrating high reversibility over five consecutive cycles. This reproducible thermal response confirms the outstanding photothermal stability of **OBB** NPs, a crucial characteristic for repeated use in photothermal therapy. The PCE was quantified for all four NPs. According to the relevant time constants obtained from the cooling time and temperature (Figure 2i),^[54]^ **OBB** NPs achieved the highest PCE value of 49.7%, whereas the other three nanoparticles all showed PCE values below 48% (Figures S17 and S18). These results collectively indicate that **OBB** NPs possess superior photothermal performance under laser irradiation compared to the other NPs studied. Meanwhile, **OBB** NPs exhibit good stability in different buffer systems (cell culture medium and PBS) (Figures S19–S21). Therefore, we chose **OBB** NPs for the subsequent biological study.

**FIGURE 4:**
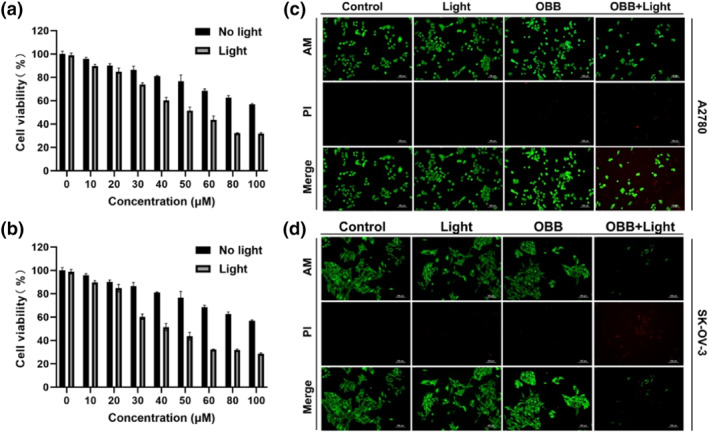
In vitro cytotoxicity of **OBB** NPs. (a) Cell viability was analyzed by CCK‐8 assay in A2780 cells after different treatment; (b) Cell viability was analyzed by CCK‐8 assay in SK‐OV‐3 cells; (c) Live‐dead status of A2780 cells evaluated by Calcein‐AM/PI co‐staining after photothermal treatment (0.3 W/cm^2^, 5 min) with 50 μM **OBB** NPs; (d) Live‐dead status of SK‐OV‐3 cells with 40 μM **OBB** NPs. Scale bar: 100 μm. For clarity, **OBB** NPs are abbreviated as **OBB** in Figure 4.

The potential cytotoxicity of **OBB** NPs was assessed by CCK8 assay against human ovarian cancer cells with or without laser irradiation.^[55]^ As shown in Figure 4a,b, without the laser radiation, **OBB** NPs showed remarkable biocompatibility with A2780 and SK‐OV‐3 cells, as the survival rate of A2780 and SK‐OV‐3 cells was above 60% even at 100 μM. However, **OBB** NPs with light irradiation for 5 min exhibited much lower cell viability within the same dose range, indicating that **OBB** NPs possess low dark cytotoxicity and high phototoxicity. The inhibitory effects of **OBB** NPs on A2780 and SK‐OV‐3 cells were concentration‐dependent, with the inhibitory rate reaching 50% at a concentration of 50 μM for A2780 cells and 40 μM for SK‐OV‐3 cells, which were selected as the conditions for subsequent experiments to further investigate the phototherapeutic performance of **OBB** NPs.

Then, we performed live‐dead cell staining to visualize the cell cytotoxicity of **OBB** NPs under laser irradiation by using calcine AM (green) and propidium iodide (red) dyes.^[56]^ As anticipated, significant cell death was observed in the A2780 cell line following 5‐min laser irradiation (0.3 W/cm^2^) in the presence of 50 μM **OBB** NPs (Figure 4c). Correspondingly, the SK‐OV‐3 cell line also exhibited a significantly increased proportion of dead cells after exposure to the same laser conditions with 40 μM **OBB** NPs (Figure 4d). These phenomena were evidenced by increased red fluorescence due to the loss of cell membrane integrity. In contrast, cells exposed to light alone or **OBB** NPs alone exhibited predominant green fluorescence, similar to the control group, indicating minimal impact on cell viability (Figure 4c,d). The results indicated that **OBB** NPs with laser irradiation have the potential to induce human ovarian cancer cell death.

**FIGURE 5:**
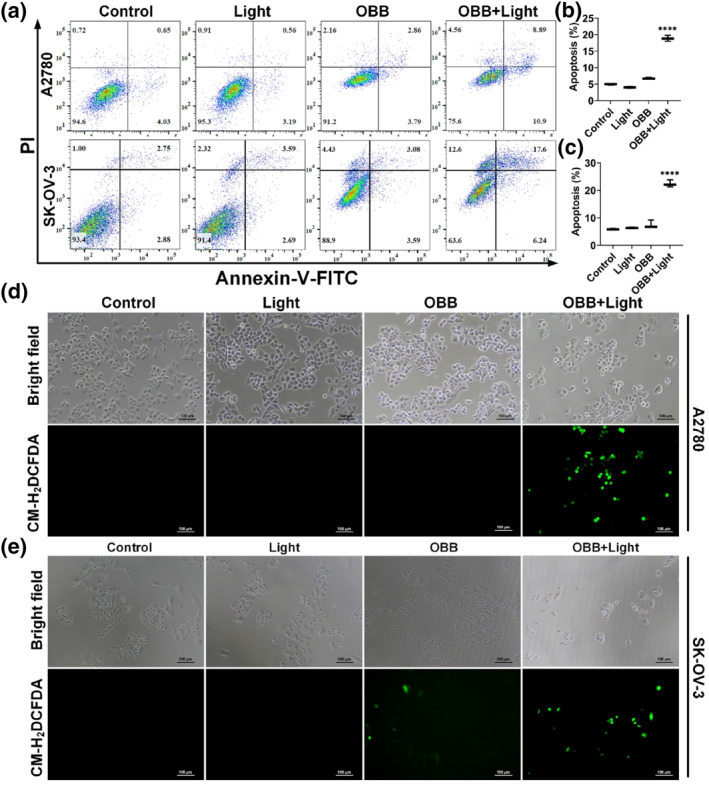
Effects of **OBB** NPs on apoptosis and reactive oxygen species (ROS) Levels. (a) Flow cytometry analysis for cell apoptosis of A2780 cells and SK‐OV‐3 cells after treatment of **OBB** NPs (50 μM **OBB** NPs for A2780 cells and 40 μM **OBB** NPs for SK‐OV‐3 cells) alone, light alone, or the combination with light irradiation (0.3 W/cm^2^, 5 min) with **OBB** NPs; (b) Quantitative analysis of apoptotic A2780 cells by flow cytometry (total of the early apoptotic and late apoptotic cells percentages); (c) Quantitative analysis of apoptotic SK‐OV‐3 cells by flow cytometry (total of the early apoptotic and late apoptotic cells percentages); (d) The intracellular ROS generation of A2780 cells under different treatment conditions as above; (e) The intracellular ROS generation of SK‐OV‐3 cells under different treatment conditions as above. ****, *p* < 0.0001. Error bars represent mean ± SD. Scale bar: 100 μm. For clarity, **OBB** NPs are abbreviated as **OBB** in Figure 5.

Phototherapy induces two main forms of cell death: programmed apoptosis and disintegrative necrosis.^[57]^ The mechanism by which the novel **OBB** NPs induce cell death is currently under investigation. Therefore, we performed flow cytometry on A2780 and SK‐OV‐3 cells using Annexin V‐FITC/PI as fluorescent markers to distinguish these stages of cell apoptosis caused by **OBB** NPs (Figure 5a).^[58]^ Annexin V staining indicated the early stage of cell apoptosis, and PI staining indicated the late apoptosis or dead cells. We noticed that the apoptosis percentage of **OBB** NPs + light group on A2780 cells exceed 18.9 ± 0.9% (Figure 5b), which was mainly in the early and late apoptosis stages. While the apoptosis rate in SK‐OV‐3 cells treated with **OBB** NPs + light was 22.4 ± 1.3% (Figure 5c), most of these dead cells were in the late apoptosis or necrosis stage. The data overall indicated that **OBB** NPs are effective to induce apoptosis and necrosis in ovarian cancer cells.

To investigate ROS induction by **OBB** NPs in ovarian cancer cells, cellular ROS levels were measured across different treatment groups. As illustrated in Figure 5d–e, the control group exhibited negligible ROS levels. The Cells (A2780 and SK‐OV‐3) treated with either **OBB** NPs alone or light irradiation alone displayed very low levels of ROS. In contrast, cells treated with **OBB** NPs plus light irradiation (**OBB** NPs + light) produced extremely high levels of ROS (Figure S22). These findings demonstrate that ROS‐mediated oxidative stress constitutes a key mechanism for **OBB** NP‐induced phototherapeutic cell death.

## 3 CONCLUSION

Based on the comprehensive findings of this study, we conclusively demonstrate that the strategic incorporation of a *meso*‐CF_3_ group and furan/thiophene heterocycles into BODIPY dyes enables precise control over their molecular packing through intermolecular hydrogen bonding (C‐H⋯F). These specific non‐covalent interactions, with bond distances critically ranging between 2.336 Å and 2.542 Å, direct the formation of well‐defined nanostructures such as hydrogen‐bonded associates. This controlled aggregation behavior is instrumental in achieving desirable photophysical properties, including a significant bathochromic shift of absorption into the NIR‐II window and broadened absorption bands, thereby facilitating efficient light harvesting and laser matching. Remarkably, the optimized nanoparticle formulation, **OBB** NPs, exhibits outstanding performance as a phototherapeutic agent, characterized by a high PCE (49.7%), a large Stokes shift, and potent Type‐I ROS generation capability. The efficacy of this system is underscored by its exceptional phototoxicity against ovarian cancer cells, inducing cell death primarily via an ROS‐mediated apoptotic and necrotic pathway while maintaining minimal dark toxicity. This work successfully establishes a fundamental structure‐property relationship that highlights the pivotal role of directed intermolecular hydrogen bonding in tailoring the aggregation and function of organic nanomaterials. The insights and design principles elucidated here provide a robust platform for the future development of highly effective aggregation‐engineered phototheranostic agents for precision cancer therapy.

## CONFLICT OF INTEREST STATEMENT

The authors declare no conflicts of interest.

## ETHICS STATEMENT

No animal or human experiments were involved in this study.

## Supporting information

Supporting Information S1

## Data Availability

The data that supports the findings of this study are available in the supplementary material of this article.

## References

[1] B. H. Li , M. Y. Zhao , L. S. Feng , C. R. Dou , S. W. Ding , G. Zhou , L. F. Lu , H. X. Zhang , F. Y. Chen , X. M. Li , G. F. Li , S. C. Zhao , C. Y. Jiang , Y. Wang , D. Y. Zhao , Y. S. Cheng , F. Zhang , Nat. Commun. 2020, 11, 3102. 32555157 10.1038/s41467-020-16924-z PMC7303218

[2] L. Yuan , W. Y. Lin , K. B. Zheng , L. W. He , W. M. Huang , Chem. Soc. Rev. 2013, 42, 622. 23093107 10.1039/c2cs35313j

[3] D. Ding , B. Z. Tang , Adv. Healthc. Mater. 2021, 10, e2102499. 34935308 10.1002/adhm.202102499

[4] J. Wang , P. R. Wang , Y. F. Li , X. W. Cheng , S. Y. Zhou , S. Han , J. B. Sun , C. H. Sun , ACS Macro Lett. 2025, 14, 1081. 40673391 10.1021/acsmacrolett.5c00362

[5] M. M. Bogner , G. London , Nat. Synth. 2024, 3, 1317.

[6] D. Zhang , L. Liu , X. Zhang , J. Lu , X.‐D. Jiang , Res. Chem. Mater. 2024, 3, 103.

[7] S. M. Atyabi , H. R. Shamlouei , E. Asgari , G. M. Roozbahani , Russ. J. Phys. Chem. B 2021, 15, S160.

[8] C. Q. Han , J. X. Guo , S. Sun , Z. Y. Wang , L. Wang , X. Y. Liu , Small 2024, 20, e2405887. 39248647 10.1002/smll.202405887

[9] A. Kreft , A. Lücht , J. Grunenberg , P. G. Jones , D. B. Werz , Angew. Chem. Int. Ed. 2019, 58, 1955. 10.1002/anie.201812880 30561872

[10] S. Y. Go , H. Chung , S. J. Shin , S. An , J. H. Youn , T. Y. Im , J. Y. Kim , T. D. Chung , H. G. Lee , J. Am. Chem. Soc. 2022, 144, 9149. 35575552 10.1021/jacs.2c03213

[11] Z. F. Zhao , W. J. Yan , W. B. Zheng , L. P. Guo , R. P. Yu , M. H. Chen , H. J. Zheng , Small 2025, 21, e2412626. 40079113 10.1002/smll.202412626

[12] S. M. Wu , W. Z. Zhang , C. R. Li , Z. G. Ni , W. F. Chen , L. Z. Gai , J. W. Tian , Z. J. Guo , H. Lu , Chem. Sci. 2024, 15, 5973. 38665518 10.1039/d3sc06976a PMC11040637

[13] F. Würthner , T. E. Kaiser , C. R. Saha‐Möller , Angew. Chem. Int. Ed. 2011, 50, 3376. 10.1002/anie.201002307 21442690

[14] O. Chovnik , S. R. Cohen , I. Pinkas , L. Houben , T. E. Gorelik , Y. Feldman , L. J. W. Shimon , M. A. Iron , M. Lahav , M. E. van der Boom , ACS Nano 2021, 15, 14643. 34516094 10.1021/acsnano.1c04355

[15] Q. Cai , R. Guo , D. F. Chen , Z. X. Deng , J. T. Gao , J. Nanobiotechnology. 2025, 23, 178. 40050980 10.1186/s12951-025-03254-9 PMC11884119

[16] C. H. Chen , S. D. Zhang , Acc. Chem. Res. 2025, 58, 583. 39873624 10.1021/acs.accounts.4c00754

[17] Y. T. Li , H. F. Lu , L. H. Lu , H. M. Wang , Acc. Mater. Res. 2025, 6, 447.

[18] T. K. Maji , ACS Appl. Mater. Interfaces 2023, 15, 25079. 37259285 10.1021/acsami.3c05952

[19] S. Y. Yun , D. Seo , H.‐J. Kim , D.‐J. Jeung , Y. K. Jeong , J.‐M. Oh , J. K. Park , J. Ind. Eng. Chem. 2021, 95, 28.

[20] P. Liesfeld , Y. Garmshausen , S. Budzak , J. Becker , A. Dallmann , D. Jacquemin , S. Hecht , Angew. Chem. Int. Ed. 2020, 59, 19352. 10.1002/anie.202008523 PMC7589249 32720745

[21] C. H. Suresh , S. Anila , Acc. Chem. Res. 2023, 56, 1884. 37351926 10.1021/acs.accounts.3c00193

[22] H. Kang , W. Lee , J. Oh , T. Kim , C. Lee , B. J. Kim , Acc. Chem. Res. 2016, 49, 2424. 27753477 10.1021/acs.accounts.6b00347

[23] B. L. Green , A. T. Collins , C. M. Breeding , Mineral. Geochem. 2022, 88, 637.

[24] J. H. Kim , T. Schembri , D. Bialas , M. Stolte , F. Würthner , Adv. Mater. 2022, 34, e2104678. 34668248 10.1002/adma.202104678

[25] S. Ogi , C. Grzeszkiewicz , F. Würthner , Chem. Sci. 2018, 9, 2768. 29732062 10.1039/c7sc03725b PMC5914135

[26] C. C. Teng , H. P. Dang , Y. X. Xu , D. L. Yin , L. F. Yan , Adv. Healthc. Mater. 2023, 12, e2300541. 37118995 10.1002/adhm.202300541

[27] C. Li , J. l. Chen , T. T. Man , B. Chen , J. Li , Q. Li , X. R. Yang , Y. Wan , C. H. Fan , J. L. Shen , JACS Au 2024, 4, 1125. 38559725 10.1021/jacsau.3c00826 PMC10976577

[28] X. Xiang , H. Q. Pang , T. Ma , F. X. Du , L. Li , J. B. Huang , L. Ma , L. Qiu , J. Nanobiotechnology. 2021, 19, 92. 33789692 10.1186/s12951-021-00835-2 PMC8011114

[29] K. Yu , K. S. Schanze , A. C. S. Cent , Sci 2023, 9, 1989. 10.1021/acscentsci.3c01296 PMC10683490 38033796

[30] A. Loudet , K. Burgess , Chem. Rev. 2007, 107, 4891. 17924696 10.1021/cr078381n

[31] R. Ziessel , G. Ulrich , A. Harriman , New J. Chem. 2007, 31, 496.

[32] C. I. Ezugwu , B. Mousavi , M. A. Asraf , Z. Luo , F. Verpoort , J. Catal. 2016, 344, 445.

[33] M. Y. Jia , Y. Wang , Y. Liu , L. Y. Niu , L. Feng , Biosens. Bioelectron. 2016, 85, 515. 27209578 10.1016/j.bios.2016.05.029

[34] Z. Ruan , P. Yuan , T. W. Li , Y. L. Tian , Q. Cheng , L. F. Yan , Eur. J. Pharm. Biopharm. 2019, 135, 25. 30550923 10.1016/j.ejpb.2018.12.006

[35] T. Zhang , C. Ma , T. T. Sun , Z. G. Xie , Coord. Chem. Rev. 2019, 390, 76.

[36] M. Raza , M. Jawaid , B. Abu‐Jdayil , Int. J. Biol. Macromol. 2025, 294, 139539. 39778835 10.1016/j.ijbiomac.2025.139539

[37] F. Berrellez‐Reyes , B. Schiavo , B. Gonzalez‐Grijalva , A. Angulo‐Molina , D. Meza‐Figueroa , Environ. Pollut. 2025, 364, 125314. 39547557 10.1016/j.envpol.2024.125314

[38] Z. Najafi , C. J. F. Kahn , F. Bildik , E. Arab‐Tehrany , N. Şahin‐Yeşilçubuk , Int. J. Biol. Macromol. 2021, 188, 62. 34343589 10.1016/j.ijbiomac.2021.07.175

[39] R. R. Zhang , K. Masenelli‐Varlot , T. Epicier , D. Stauffer , F. Chaput , L. Joly‐Pottuz , Mater. Today Nano 2025, 29, 100564.

[40] B. K. Wilson , R. K. Prud'homme , J. Colloid Interface Sci. 2021, 604, 208. 34265681 10.1016/j.jcis.2021.04.081

[41] S. K. Pandey Aashima , S. Singh , S. K. Mehta , J. Colloid Interface Sci. 2018, 529, 496. 29945019 10.1016/j.jcis.2018.06.030

[42] J. F. Hou , J. J. Jie , X. W. Wei , X. Q. Shen , Q. F. Zhao , X. P. Chai , H. Pang , Z. R. Shen , J. Q. Wang , L. P. Wu , J. H. Xu , J. Nanobiotechnol. 2024, 22, 449. 10.1186/s12951-024-02675-2 PMC11287882 39080658

[43] K. Dhangar , M. Kumar , M. Aouad , J. Mahlknecht , N. P. Raval , Chemosphere 2023, 311, 137088. 36332736 10.1016/j.chemosphere.2022.137088

[44] S. Attia , M. C. Schmidt , C. Schröder , S. Schauermann , ACS Catal. 2019, 9, 6882.

[45] A. P. Deshmukh , W. Zheng , C. Chuang , A. D. Bailey , J. A. Williams , E. M. Sletten , E. H. Egelman , J. R. Caram , Nat. Chem. 2024, 16, 800. 38316987 10.1038/s41557-023-01432-6 PMC11088501

[46] M. B. Miltenburg , T. B. Schon , E. L. Kynaston , J. G. Manion , D. S. Seferos , Chem. Mater. 2017, 29, 6611.

[47] F. Neese , Wires. Comput. Mol. Sci. 2025, 15, e70019.

[48] H. Song , N. K. Szymczak , Angew. Chem. Int. Ed. 2024, 63, e202411099. 10.1002/anie.202411099 38967599

[49] Y. Liu , P. Bhattarai , Z. Dai , X. Chen , Chem. Soc. Rev. 2019, 48, 2053. 30259015 10.1039/c8cs00618k PMC6437026

[50] H. W. Chang , S. U. Jen , Y. H. Liao , F. C. Chang , W. C. Chang , C. H. Chiu , J. Cifre , D. G. Chubov , I. S. Golovin , J. Alloys Compd. 2022, 927, 166894.

[51] G. H. Zhang , H. L. Wang , L. Cheng , Y. L. Li , Z. H. Zhu , H. H. Zou , J. Colloid Interface Sci. 2025, 679, 578. 10.1016/j.jcis.2024.10.133 39471586

[52] D. Y. Xiao , C. X. Wu , B. F. Liang , S. Z. Jiang , J. X. Ma , Y. Li , J. Mater. Chem. A. 2024, 12, 31655.

[53] A. Molla , M. Sahu , S. Hussain , J. Mater. Chem. A. 2015, 3, 15616.

[54] H. Mousa , J. Naser , J. Energy Storage 2019, 25, 100871.

[55] F. Wu , J. Zhang , J. T. Ye , J. W. Wang , W. H. Zheng , H. Chen , X. Y. Dong , Z. S. Huang , L. Y. Cai , G. H. Xiang , J. Nanobiotechnol. 2025, 23, 503. 10.1186/s12951-025-03570-0 PMC12255031 40652210

[56] L. J. Tan , R. Huang , X. Q. Li , S. P. Liu , Y. M. Shen , Acta Biomater. 2025, 196, 537. 39929764 10.1016/j.actbio.2025.01.063

[57] J. Y. Yuan , D. Ofengeim , Nat. Rev. Mol. Cell Biol. 2024, 25, 379. 38110635 10.1038/s41580-023-00689-6

[58] L. V. G. Longo , B. McNeil‐Laidley , F. Cottini , T. Hughes , G. Hilinski , E. Merritt , D. M. Benson , Blood 2021, 138, 2660. 10.3324/haematol.2023.282838 PMC10828771 37496433

