# Supplementary material for: Intermolecular hydrogen‐bonded associates of BODIPYs drive controllable aggregates and enhanced NIR phototherapeutic performance
Source: Smart Mol. 2026 Mar 11;4(2):e70041. doi: 10.1002/smo2.70041 (PMC13317655; doi:10.1002/smo2.70041)
Supplement: Supplementary file 1 — Supporting Information S1 [file SMO2-4-e70041-s001.docx]

**Supporting Information For**

**Intermolecular Hydrogen-Bonded Associates of BODIPYs Drive Controllable Aggregates and Enhanced NIR Phototherapeutic Performance**

**Siying Gou,**^a,1^ **Zhe Xun,**^b,1^ **Peijun Yang,**^a, 1^ **Xin Li,**^c^ **Dongxiang Zhang,**^a^ **Jianjun Du,**^c^ **Shuo Li,**^b,^* **Xin-Dong Jiang,**^a,^* **Gaowu Qin**^a,^*

^a^Institute for Strategic Materials and Components, Liaoning & Shenyang Key Laboratory of Functional Dye and Pigment, Shenyang University of Chemical Technology, Shenyang, 110142, China. E-mail: [xdjiang@syuct.edu.cn](mailto:xdjiang@syuct.edu.cn); gwqin@syuct.edu.cn

^b^Department of Biochemistry and Molecular Biology, School of Life Sciences, China Medical University, Shenyang, Liaoning 110122, China. E-mail: sli@cmu.edu.cn

^c^State Key Laboratory of Fine Chemicals, Dalian University of Technology, Dalian 116024, China.

^1^These authors contributed equally to this work.

**1. Experiment**

**2. NMR and HRMS**

**3. Figure and Table**

**4. X-ray data**

**1 Experimental section**

**General**

Unless specified otherwise, all of the organic solvents and chemicals used were analytical grade and purchased from Sinopharm Group Co. Ltd. without any further purification. A 400 MHz VARIAN Mercury NMR spectrometer was employed to record ^1^H NMR spectra. Chemical shifts in ^1^H NMR (δ) are provided in ppm downfield from Me_4_Si, as estimated by residual chloroform (δ 7.26 ppm). Using the internal chloroform signal at δ = 77.0 ppm as a reference, ^13^C NMR spectra were gathered in CDCl_3_ using a VARIAN Mercury 125 MHz spectrometer and reported in ppm. The exact molecular weight of the product is determined using a high-resolution mass spectrometer (Thermo Scientific LTQ Orbitrap XL). A UV-2550 spectrophotometer was utilized to record an absorption spectrum at 298 K. The fluorescence spectra were recorded with an F-98 spectrophotometer and are displayed as cm^-1^. A laser particle size analyzer was obtained from Malvern. A camera measuring temperature maintains track of the solution temperature. The 808 nm laser utilized for light irradiation was purchased from Changchun New Industries Optoelectronics Technology, and the output power was controlled via a fiber-linked laser system. The optical power density was measured using a CEL-NP 2000 power meter that was acquired from Beijing Zhong Jiao Jin Yuan Technology Co, Ltd. In the CCK8 assessment, the absorbance at 450 nm was quantified using a microplate reader (BioTek, Santa Clara, VT, USA). The Annexin V-FITC/PI Cell Apoptosis Detection Kit was bought from Wuhan Servicebio Technology Co., Ltd.

**Computational method**

The quantum chemical calculations in this work are performed by using the Gaussian 16 and Orca 6.0 programs. The density functional theory (DFT) and time-dependent density functional theory (TDDFT) calculations are carried out using the B3LYP functional and def2-TZVP basis set. Grimme’s D3-dispersion correction with Becke-Johnson damping (D3(BJ)) is employed to improve the description of the London-dispersion interactions. And the solvation effects of dichloromethane are also taken into consideration with the solvation model based on density (SMD).

**Singlet oxygen (^1^O_2_) detection**

Utilizing 1,3-diphenylisobenzofuran (DPBF), the ^1^O_2_ production in toluene was determined. The absorbance of DPBF at 416 nm was adjusted to about 1.4 in toluene and the absorbance of dye molecule was adjusted to about 0.8. The characteristic absorption of DPBF was applied to characterize ^1^O_2_ production. The absorption value of the dye molecule indicates the photo-stability of the dye. The 808 nm laser light source is used, the optical radiation power is 0.3 W/cm^2^.

**Reactive oxygen (ROS) detection**

10 μM DHR123 or 10 μM DCFH solution was prepared in DMSO and then mixed with nanoparticle dispersions of four different compounds at equal volumes to obtain the mixed solutions. Under irradiation with a 808 nm laser (0.3 W cm^2^), the fluorescence of the mixed solutions was measured after 1 minute of exposure, with the total irradiation time lasting 10 min.

**Calculation of photothermal efficiency**

The photothermal conversion efficiencies (*η*) was calculated using the following method:

$$\eta=\frac{hs\left( T_{Max}-T_{Surr} \right)-Q_{Dis}}{I(1-{10}^{-A})}$$

*h* means heat transfer coefficient, *s* was for container surface area, *Q_Dis_* stands for heat dispersed from the laser via the solvent and container, *I* was for laser power, and *A* represents for absorbance at excitation wavelength. *η* denotes photothermal conversion efficiency.

$$hs=\frac{mC}{\tau_{s}}$$

*m* is the total quantity of the photothermal reagent containing solution, *C* represents the temperature coefficient, and *τ*_s_ is the relevant time constant.

$$t=-\tau_{s}ln(\theta)$$

The temperature of the driving force is a non-dimensional parameter termed *θ*.

$$\theta=\frac{T-T_{Surr}}{T_{Max}-T_{Surr}}$$

*T* is the current temperature, *T_Max_* is the highest steady state temperature, and *T_surr_* denotes the surrounding temperature.

**The Preparation of nanoparticles (NPs)**

Nanoparticles were fabricated via the nano-deposition approach. In detail, 1 mL of THF solution with 1 mg dye and 5 mg DSPE-PEG_2000_ was slowly yet steadily introduced into 10 mL of aqueous solution. After that, THF was evaporated through constant stirring for 24 h to guarantee the homogeneous dispersion of the dye in the solution. The target products were collected by centrifuging the resulting nanoparticle mixture at 6000 rpm for 5 min.

**Cell lines**

Human ovarian cancer cells A2780 and SK-OV-3 were cultured in DMEM high glucose medium supplemented with 10% fetal bovine serum and 1% penicillin-streptomycin in a humidity-controlled atmosphere containing 5% CO_2_ at 37 ℃.

**Evaluation of cytotoxicity by CCK8 assay**

The cytotoxicity of **OBB** NPs against A2780 and SK-OV-3 cells was assessed using CCK8 assays. A2780 and SK-OV-3 cells were seeded into 96-well plates at 5 × 10³ cells per well and incubated overnight (37 °C, 5% CO₂). Cells were incubated with **OBB** NPs at various concentrations for 4 h, followed by irradiation using an 808 nm laser (0.3 W/cm²) for 5 min. 24 h post-irradiation, 10 µL of CCK8 solution was added to each well. After a 1 h incubation, absorbance was measured at 450 nm using a microplate reader following gentle shaking for 20 s. Cell viability was calculated relative to untreated control wells.

**Calcein-AM/PI staining of live-dead cells**

The phototherapeutic efficacy of **OBB** NPs was evaluated using Calcein-AM/PI staining to distinguish live/dead cells according to the manufacturer's protocol. Cells were divided into four treatment groups, Control: Untreated cells; **OBB** NPs: Cells incubated with **OBB** NPs (dark toxicity); Light alone: 808 nm irradiation (0.3 W/cm^2^, 5 min); **OBB** NPs + Light: 808 nm irradiation (0.3 W/cm^2^, 5 min) after 4h **OBB** NP incubation. A2780 and SK-OV-3 cells (2×10^4^ cells/well) were seeded in 24-well plates and incubated with **OBB** NPs for 4 h (40 μM for A2780, 50 μM for SK-OV-3). Following irradiation, all cultures were maintained for 24 h prior to adding Calcein-AM/PI double-stain solution. After 30 min incubation, fluorescence imaging was performed with the following parameters: Calcein-AM (green, Ex 488 nm/Em 500-529 nm) and PI (red, Ex 559 nm/Em 570-619 nm).

**Cell apoptosis analysis by Annexin V-FITC/PI**

Flow cytometry was used to detect the apoptotic ovarian cancer cells in photothermal therapy. A2780 and SK-OV-3 cells were processed using the same experimental groups as described above. Cells (2×10^5^ cells/well) were seeded in 6-well plates and incubated for 4 h with OBB NPs at cell line-specific concentrations (40 μM for A2780, 50 μM for SK-OV-3). Subsequently, cells were exposed to 808 nm laser irradiation (0.3 W/cm^2^) for 5 min. After 24 h incubation, cells were harvested via trypsinization and stained using an Annexin V-FITC/PI Apoptosis Detection Kit (Elabscience Biotechnology Co., Ltd.). Briefly, cells were resuspended in 100 μL of 1× binding buffer containing 2.5 μL Annexin V-FITC and 2.5 μL PI, followed by 20 min of dark incubation at room temperature. Immediately after adding 400 μL of 1× binding buffer, samples were analyzed on a BD LSRFortessa™ flow cytometer (BD Biosciences).

**Reactive oxygen species detection**

Intracellular ROS generation was measured by 5,6-chloromethyl-2′,7′-dichlorodihydrofluorescein diacetate (CM-H2DCFDA, Beyotime Biotechnology, S0035S). A2780 and SK-OV-3 were seeded in 24-well plates at 2×10^4^ cells/well and incubated overnight. A2780 and SK-OV-3 cells were processed using the same experimental groups as previously described. After treatments, intracellular ROS levels were measured by incubating cells with 5 μM CM-H_2_DCFDA at 37 °C for 30 min in the dark. Cells were then washed three times with serum-free medium. CM-DCF fluorescence was visualized with a fluorescence microscope (excitation: 488 nm; emission: 515–540 nm).

**Statistical analysis**

The Student’s t test (t test) and one way analysis of variance (ANOVA) were carried out by Graph Pad Prism version 8.0.2. All data reported herein represent the mean ± standard deviation (SD). *P*< 0.05 was considered statistically significant. * *P*<0.05, ** *P*<0.01, *** *P*< 0.001, **** *P*< 0.001.

**Synthesis**

**Scheme S1** Synthesis of BODIPYs.

1) *Synthesis of* ***OBB***

Acetic acid (0.1 mL) and piperidine (0.1 mL) were added to toluene (10 mL) solutions containing 5-bromo-2-furanoformaldehyde (1.105 g, 6.32 mmol) and **CF_3_-BDP** (0.5 g, 1.58 mmol). The mixture was stirred under reflux conditions for 5 h. After the mixture has cooled to room temperature, wash three times with water (100 mL), the organic phase was then extracted with CH_2_Cl_2_ (100 mL ×3). To obtain a crude product, the organic phase is dried with anhydrous sodium sulfate and then filtered. Subsequently, the solvent is removed by rotary evaporation. The crude product was purified by silica gel column chromatography to obtain a dark red solid **OBB** (0.298 g, 30%). ^1^H NMR (400 MHz, CDCl_3_) δ 7.55 (d, *J* = 15.6 Hz, 2H), 6.98 (d, *J* = 15.6 Hz, 2H), 6.72 (s, 2H), 6.59 (d, *J* = 3.6 Hz, 2H), 6.43 (d, *J* = 3.6 Hz, 2H), 2.35 (s, 6H). ^13^CNMR (125 MHz, CDCl_3_) δ 154.8, 153.8, 140.6, 136.1, 125.5, 123.5, 119.9, 117.1, 115.8, 114.3, 15.2. HRMS (ESI) m/z calcd for C_24_H_16_BBr_2_F_5_N_2_O_2_Na^+^ (M+Na)^+^652.94635, found 652.94611.

2) *Synthesis of* ***OHB***

Furan-2-carbaldehyde (0.607 g, 6.32 mmol) was used as the starting material (Scheme S1), and **OHB** was obtained as dark red solids (0.111 g, 15%). ^1^H NMR (400 MHz, CDCl_3_) δ 7.61 (d, *J* = 15.6 Hz, 2H), 7.54 (s, 2H), 6.98 (d, *J* = 15.6 Hz, 2H), 6.74 (s, 2H), 6.62 (s, 2H), 6.49 (s, 2H), 2.35 (s, 6H). ^13^C NMR (125 MHz, CDCl_3_) 153.9, 152.9, 144.7, 141.1, 134.3, 124.5, 120.7, 117.2, 113.4, 112.6, 77.3, 77.0, 76.7, 15.9, 15.8. HRMS (ESI) m/z calcd for C_24_H_18_BF_5_N_2_O_2_Na^+^ (M+Na)^+^495.12737, found 495.12722.

3) *Synthesis of* ***SBB***

5-bromothiophene-2-carboxaldehyde (1.207 g, 6.32 mmol) was used as the starting material (Scheme S1), and **SBB** was obtained as dark red solids (0.313 g, 30%). ^1^H NMR (400 MHz, CDCl_3_) δ 7.39 (d, *J* = 15.6 Hz, 2H), 7.29 (d, *J* = 15.6 Hz, 2H), 7.03 (d, *J* = 3.6 Hz, 2H), 7.01 (d, *J* = 3.6 Hz, 2H), 6.74 (s, 2H), 2.35 (s, 6H). ^13^C NMR (125 MHz, CDCl_3_) δ 153.5, 149.4, 143.7, 141.3, 134.3, 132.2, 131.2, 129.9, 129.8, 120.8, 118.4, 115.7, 77.2, 77.0, 76.8, 15.8, 15.8, 15.4, 14.1. HRMS (ESI) m/z calcd for C_24_H_16_BBr_2_F_5_N_2_S_2_Na^+^ (M+Na)^+^684.90066, found 684.90051.

4) *Synthesis of* ***SHB***

Thiophene-2-carbaldehyde (0.708 g, 6.32 mmol) was used as the starting material (Scheme S1), and **SHB** was obtained as dark red solids (0.119 g, 15%). ^1^H NMR (400 MHz, CDCl_3_) δ 7.52 (d, *J* = 16 Hz, 2H), 7.43 (d, *J* = 16 Hz, 2H), 7.39 (d, *J* = 3.6 Hz, 2H), 7.28 (d, *J* = 3.6 Hz, 2H), 7.08 (d, *J* = 3.6 Hz, 1H), 7.06 (d, *J* = 3.6 Hz, 1H), 6.72 (s, 2H), 2.35 (s, 6H). ^13^C NMR (125 MHz, CDCl_3_) δ 153.9, 153.7, 141.2, 134.5, 125.4, 123.4, 120.9, 117.3, 115.2, 114.7, 15.8. HRMS (ESI) m/z calcd for C_24_H_18_BF_5_N_2_S_2_Na^+^ (M+Na)^+^527.08168, found 527.08179.

**2 NMR and HRMS**


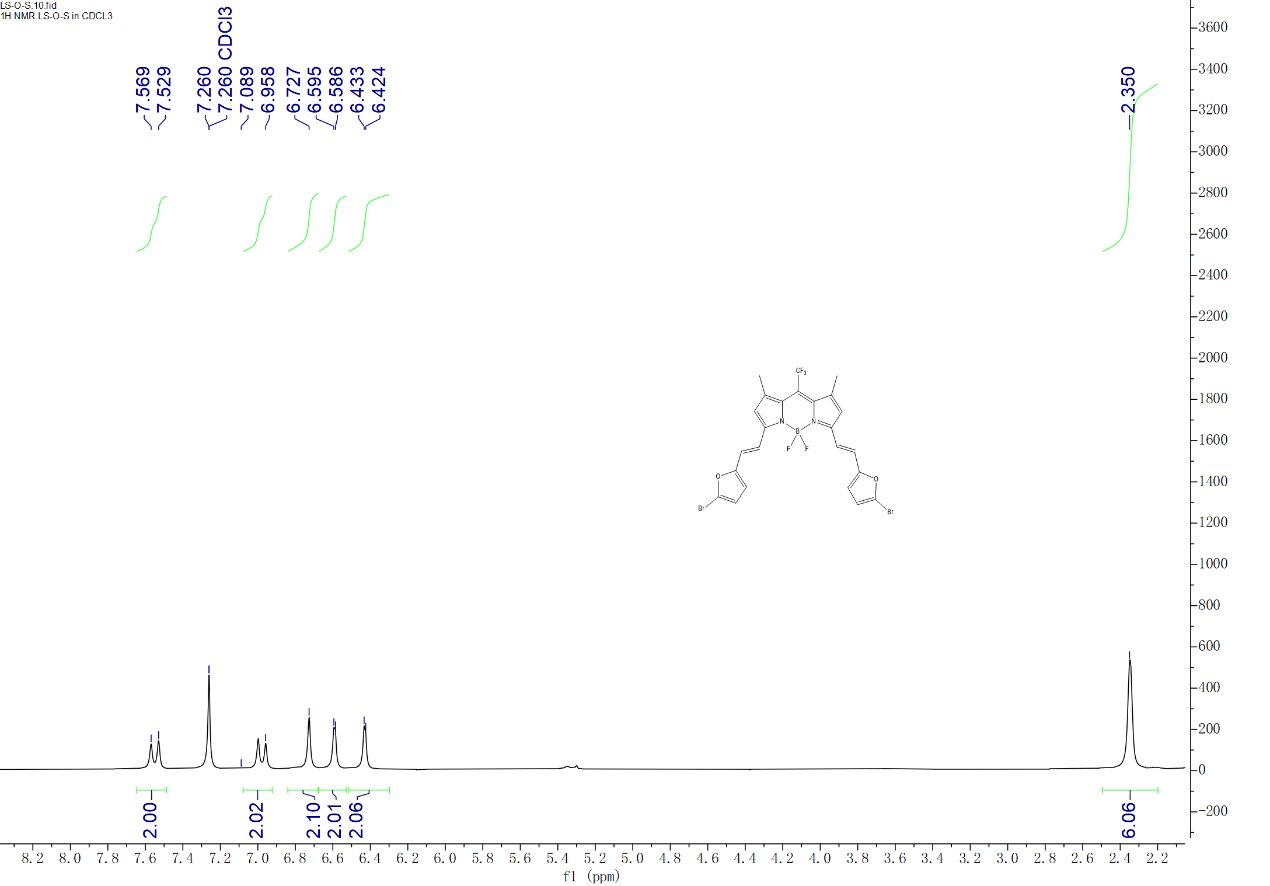


^1^H NMR spectrum (400 MHz, CDCl_3_) of **OBB**

**
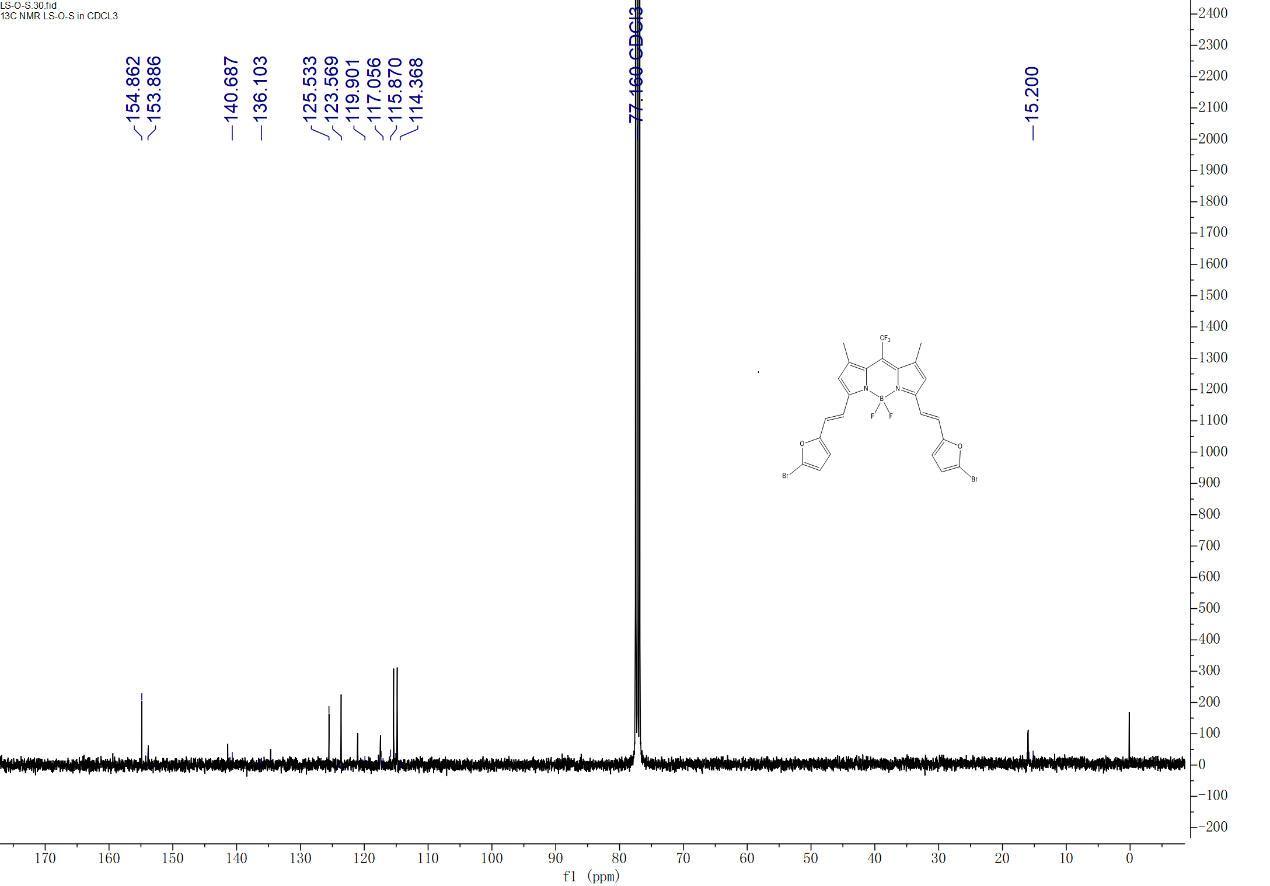
**

^13^C NMR spectrum (125 MHz, CDCl_3_) of **OBB**

**
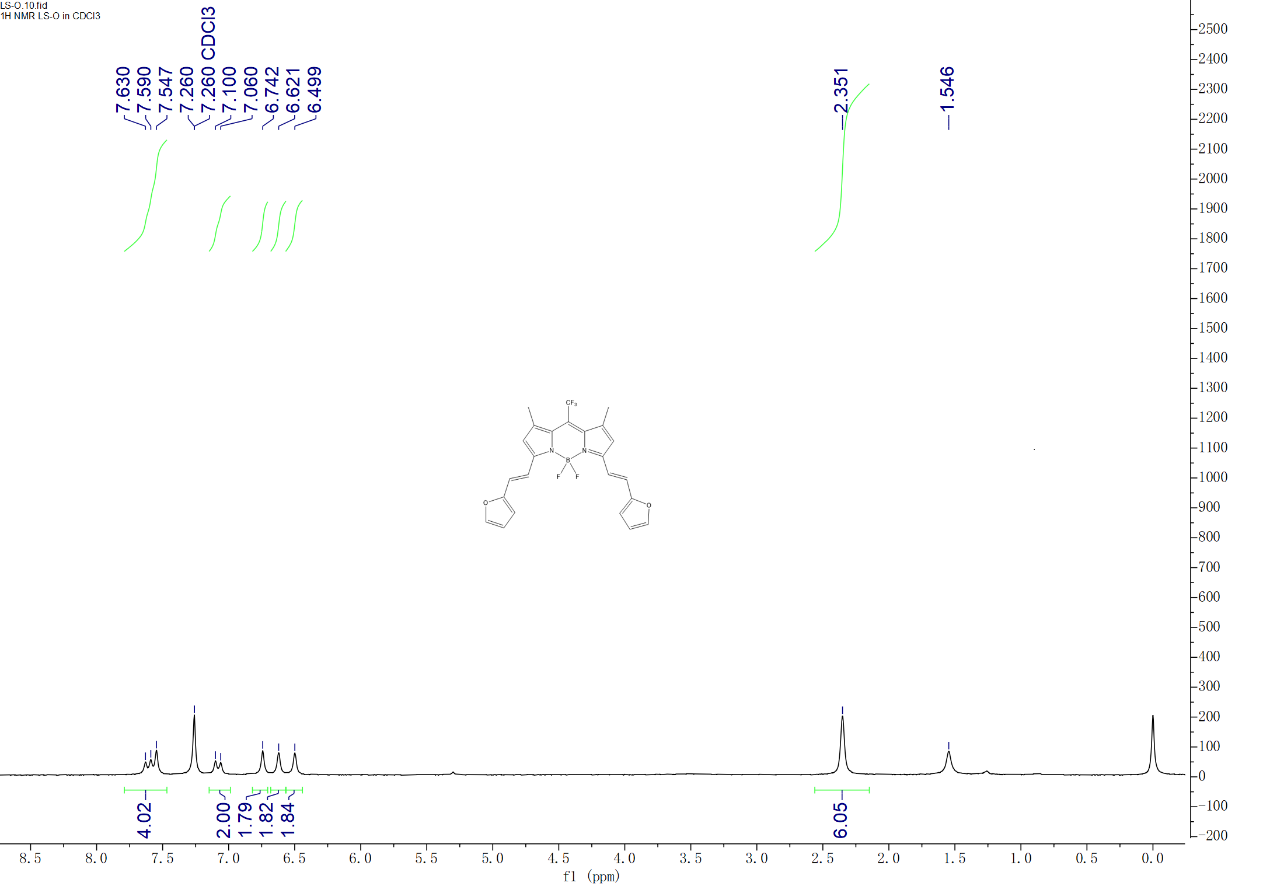
**

^1^H NMR spectrum (400 MHz, CDCl_3_) of **OHB**

*
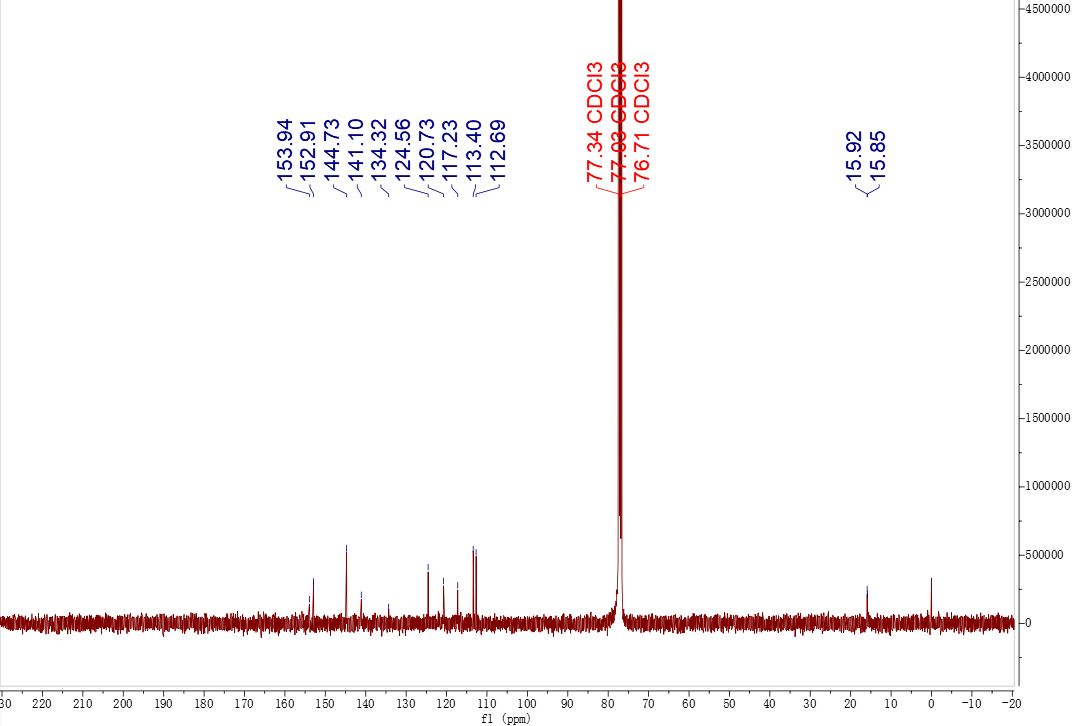
*

^13^C NMR spectrum (125 MHz, CDCl_3_) of **OHB**

**
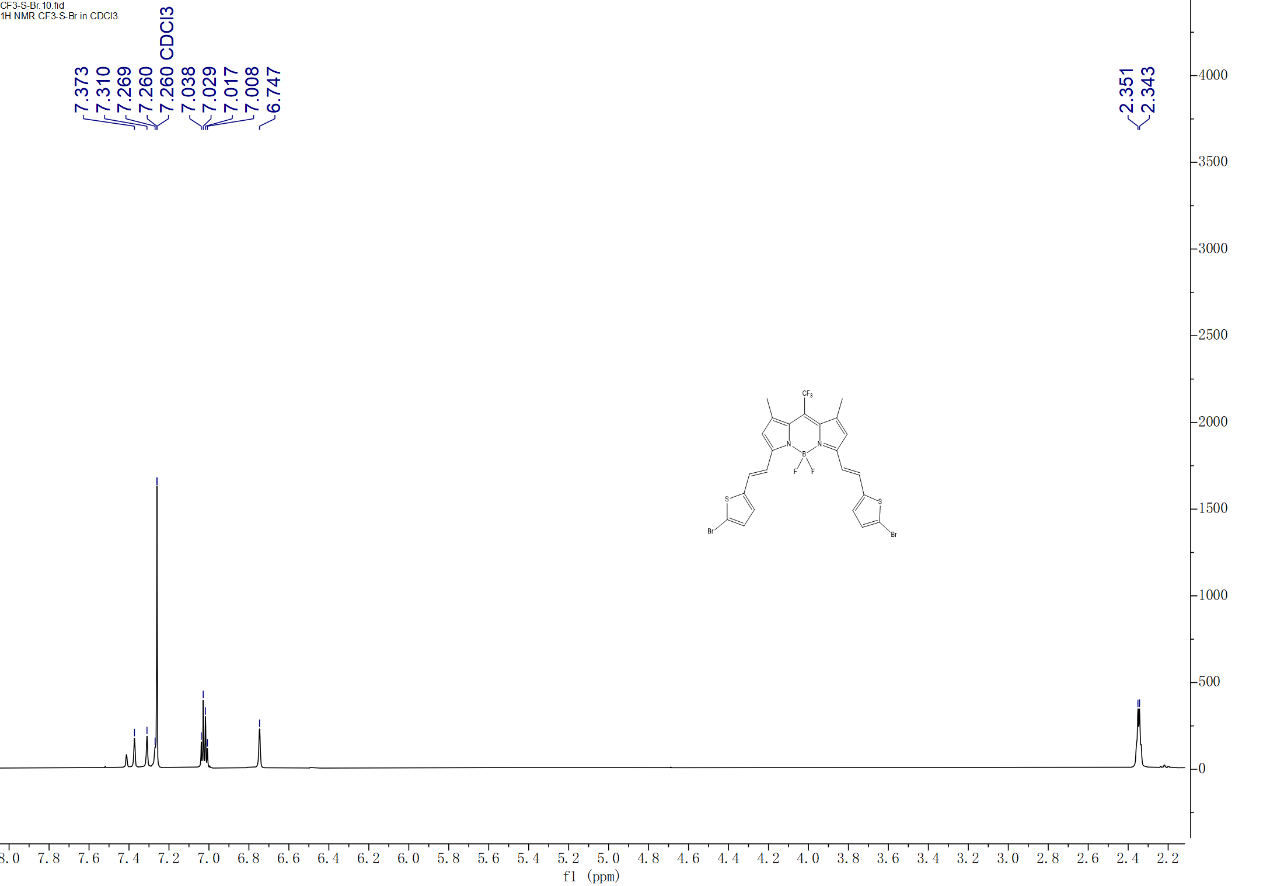
**

^1^H NMR spectrum (400 MHz, CDCl_3_) of **SBB**

**
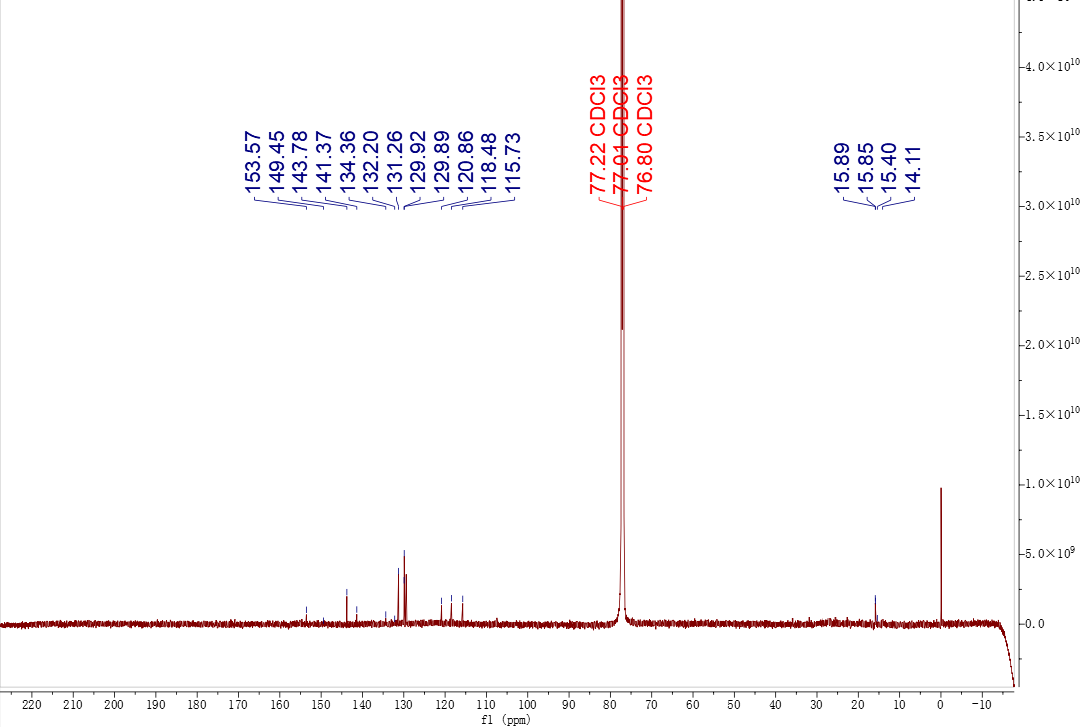
**

^13^C NMR spectrum (125 MHz, CDCl_3_) of **SBB**

^1^H NMR spectrum (400 MHz, CDCl_3_) of **SH-BDP**


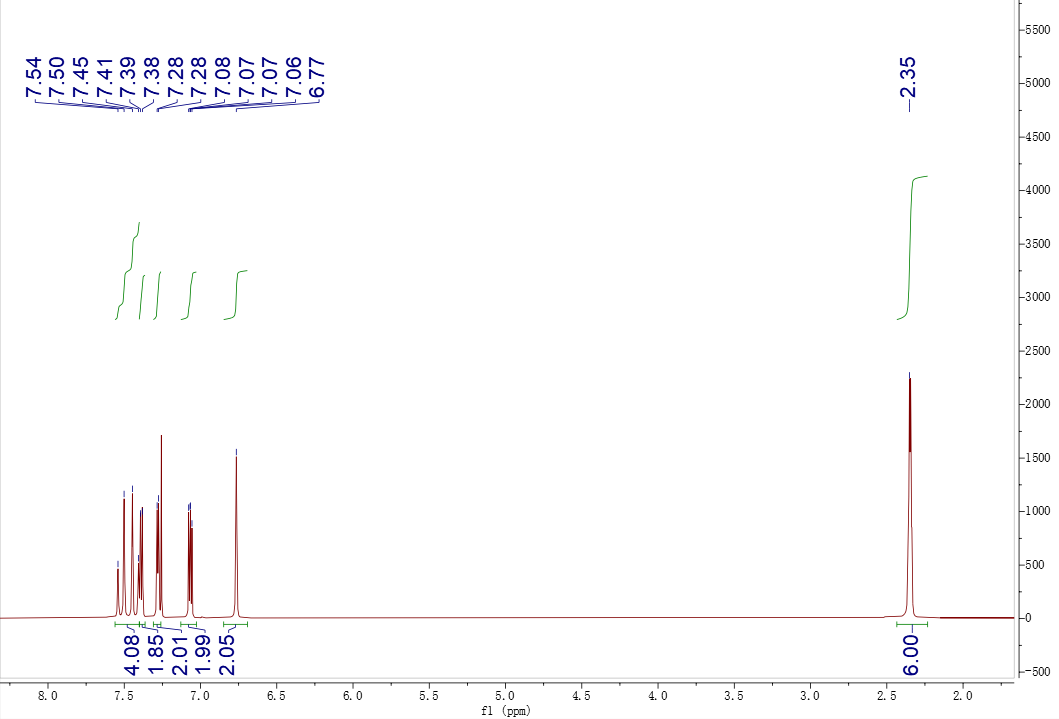


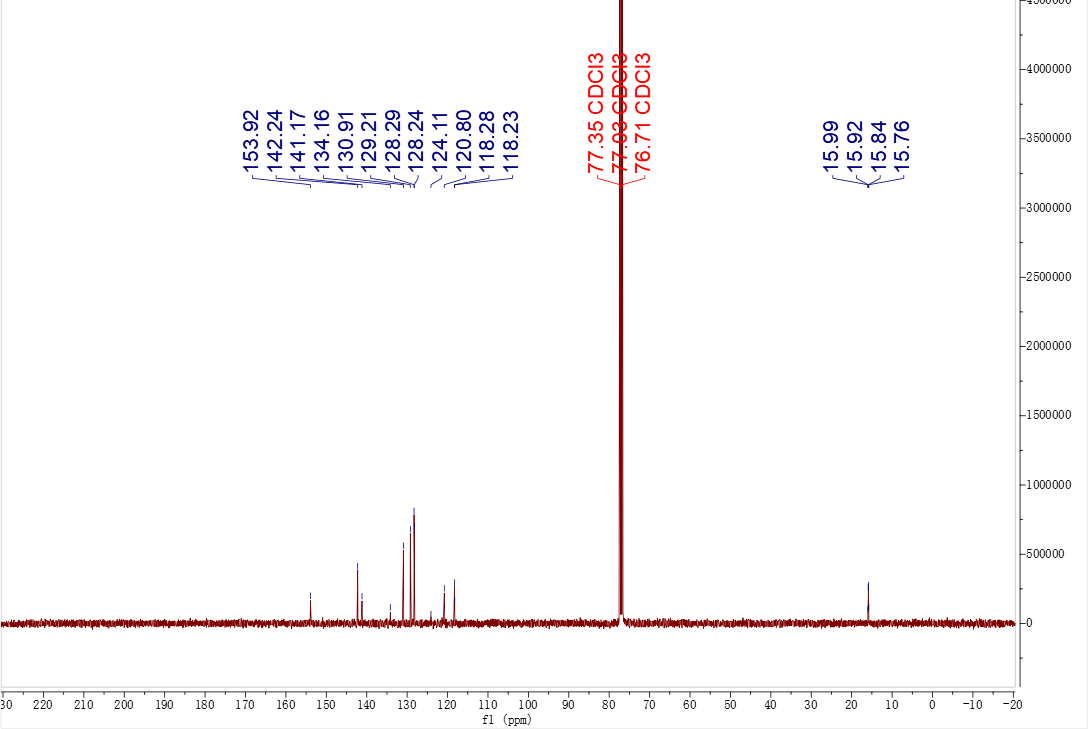


^13^C NMR spectrum (125 MHz, CDCl_3_) of **SHB**

**OBB:** HRMS (ESI) m/z calcd for C_24_H_16_BBr_2_F_5_N_2_O_2_Na^+^ (M+Na)^+^652.94635, found 652.94611.

**OHB:** HRMS (ESI) m/z calcd for C_24_H_18_BF_5_N_2_O_2_Na^+^ (M+Na)^+^495.12737, found 495.12722.

**SBB:** HRMS (ESI) m/z calcd for C_24_H_16_BBr_2_F_5_N_2_S_2_Na^+^ (M+Na)^+^684.90066, found 684.90051.

**SHB:** HRMS (ESI) m/z calcd for C_24_H_18_BF_5_N_2_S_2_Na^+^ (M+Na)^+^527.08168, found 527.08179.

**3. Figure and Table**

**
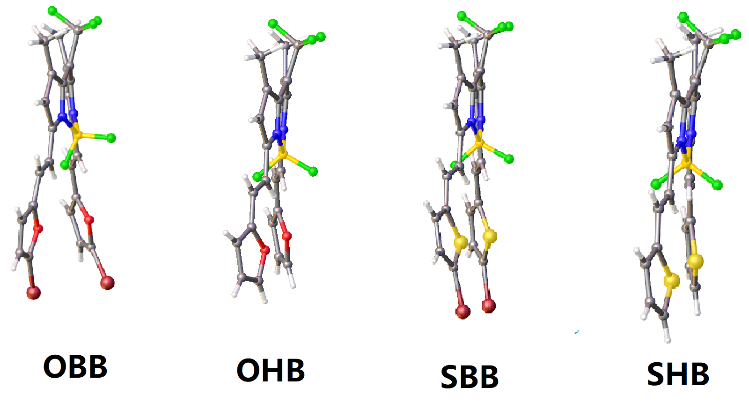
**

**FIGURE S1** Side views of the molecular structures.

**
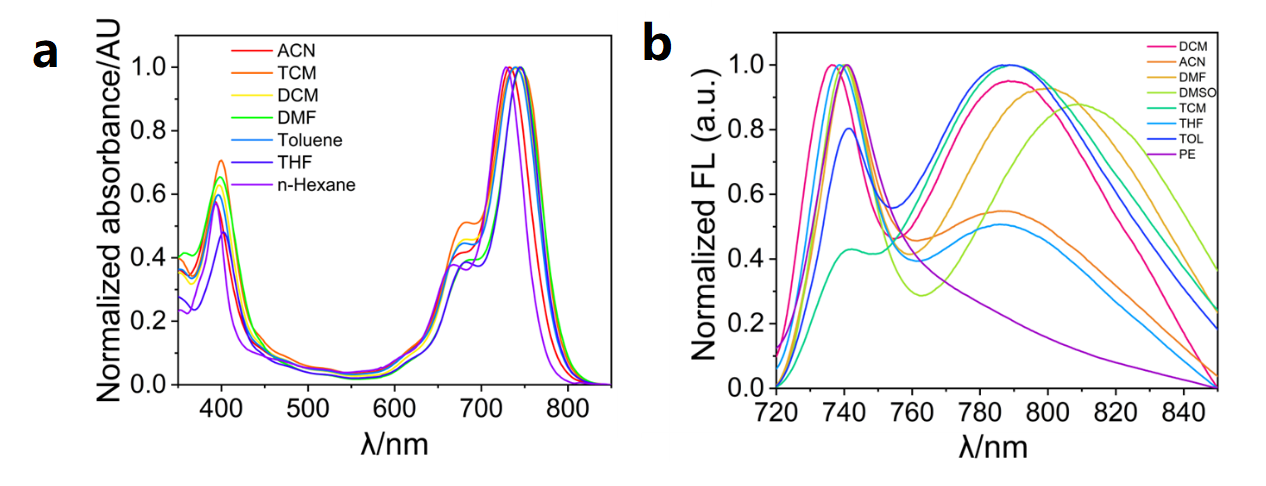
**

**
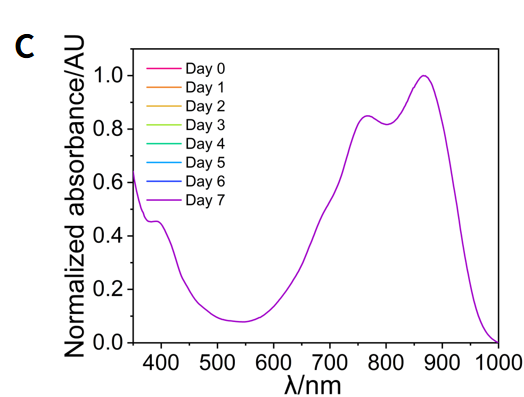
**

**FIGURE S2** a) Normalized absorption spectra and b) fluorescence intensity of **OBB** in different solvents. c) Absorption Changes of **OBB**-NPs in Aqueous Solution over a 7-Day Period.

**
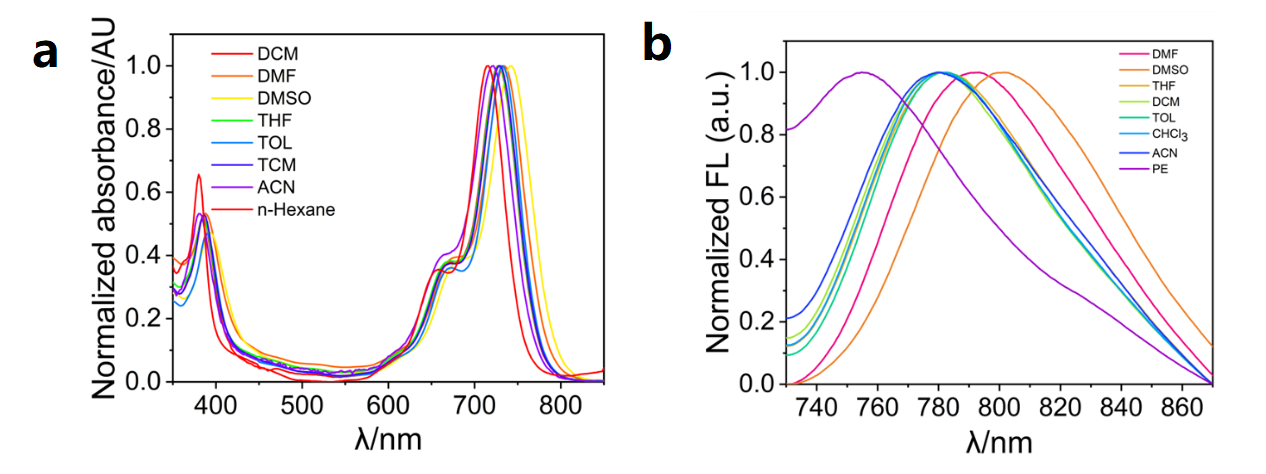
**

**FIGURE S3** a) Normalized absorption spectra and b) fluorescence intensity of **OHB** in different solvents.

**
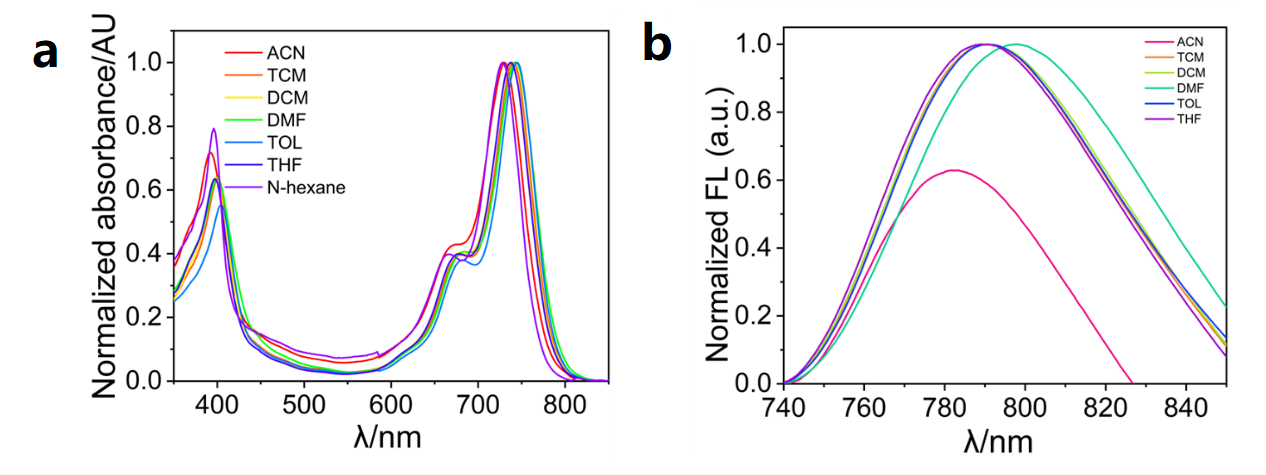
**

**FIGURE S4** a) Normalized absorption spectra and b) fluorescence intensity of **SBB** in different solvents.

**
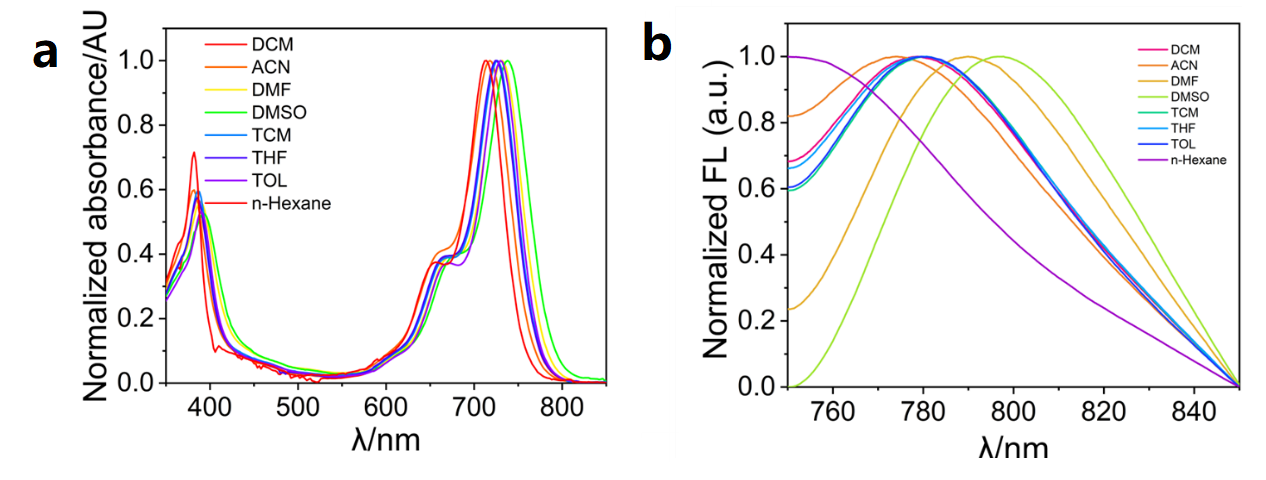
**

**FIGURE S5** a) Normalized absorption spectra and b) fluorescence intensity of **SHB** in different solvents.

**Table S1** Photophysical properties of **OBB** and **OHB**, **SBB** and **SHB** in various solvents at 298 K.

|  | **OBB** | | | | **OHB** | | | |
| --- | --- | --- | --- | --- | --- | --- | --- | --- |
| solvent | λ_abs_/λ_em_ | FWHM | Stokes shift |  | λ_abs_/λ_em_ | FWHM | Stokes shift |  |
| DCM | 738/788 | 50 | 38 |  | 728/780 | 68 | 30 |  |
| ACN | 732/800 | 68 | 30 |  | 722/780 | 58 | 32 |  |
| DMF | 744/788 | 44 | 30 |  | 732/792 | 60 | 32 |  |
| TCM | 740/790 | 50 | 42 |  | 728/780 | 52 | 34 |  |
| THF | 746/788 | 42 | 42 |  | 728/782 | 54 | 30 |  |
| TOL | 740/790 | 50 | 38 |  | 732/782 | 50 | 30 |  |
|  | **SBB** | | |  | **SHB** | | |  |
| solvent | λ_abs_/λ_em_ | FWHM | Stokes shift |  | λ_abs_/λ_em_ | FWHM | Stokes shift |  |
| DCM | 738/790 | 52 | 28 |  | 724/779 | 55 | 34 |  |
| ACN | 730/784 | 54 | 24 |  | 716/775 | 59 | 28 |  |
| DMF | 742/798 | 56 | 30 |  | 732/791 | 59 | 34 |  |
| TCM | 738/780 | 42 | 34 |  | 728/781 | 53 | 24 |  |
| THF | 740/789 | 49 | 30 |  | 724/780 | 56 | 28 |  |
| TOL | 744/792 | 48 | 28 |  | 730/780 | 50 | 28 |  |

**Table S2** Theoretical calculations of bimolecular and monomolecular absorption spectra at 298K

| **Bimolecular** | | | | **Single molecule** | | | |
| --- | --- | --- | --- | --- | --- | --- | --- |
|  | E(eV) | λ(nm) | f |  | E(eV) | λ(nm) | f |
| S1 | 0.92 | 1350.63 | 0.00 | **S1** | **1.87** | **664.55** | **0.77** |
| **S2** | **1.80** | **689.71** | **0.82** | S2 | 2.53 | 490.54 | 0.17 |
| **S3** | **1.83** | **677.52** | **0.74** | S3 | 2.95 | 420.52 | 0.00 |
| S4 | 1.91 | 648.19 | 0.01 | S4 | 3.06 | 404.59 | 0.04 |
| S5 | 1.97 | 628.98 | 0.00 | S5 | 3.19 | 388.24 | 0.00 |
| S6 | 2.46 | 504.72 | 0.07 | S6 | 3.38 | 366.79 | 1.38 |
| S7 | 2.48 | 499.37 | 0.03 | S7 | 3.52 | 352.54 | 0.00 |
| S8 | 2.52 | 491.55 | 0.16 | S8 | 4.10 | 302.34 | 0.00 |

**Table S3** Fluorescence quantum yield of monomer and *J* aggregate.

|  | **Fluorescence quantum yield of Single-molecule** | **Fluorescence quantum yield of *J*-aggregates** |
| --- | --- | --- |
| **OBB** | 0.005 | 0.002 |
| **OHB** | 0.014 | 0.007 |
| **SBB** | 0.003 | 0.001 |
| **SHB** | 0.011 | 0.003 |

**Table S4** Chemical structures and photophysical properties of the reported dye from literatures.

| Dye | Structure | λ_abs_/λ_em_ （nm） | *η* | Reference |
| --- | --- | --- | --- | --- |
| OMe-azaBDP |  | 688/724 | 36% | *Journal of Materials Chemistry B 12, 5 (2024): 1372-1378.* |
| Aza-BODIPY-mPEG |  | 827/- | 41% | *ACS Applied Bio Materials 5 (2022): 4567-4577.* |
| CN-NMe_2_ |  | 853/959 | - | *ChemPhotoChem 4 (2020): 5304-5311.* |
| DI-BDP-CZ | 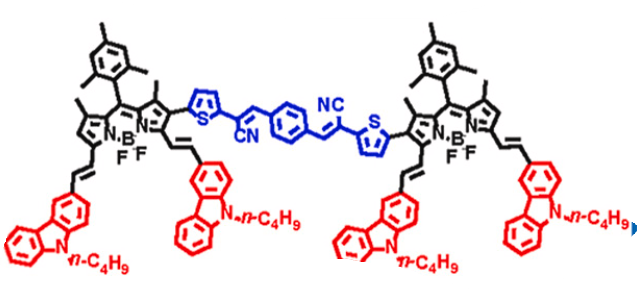 | 652/- | 14.97% | *International journal of stroke : official journal of the International Stroke Society, 13(6), 612–632.* |
| BDP-4PTZ |  | 615/- | 43% | *Chemical Research Chinese Universities 37 (2021): 951-959.* |
| BDP-26 | 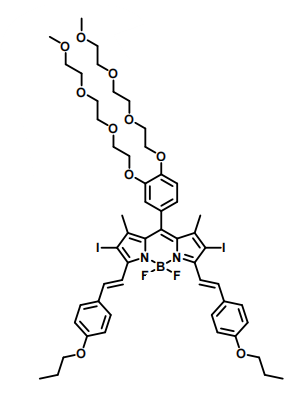 | 730/785 | 29% | *Journal of materials chemistry. B, 13(40), 12831–12868.* |
| NIR1028 |  | 913/1028 | - | *Journal of Nanobiotechnology 19 (2021): 431.* |
| BDP-b |  | 812/842 | - | *AngewandteChemie 63 (2024): e202319875.* |
| TTAB |  | 850/1000 | 35% | *Talanta 279 (2024): 126633.* |
| DA-azaBDP |  | 838/923 | 37.2% | *Chinese Chemical Letters (2024):* DOI 10.1016/j.cclet.2024.110098. |
| NBDPBr |  | 624/650 | 43% | *Chinese Chemical Letters 35 (2024): 108974.* |
| C8 -NBDP-OEG4 | 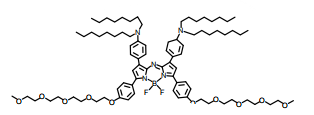 |  | 39.8% | *Biomaterials science, 13(19), 5369–5381.* |
| OBB |  | 740/788 | 49.7% | *This work* |

**
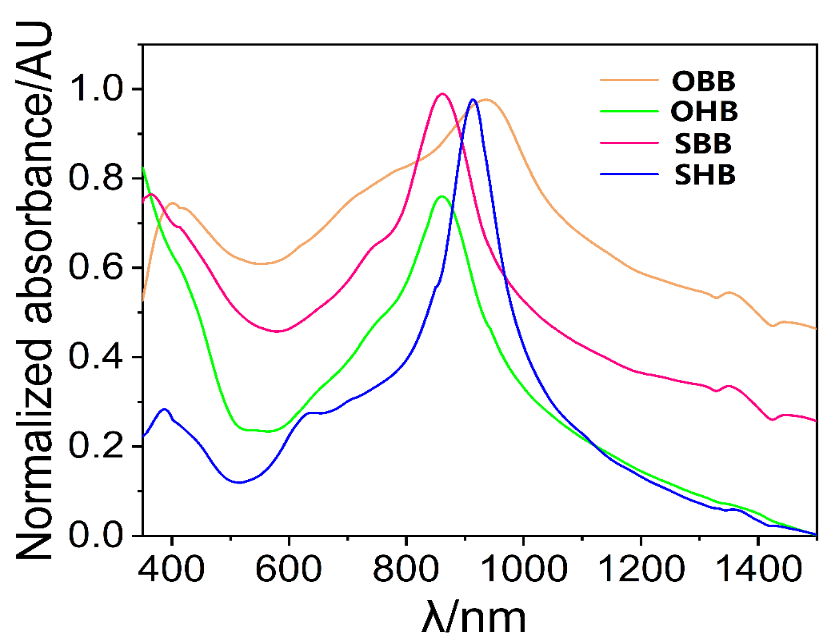
**

**FIGURE S6** Absorption spectra of four dyes in the film state.


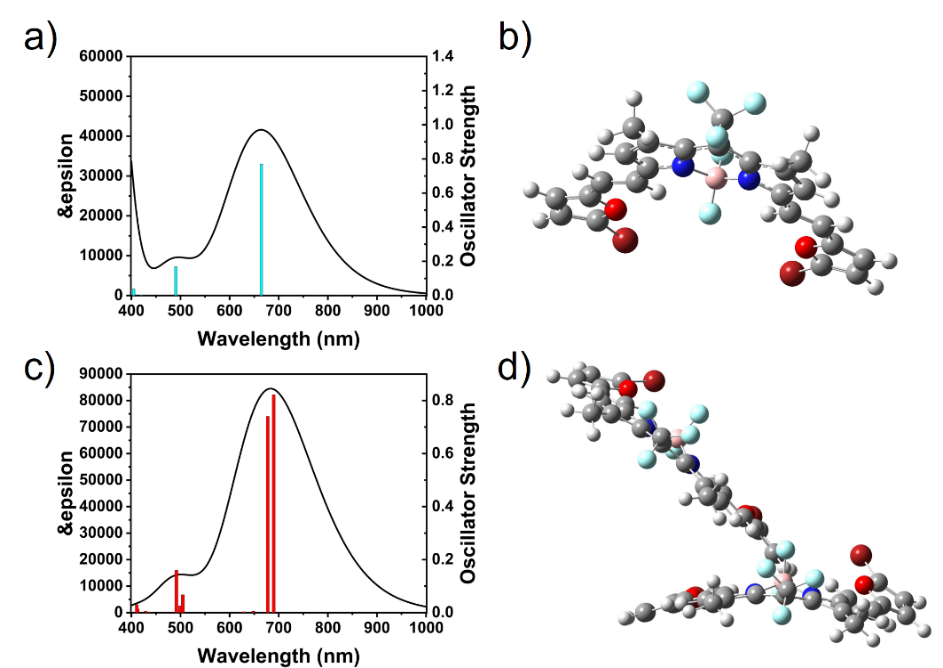


**FIGURE S7** a) Single-molecule theoretical calculations of absorption spectra. b) views of the single molecular structures. c) Bimolecular theory calculations of absorption spectra d) views of the bimolecular structures.

**
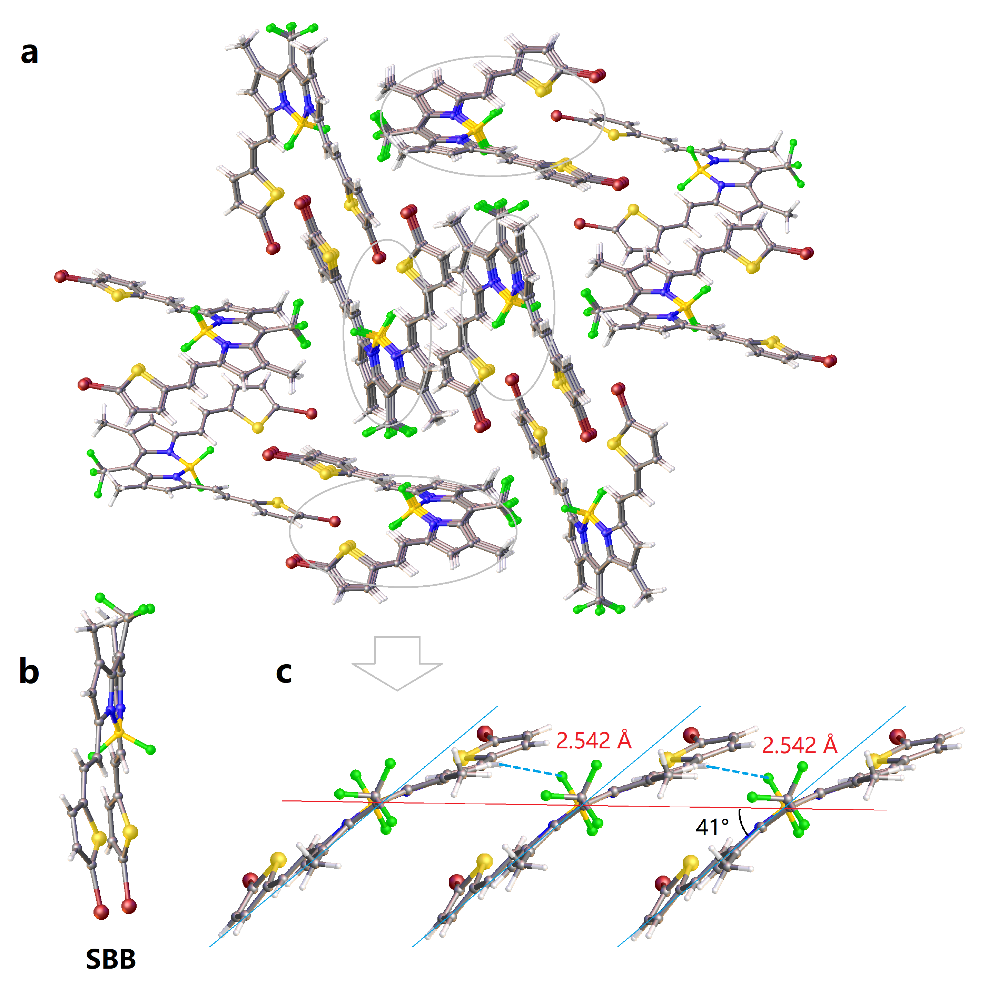
**

**FIGURE S8** Crystal packing of **SBB**.

**
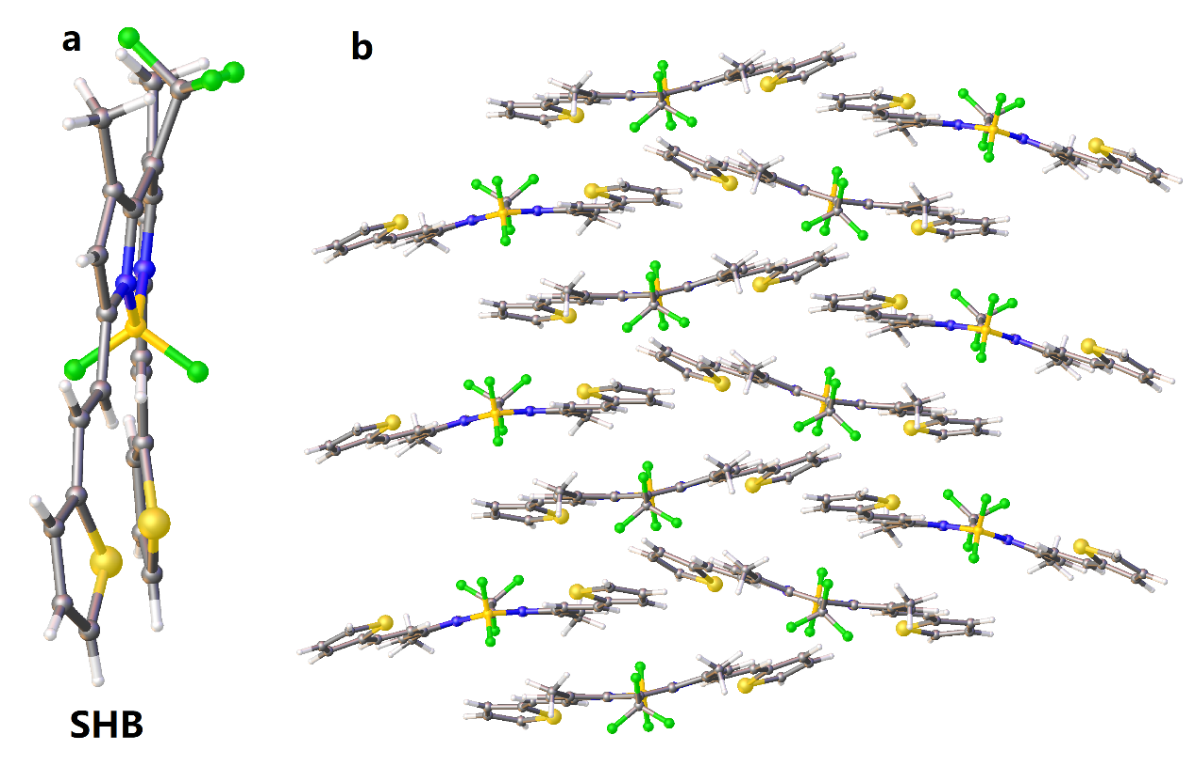
**

**FIGURE S9** Crystal packing of **SHB**.


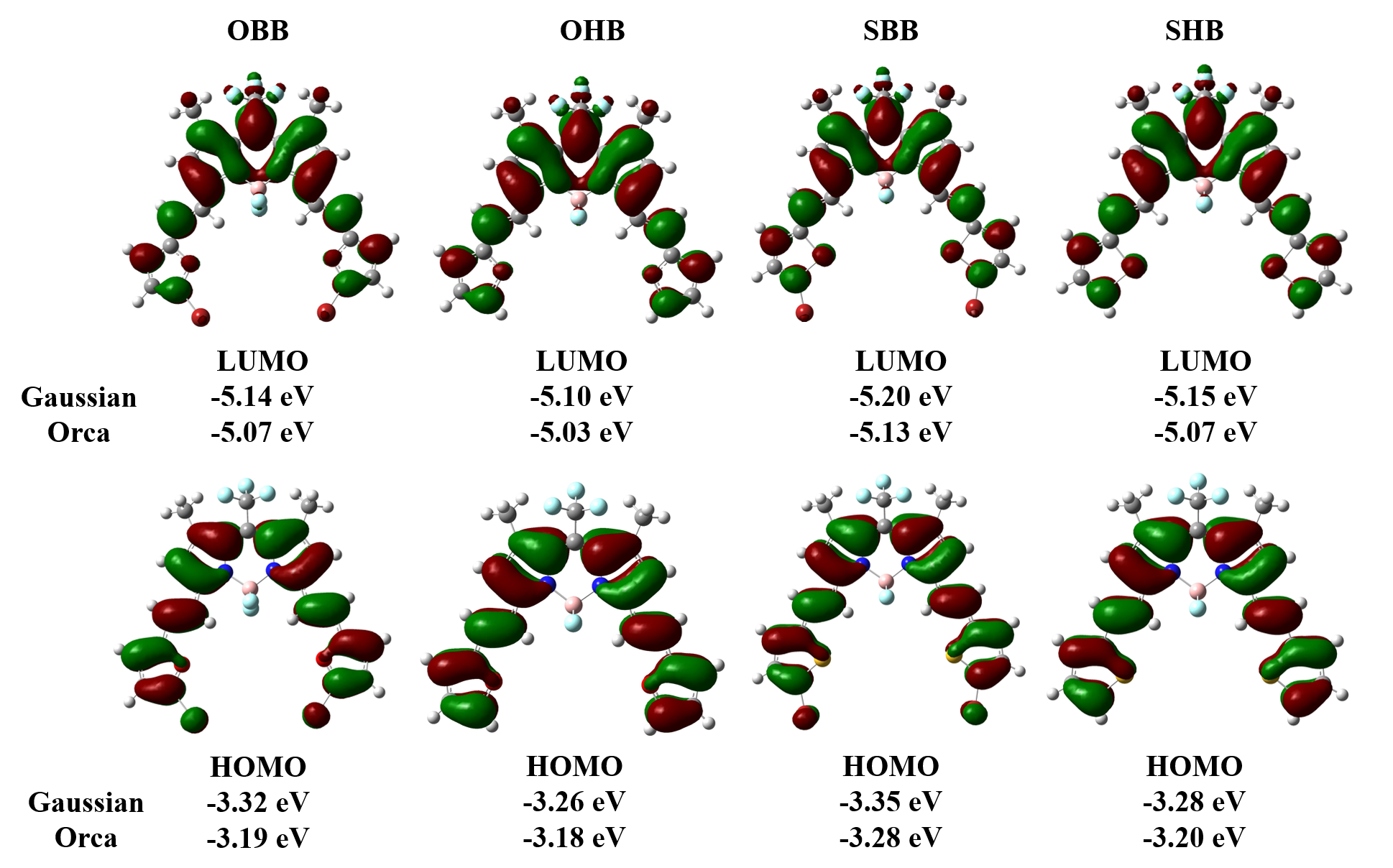


**FIGURE S10** Frontier orbitals of **OBB**, **OHB**, **SBB** and **SHB**.


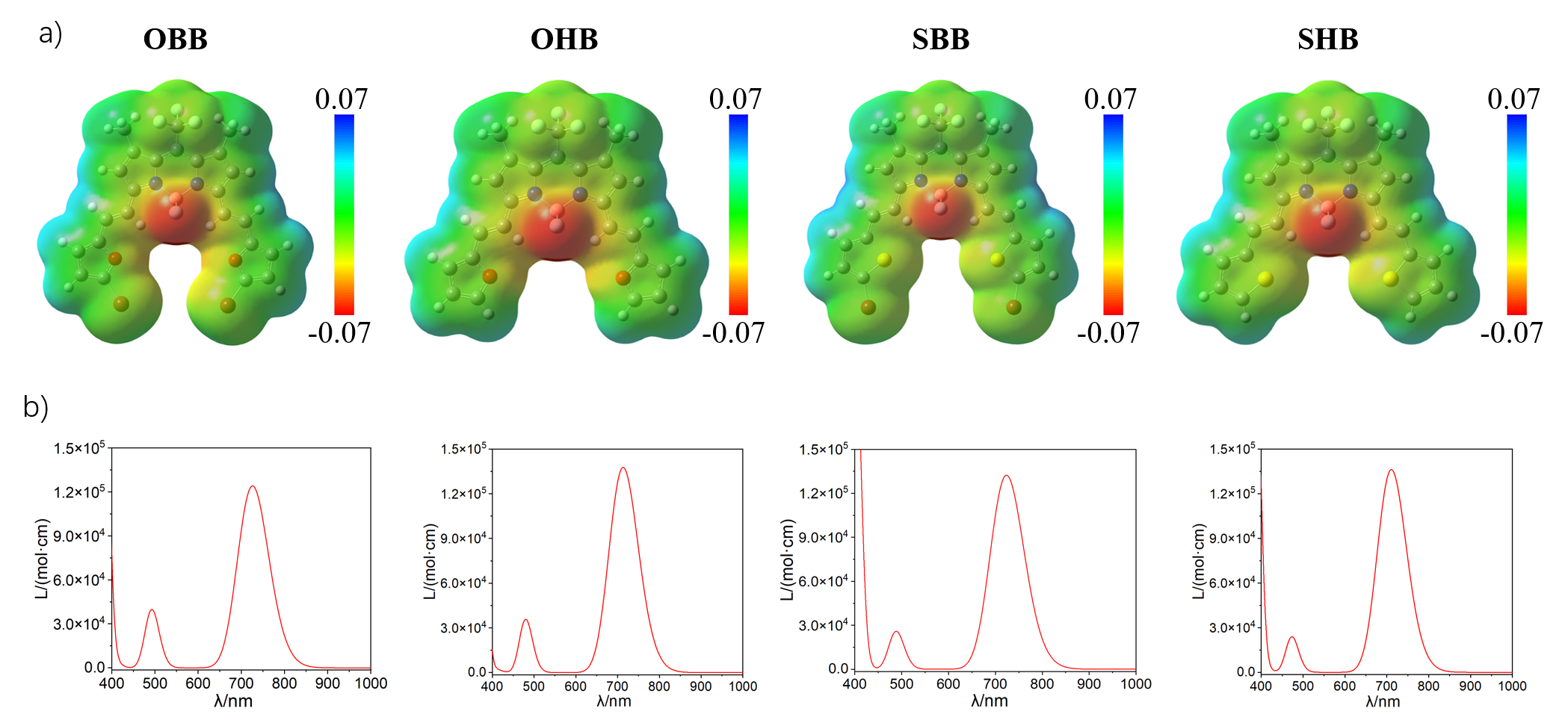


**FIGURE S11** a) ESP distribution diagram of **OBB**, **OHB**, **SBB** and **SHB**. b) Calculated absorption of **OBB**, **OHB**, **SBB** and **SHB**.


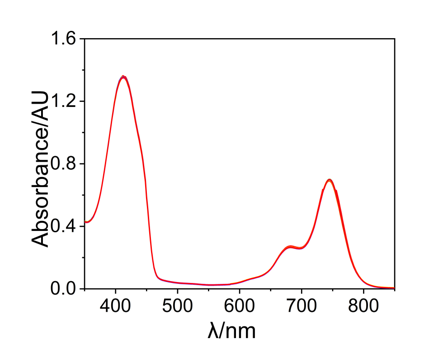


**FIGURE S12** Time-dependent photodegradation of DPBF by **OBB**.


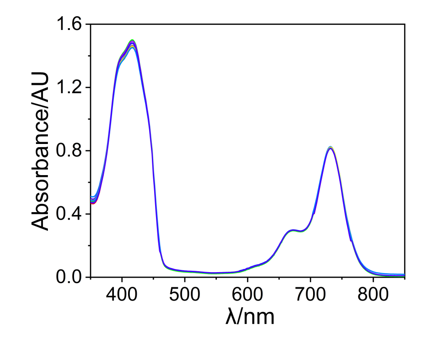


**FIGURE S13** Time-dependent photodegradation of DPBF by **OHB**.


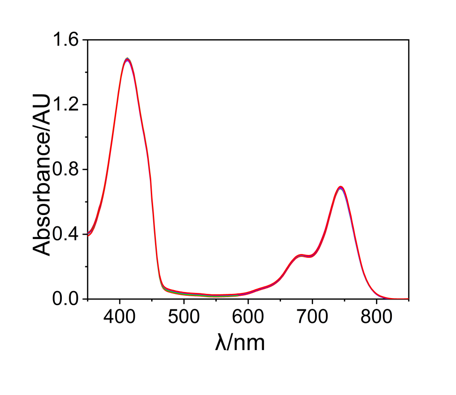


**FIGURE S14** Time-dependent photodegradation of DPBF by **SBB**.


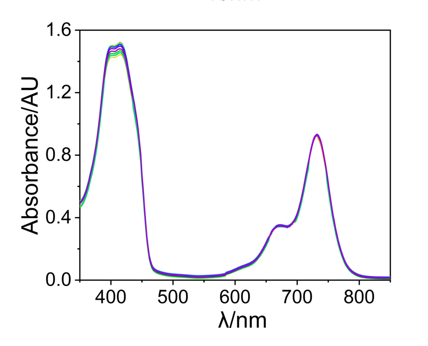


**FIGURE S15** Time-dependent photodegradation of DPBF by **SHB**.


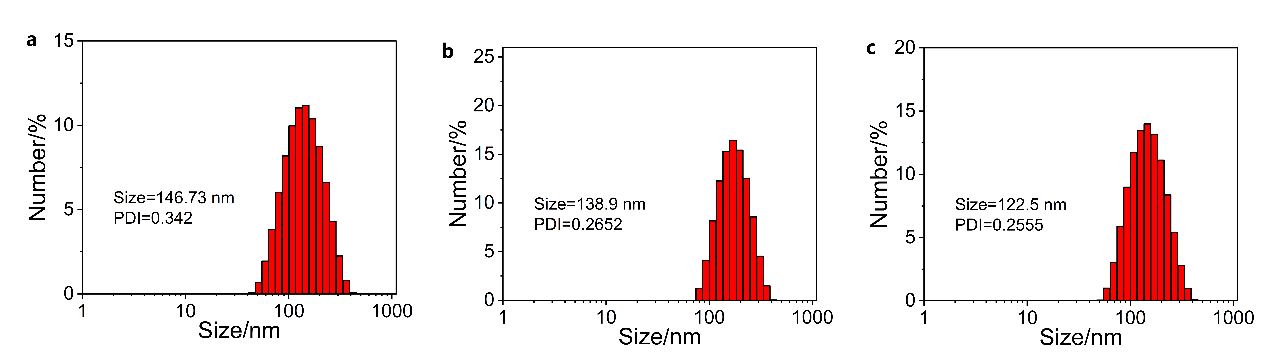


**FIGURE S16** DLS and of self-assembled nanoparticles a) **OHB** NPs, b) **SBB** NPs and c) **SHB** NPs.


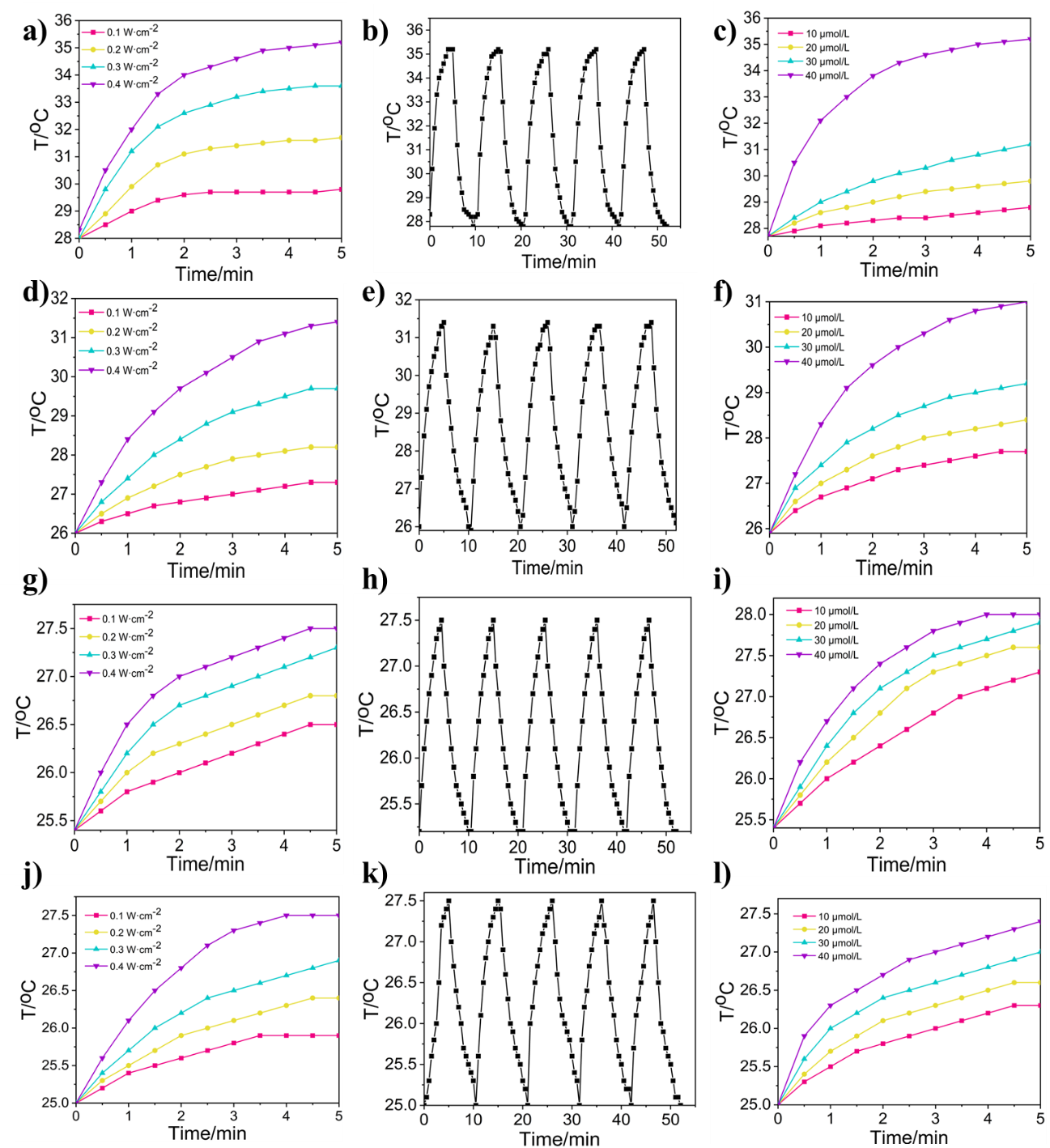


**FIGURE S17** light intensity-dependent photothermal effect, photothermal cycling stability, and concentration-dependent photothermal effect for a-c) **OBB** NPs, d-f) **OHB** NPs, g-i) **SBB** NPs and j-l) **SHB** NPs.


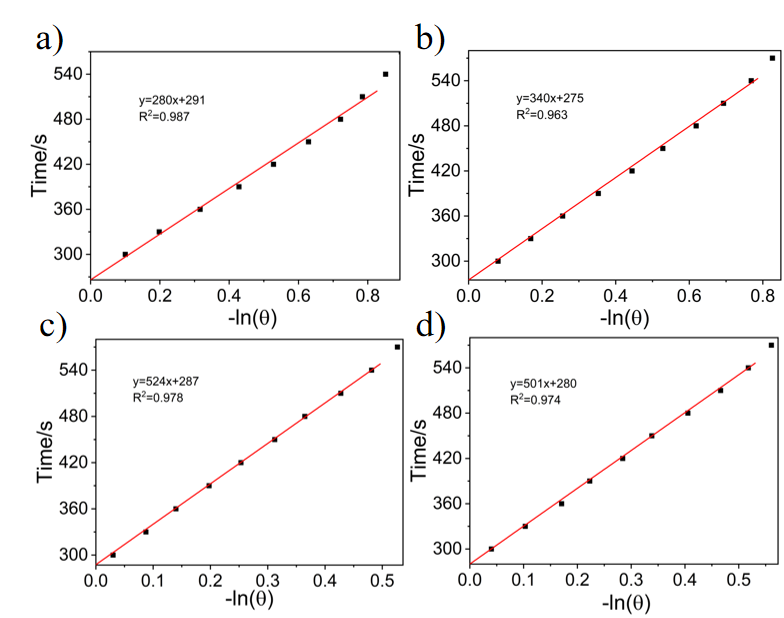


**FIGURE S18** Cooling time *versus* the negative natural logarithm of driving force temperature for a) **OBB** NPs (*η*= 49.7%), b) **OHB** NPs (*η*= 48%), c) **SBB** NPs (*η*= 46.6%) and d) **SHB** NPs (*η*= 46.1%).

**
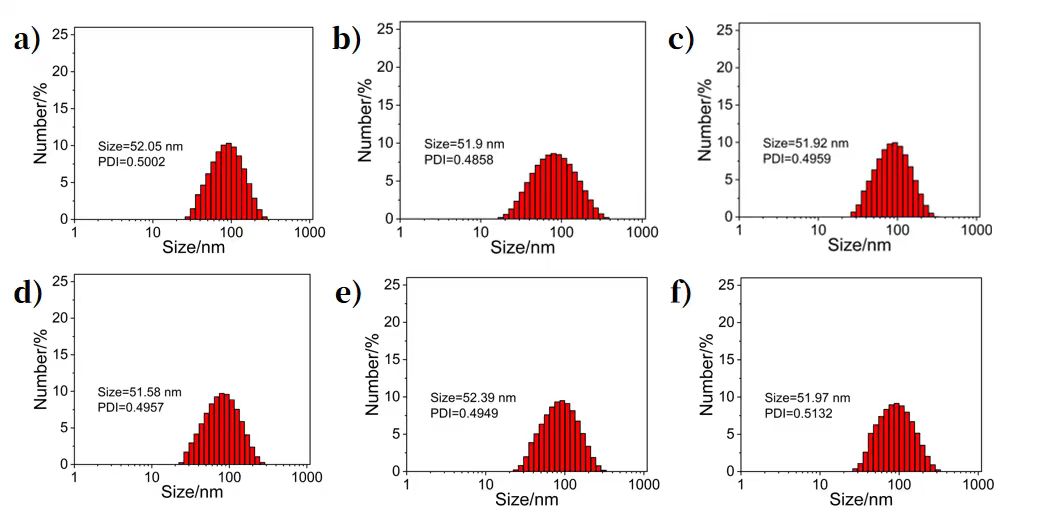
**

**FIGURE S19** Dynamic light scattering (DLS) measurements showing the hydrodynamic size distribution of **OBB** nanoparticles (**OBB** NPs) under physiological conditions. The size and polydispersity index (PDI) of **OBB** NPs were measured in cell culture medium at different time points: (a) 0 h; (b) 4 h; (c) 8 h; (d)12 h; (e) 16 h; and (f) 20 h.

**
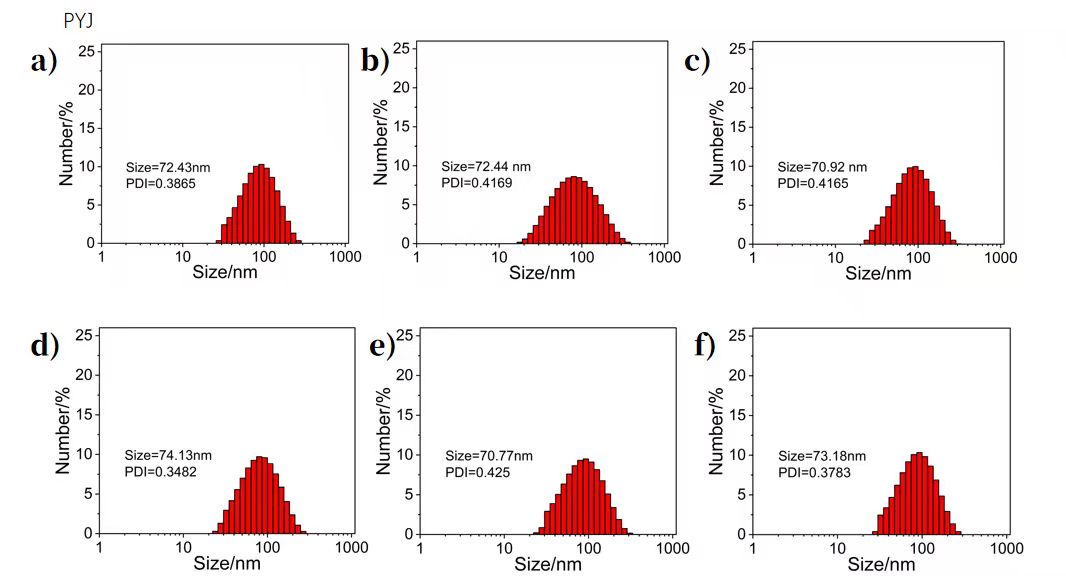
**

**FIGURE S20** Dynamic light scattering (DLS) measurements showing the hydrodynamic size distribution of **OBB** nanoparticles (**OBB** NPs) under physiological conditions. The size and polydispersity index (PDI) of **OBB** NPs were measured in PBS at different time points: (a) 0 h; (b) 4 h; (c) 8 h; (d)12 h; (e) 16 h; and (f) 20 h.

**
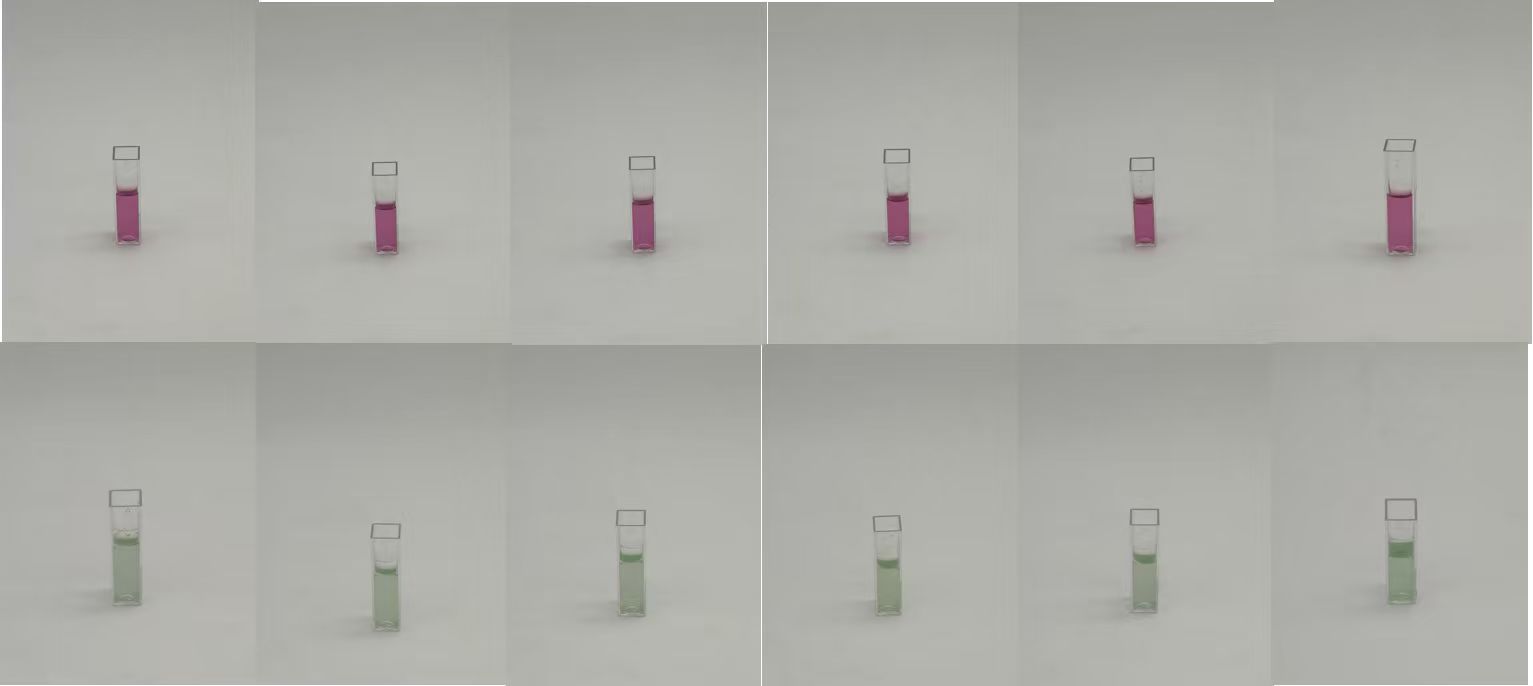
**

**FIGURE S21** Photographs of **OBB** nanoparticles (**OBB** NPs) dispersed in cell culture medium and PBS (pH 7.4) at different time points (0 to 20 h).


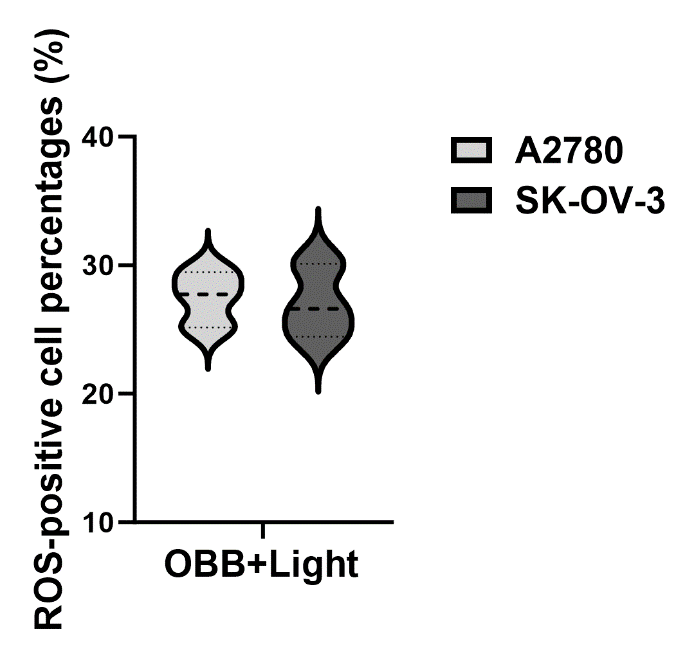


**FIGURE S22** ROS-positive cell percentages.

**4. Xray data**

**OB-BDP**

| **Table 1 Crystal data and structure refinement for OB-BDP.** | |
| --- | --- |
| Identification code | OB-BDP |
| Empirical formula | C_24_H_16_BBr_2_F_5_N_2_O_2_ |
| Formula weight | 630.02 |
| Temperature/K | 200.03(10) |
| Crystal system | triclinic |
| Space group | P-1 |
| a/Å | 8.29931(18) |
| b/Å | 11.28018(17) |
| c/Å | 25.7679(5) |
| α/° | 94.8673(14) |
| β/° | 97.7886(17) |
| γ/° | 90.7261(15) |
| Volume/Å^3^ | 2380.74(8) |
| Z | 4 |
| ρ_calc_g/cm^3^ | 1.758 |
| μ/mm^‑1^ | 4.915 |
| F(000) | 1240.0 |
| Crystal size/mm^3^ | 0.13 × 0.12 × 0.1 |
| Radiation | Cu Kα (λ = 1.54184) |
| 2Θ range for data collection/° | 7.868 to 145.716 |
| Index ranges | -10 ≤ h ≤ 10, -10 ≤ k ≤ 13, -31 ≤ l ≤ 31 |
| Reflections collected | 44599 |
| Independent reflections | 8924 [R_int_ = 0.0299, R_sigma_ = 0.0213] |
| Data/restraints/parameters | 8924/0/653 |
| Goodness-of-fit on F^2^ | 1.168 |
| Final R indexes [I>=2σ (I)] | R_1_ = 0.0790, wR_2_ = 0.1990 |
| Final R indexes [all data] | R_1_ = 0.0860, wR_2_ = 0.2029 |
| Largest diff. peak/hole / e Å^-3^ | 1.41/-0.63 |

Crystal structure determination of [OB-BDP]

Crystal Data for C_24_H_16_BBr_2_F_5_N_2_O_2_ (*M* =630.02 g/mol): triclinic, space group P-1 (no. 2), *a* = 8.29931(18) Å, *b* = 11.28018(17) Å, *c* = 25.7679(5) Å, *α* = 94.8673(14)°, *β* = 97.7886(17)°, *γ* = 90.7261(15)°, *V* = 2380.74(8) Å^3^, *Z* = 4, *T* = 200.03(10) K, μ(Cu Kα) = 4.915 mm^-1^, *Dcalc* = 1.758 g/cm^3^, 44599 reflections measured (7.868° ≤ 2Θ ≤ 145.716°), 8924 unique (*R*_int_ = 0.0299, R_sigma_ = 0.0213) which were used in all calculations. The final *R*_1_ was 0.0790 (I > 2σ(I)) and *wR*_2_ was 0.2029 (all data).

| Table 2 Fractional Atomic Coordinates (×10^4^) and Equivalent Isotropic Displacement Parameters (Å^2^×10^3^) for GSY_0B_2. U_eq_ is defined as 1/3 of the trace of the orthogonalised U_IJ_ tensor. | | | | |
| --- | --- | --- | --- | --- |
| Atom | *x* | *y* | *z* | U(eq) |
| Br_1_ | 1719.0(11) | -1724.4(8) | 4700.6(3) | 58.9(2) |
| Br_3_ | 6270.4(12) | 3626.5(9) | 4372.3(4) | 69.7(3) |
| F_1_ | -3412(5) | 1122(5) | 403.5(17) | 69.5(14) |
| F_2_ | -991(5) | 1703(4) | 319.1(15) | 54.2(10) |
| F_3_ | -1527(6) | -153(4) | 310.4(15) | 62.4(12) |
| F_4_ | 2172(5) | 376(3) | 1963.8(16) | 47.9(9) |
| F_5_ | 906(5) | 950(3) | 2666.2(14) | 46.7(9) |
| O_1_ | 360(5) | -2041(4) | 3676.5(16) | 38.3(10) |
| O_2_ | 4032(6) | 3968(4) | 3515.4(18) | 44.2(11) |
| N_1_ | -681(6) | -94(5) | 1900.8(19) | 35.4(11) |
| N_2_ | 277(6) | 1977(5) | 1886.3(19) | 34.6(11) |
| C_1_ | 444(9) | -2556(6) | 4136(3) | 44.7(16) |
| C_2_ | -396(9) | -3591(7) | 4088(3) | 49.0(17) |
| C_3_ | -1112(9) | -3750(6) | 3550(3) | 47.3(16) |
| C_4_ | -645(8) | -2804(6) | 3312(3) | 39.8(14) |
| C_5_ | -1016(8) | -2470(6) | 2792(2) | 42.6(15) |
| C_6_ | -547(8) | -1438(6) | 2613(2) | 35.9(13) |
| C_7_ | -1097(8) | -1132(6) | 2089(2) | 39.1(14) |
| C_8_ | -2234(9) | -1756(7) | 1702(3) | 49.1(17) |
| C_9_ | -2468(10) | -1139(7) | 1266(3) | 50.5(18) |
| C_10_ | -3667(12) | -1513(9) | 791(3) | 73(3) |
| C_11_ | -1458(8) | -75(6) | 1389(2) | 38.8(14) |
| C_12_ | -1320(8) | 971(6) | 1132(2) | 40.5(15) |
| C_13_ | -1826(9) | 905(7) | 542(3) | 48.8(18) |
| C_14_ | -643(7) | 2018(6) | 1397(2) | 34.7(13) |
| C_15_ | -789(8) | 3274(6) | 1300(3) | 39.7(14) |
| C_16_ | 49(8) | 3892(6) | 1725(3) | 40.8(15) |
| C_17_ | 751(8) | 3114(6) | 2084(2) | 36.9(13) |
| C_18_ | 1812(8) | 3394(6) | 2571(3) | 37.3(14) |
| C_19_ | 2036(8) | 4496(6) | 2805(3) | 41.5(15) |
| C_20_ | 3090(9) | 4837(6) | 3281(3) | 43.3(15) |
| C_21_ | 3364(9) | 5884(6) | 3578(3) | 48.3(17) |
| C_22_ | 4517(9) | 5706(7) | 4016(3) | 53.3(18) |
| C_23_ | 4882(9) | 4543(7) | 3957(3) | 47.2(16) |
| C_24_ | -1838(9) | 3810(7) | 861(3) | 53.9(19) |
| B_1_ | 718(9) | 791(7) | 2123(3) | 36.4(15) |
| Br_2_ | 11282.3(12) | 3328.0(9) | 4340.9(4) | 68.0(3) |
| Br_4_ | 6814.4(11) | 8679.6(8) | 4662.9(3) | 59.2(2) |
| F_6_ | 1576(5) | 3962(5) | 387.3(16) | 68.8(14) |
| F_7_ | 3421(6) | 5258(4) | 291.9(15) | 63.0(12) |
| F_8_ | 4003(5) | 3438(4) | 309.9(15) | 54.9(11) |
| F_9_ | 5836(5) | 5143(3) | 2657.1(13) | 44.1(9) |
| F_10_ | 7133(4) | 5438(3) | 1960.0(16) | 46.9(9) |
| O_3_ | 9020(6) | 2553(4) | 3501.1(18) | 43.1(11) |
| O_4_ | 5394(6) | 8544(4) | 3645.6(17) | 41.3(10) |
| N_3_ | 5238(6) | 3783(5) | 1876.2(19) | 34.5(11) |
| N_4_ | 4284(6) | 5849(5) | 1885.5(18) | 35.8(11) |
| C_01O_ | 3152(9) | 4278(7) | 530(3) | 48.9(17) |
| C_25_ | 3177(9) | 1499(7) | 849(3) | 52.7(18) |
| C_26_ | 4209(8) | 2240(6) | 1284(2) | 40.1(15) |
| C_27_ | 5057(8) | 1796(6) | 1714(3) | 41.9(15) |
| C_28_ | 5736(8) | 2749(6) | 2074(2) | 36.7(14) |
| C_29_ | 6783(8) | 2690(6) | 2558(3) | 40.6(15) |
| C_30_ | 7056(9) | 1679(6) | 2792(3) | 43.8(15) |
| C_31_ | 8119(8) | 1572(6) | 3271(3) | 41.9(15) |
| C_32_ | 8478(10) | 631(6) | 3565(3) | 50.5(18) |
| C_33_ | 9667(10) | 1036(7) | 3988(3) | 52.7(19) |
| C_34_ | 9919(9) | 2175(7) | 3931(3) | 47.7(17) |
| C_35_ | 4318(8) | 3528(6) | 1386(2) | 36.8(14) |
| C_36_ | 3651(8) | 4461(6) | 1118(2) | 38.5(14) |
| C_37_ | 3499(8) | 5601(6) | 1375(2) | 40.8(15) |
| C_38_ | 2500(9) | 6613(7) | 1247(3) | 48.7(17) |
| C_39_ | 1296(12) | 6760(9) | 767(3) | 73(3) |
| C_40_ | 2720(9) | 7396(6) | 1679(3) | 45.5(16) |
| C_41_ | 3852(8) | 6945(6) | 2073(2) | 38.2(14) |
| C_42_ | 4445(8) | 7485(6) | 2591(2) | 42.0(15) |
| C_43_ | 4001(8) | 8598(6) | 2760(2) | 40.1(14) |
| C_44_ | 4417(8) | 9152(6) | 3281(3) | 39.7(14) |
| C_45_ | 4025(9) | 10195(6) | 3521(3) | 49.0(17) |
| C_46_ | 4745(10) | 10277(7) | 4040(3) | 52.7(18) |
| C_47_ | 5518(9) | 9262(6) | 4101(3) | 44.4(16) |
| B_2_ | 5681(9) | 5062(7) | 2119(3) | 35.0(15) |

| Table 3 Anisotropic Displacement Parameters (Å^2^×10^3^) for OB-BDP. The Anisotropic displacement factor exponent takes the form: -2π^2^[h^2^a*^2^U_11_+2hka*b*U_12_+…]. | | | | | | |
| --- | --- | --- | --- | --- | --- | --- |
| Atom | U_11_ | U_22_ | U_33_ | U_23_ | U_13_ | U_12_ |
| Br_1_ | 72.5(6) | 58.6(5) | 39.4(4) | 3.7(3) | -13.5(4) | -1.9(4) |
| Br_3_ | 66.3(6) | 69.6(6) | 67.4(6) | 8.3(4) | -13.9(4) | 6.4(4) |
| F_1_ | 44(2) | 121(4) | 42(2) | 16(2) | -7.9(19) | 5(2) |
| F_2_ | 59(3) | 72(3) | 33(2) | 15.3(19) | 5.7(18) | 3(2) |
| F_3_ | 85(3) | 67(3) | 33(2) | -2.6(19) | 5(2) | -4(2) |
| F_4_ | 41(2) | 45(2) | 62(2) | 18.2(19) | 9.6(18) | 7.5(17) |
| F_5_ | 59(2) | 49(2) | 29.0(18) | 8.1(16) | -7.6(16) | -15.5(18) |
| O_1_ | 50(3) | 31(2) | 33(2) | 6.2(17) | -4.0(19) | -0.3(19) |
| O_2_ | 49(3) | 39(3) | 41(2) | 0(2) | -1(2) | 0(2) |
| N_1_ | 39(3) | 41(3) | 25(2) | 3(2) | 3(2) | -3(2) |
| N_2_ | 39(3) | 37(3) | 28(2) | 6(2) | 2(2) | 3(2) |
| C_1_ | 52(4) | 46(4) | 35(3) | 0(3) | 3(3) | 6(3) |
| C_2_ | 59(4) | 50(4) | 39(4) | 9(3) | 4(3) | -6(3) |
| C_3_ | 52(4) | 41(4) | 45(4) | 3(3) | -3(3) | -9(3) |
| C_4_ | 46(4) | 35(3) | 36(3) | 2(3) | -1(3) | -3(3) |
| C_5_ | 50(4) | 41(4) | 32(3) | -4(3) | -2(3) | -6(3) |
| C_6_ | 44(3) | 35(3) | 27(3) | 0(2) | 1(2) | -5(3) |
| C_7_ | 44(4) | 42(4) | 30(3) | 1(3) | 2(3) | -2(3) |
| C_8_ | 60(4) | 48(4) | 34(3) | -3(3) | -6(3) | -15(3) |
| C_9_ | 57(4) | 55(4) | 36(4) | 1(3) | -3(3) | -10(3) |
| C_10_ | 91(7) | 81(6) | 40(4) | 3(4) | -18(4) | -29(5) |
| C_11_ | 41(3) | 47(4) | 26(3) | 3(3) | 0(3) | -2(3) |
| C_12_ | 31(3) | 59(4) | 31(3) | 4(3) | 1(2) | 4(3) |
| C_13_ | 46(4) | 74(5) | 26(3) | 10(3) | 1(3) | -3(3) |
| C_14_ | 29(3) | 44(4) | 33(3) | 11(3) | 6(2) | 6(3) |
| C_15_ | 35(3) | 50(4) | 36(3) | 12(3) | 9(3) | 6(3) |
| C_16_ | 42(4) | 37(4) | 44(4) | 8(3) | 8(3) | 6(3) |
| C_17_ | 39(3) | 40(4) | 33(3) | 5(3) | 7(3) | 4(3) |
| C_18_ | 42(3) | 30(3) | 41(3) | 5(3) | 6(3) | 2(3) |
| C_19_ | 47(4) | 39(4) | 39(3) | 5(3) | 5(3) | 2(3) |
| C_20_ | 49(4) | 39(4) | 43(4) | 5(3) | 7(3) | 2(3) |
| C_21_ | 57(4) | 33(4) | 54(4) | 2(3) | 8(3) | -2(3) |
| C_22_ | 56(4) | 46(4) | 55(4) | -8(3) | 5(4) | -9(3) |
| C_23_ | 49(4) | 48(4) | 42(4) | 3(3) | 1(3) | 0(3) |
| C_24_ | 54(4) | 60(5) | 49(4) | 20(4) | 4(3) | 18(4) |
| B_1_ | 34(4) | 43(4) | 32(3) | 1(3) | 3(3) | -5(3) |
| Br_2_ | 63.1(5) | 79.2(6) | 59.0(5) | 19.7(4) | -9.4(4) | -7.9(4) |
| Br_4_ | 68.6(5) | 65.0(5) | 37.4(4) | -5.9(3) | -9.6(3) | 0.4(4) |
| F_6_ | 38(2) | 121(4) | 40(2) | -12(2) | -9.7(18) | -6(2) |
| F_7_ | 84(3) | 73(3) | 32(2) | 11(2) | 2(2) | 7(2) |
| F_8_ | 61(3) | 68(3) | 32(2) | -12.5(18) | 4.4(18) | 5(2) |
| F_9_ | 59(2) | 43(2) | 26.7(17) | -2.0(15) | -6.7(16) | 12.3(17) |
| F_10_ | 39(2) | 39(2) | 61(2) | -5.6(18) | 6.1(18) | -3.0(16) |
| O_3_ | 47(3) | 41(3) | 42(2) | 13(2) | 1(2) | 3(2) |
| O_4_ | 53(3) | 36(2) | 33(2) | -4.0(18) | 2.1(19) | 2(2) |
| N_3_ | 38(3) | 34(3) | 29(2) | 0(2) | 0(2) | 0(2) |
| N_4_ | 41(3) | 43(3) | 22(2) | 1(2) | -1(2) | 3(2) |
| C_01O_ | 43(4) | 68(5) | 34(3) | -1(3) | 1(3) | -2(3) |
| C_25_ | 49(4) | 54(4) | 51(4) | -11(3) | 2(3) | -6(3) |
| C_26_ | 32(3) | 52(4) | 35(3) | -8(3) | 6(3) | -6(3) |
| C_27_ | 42(4) | 34(3) | 49(4) | -3(3) | 8(3) | -8(3) |
| C_28_ | 37(3) | 38(4) | 37(3) | 3(3) | 10(3) | 0(3) |
| C_29_ | 42(4) | 41(4) | 38(3) | 3(3) | 5(3) | 3(3) |
| C_30_ | 54(4) | 37(4) | 42(4) | 3(3) | 11(3) | 3(3) |
| C_31_ | 47(4) | 39(4) | 43(4) | 6(3) | 14(3) | 9(3) |
| C_32_ | 69(5) | 35(4) | 52(4) | 16(3) | 17(4) | 12(3) |
| C_33_ | 62(5) | 54(5) | 46(4) | 19(3) | 11(3) | 20(4) |
| C_34_ | 45(4) | 51(4) | 48(4) | 20(3) | 1(3) | 7(3) |
| C_35_ | 36(3) | 47(4) | 26(3) | -6(3) | 6(2) | -4(3) |
| C_36_ | 38(3) | 50(4) | 27(3) | 2(3) | 2(2) | -1(3) |
| C_37_ | 43(4) | 51(4) | 27(3) | 5(3) | -1(3) | 5(3) |
| C_38_ | 56(4) | 59(5) | 30(3) | 5(3) | -1(3) | 13(3) |
| C_39_ | 85(6) | 83(7) | 43(4) | 6(4) | -19(4) | 32(5) |
| C_40_ | 53(4) | 45(4) | 35(3) | 3(3) | -6(3) | 13(3) |
| C_41_ | 45(4) | 38(4) | 31(3) | 5(3) | 1(3) | 6(3) |
| C_42_ | 46(4) | 46(4) | 32(3) | 4(3) | -2(3) | 4(3) |
| C_43_ | 46(4) | 39(4) | 33(3) | 1(3) | -2(3) | 8(3) |
| C_44_ | 44(4) | 36(4) | 36(3) | 3(3) | -1(3) | 4(3) |
| C_45_ | 53(4) | 41(4) | 51(4) | 1(3) | 3(3) | 9(3) |
| C_46_ | 65(5) | 44(4) | 48(4) | -8(3) | 10(4) | 5(3) |
| C_47_ | 58(4) | 39(4) | 35(3) | -2(3) | 6(3) | -4(3) |
| B_2_ | 37(4) | 39(4) | 27(3) | -2(3) | -3(3) | 4(3) |

| Table 4 Bond Lengths for OB-BDP. | | | | | | |
| --- | --- | --- | --- | --- | --- | --- |
| Atom | Atom | Length/Å |  | Atom | Atom | Length/Å |
| Br_1_ | C_1_ | 1.856(7) |  | Br_2_ | C_34_ | 1.869(8) |
| Br_3_ | C_23_ | 1.852(7) |  | Br_4_ | C_47_ | 1.856(7) |
| F_1_ | C_13_ | 1.347(8) |  | F_6_ | C_01O_ | 1.345(8) |
| F_2_ | C_13_ | 1.342(8) |  | F_7_ | C_01O_ | 1.339(9) |
| F_3_ | C_13_ | 1.330(9) |  | F_8_ | C_01O_ | 1.323(9) |
| F_4_ | B_1_ | 1.400(8) |  | F_9_ | B_2_ | 1.371(7) |
| F_5_ | B_1_ | 1.384(8) |  | F_10_ | B_2_ | 1.397(8) |
| O_1_ | C_1_ | 1.356(8) |  | O_3_ | C_31_ | 1.373(8) |
| O_1_ | C_4_ | 1.397(7) |  | O_3_ | C_34_ | 1.353(8) |
| O_2_ | C_20_ | 1.389(8) |  | O_4_ | C_44_ | 1.387(8) |
| O_2_ | C_23_ | 1.363(8) |  | O_4_ | C_47_ | 1.359(8) |
| N_1_ | C_7_ | 1.363(8) |  | N_3_ | C_28_ | 1.359(8) |
| N_1_ | C_11_ | 1.390(8) |  | N_3_ | C_35_ | 1.390(8) |
| N_1_ | B_1_ | 1.538(8) |  | N_3_ | B_2_ | 1.542(9) |
| N_2_ | C_14_ | 1.388(8) |  | N_4_ | C_37_ | 1.392(8) |
| N_2_ | C_17_ | 1.372(8) |  | N_4_ | C_41_ | 1.360(8) |
| N_2_ | B_1_ | 1.544(9) |  | N_4_ | B_2_ | 1.556(9) |
| C_1_ | C_2_ | 1.340(10) |  | C_01O_ | C_36_ | 1.512(9) |
| C_2_ | C_3_ | 1.428(10) |  | C_25_ | C_26_ | 1.501(9) |
| C_3_ | C_4_ | 1.353(10) |  | C_26_ | C_27_ | 1.365(10) |
| C_4_ | C_5_ | 1.417(9) |  | C_26_ | C_35_ | 1.454(9) |
| C_5_ | C_6_ | 1.359(9) |  | C_27_ | C_28_ | 1.417(9) |
| C_6_ | C_7_ | 1.438(8) |  | C_28_ | C_29_ | 1.428(9) |
| C_7_ | C_8_ | 1.410(9) |  | C_29_ | C_30_ | 1.342(10) |
| C_8_ | C_9_ | 1.363(10) |  | C_30_ | C_31_ | 1.432(10) |
| C_9_ | C_10_ | 1.497(10) |  | C_31_ | C_32_ | 1.368(10) |
| C_9_ | C_11_ | 1.443(10) |  | C_32_ | C_33_ | 1.409(11) |
| C_11_ | C_12_ | 1.411(10) |  | C_33_ | C_34_ | 1.323(11) |
| C_12_ | C_13_ | 1.517(9) |  | C_35_ | C_36_ | 1.388(9) |
| C_12_ | C_14_ | 1.385(9) |  | C_36_ | C_37_ | 1.410(9) |
| C_14_ | C_15_ | 1.463(9) |  | C_37_ | C_38_ | 1.453(10) |
| C_15_ | C_16_ | 1.348(10) |  | C_38_ | C_39_ | 1.504(9) |
| C_15_ | C_24_ | 1.506(9) |  | C_38_ | C_40_ | 1.351(10) |
| C_16_ | C_17_ | 1.404(9) |  | C_40_ | C_41_ | 1.420(9) |
| C_17_ | C_18_ | 1.441(9) |  | C_41_ | C_42_ | 1.438(9) |
| C_18_ | C_19_ | 1.334(9) |  | C_42_ | C_43_ | 1.365(9) |
| C_19_ | C_20_ | 1.426(10) |  | C_43_ | C_44_ | 1.429(9) |
| C_20_ | C_21_ | 1.351(10) |  | C_44_ | C_45_ | 1.347(9) |
| C_21_ | C_22_ | 1.408(11) |  | C_45_ | C_46_ | 1.384(10) |
| C_22_ | C_23_ | 1.350(11) |  | C_46_ | C_47_ | 1.330(11) |

| Table 5 Bond Angles for OB-BDP. | | | | | | | | |
| --- | --- | --- | --- | --- | --- | --- | --- | --- |
| Atom | Atom | Atom | Angle/˚ |  | Atom | Atom | Atom | Angle/˚ |
| C_1_ | O_1_ | C_4_ | 105.1(5) |  | C_34_ | O_3_ | C_31_ | 105.0(5) |
| C_23_ | O_2_ | C_20_ | 105.0(5) |  | C_47_ | O_4_ | C_44_ | 104.9(5) |
| C_7_ | N_1_ | C_11_ | 108.6(5) |  | C_28_ | N_3_ | C_35_ | 109.2(5) |
| C_7_ | N_1_ | B_1_ | 128.5(5) |  | C_28_ | N_3_ | B_2_ | 127.5(5) |
| C_11_ | N_1_ | B_1_ | 121.1(5) |  | C_35_ | N_3_ | B_2_ | 123.2(5) |
| C_14_ | N_2_ | B_1_ | 122.3(5) |  | C_37_ | N_4_ | B_2_ | 121.4(5) |
| C_17_ | N_2_ | C_14_ | 108.9(5) |  | C_41_ | N_4_ | C_37_ | 108.8(5) |
| C_17_ | N_2_ | B_1_ | 128.8(5) |  | C_41_ | N_4_ | B_2_ | 128.6(5) |
| O_1_ | C_1_ | Br_1_ | 114.7(5) |  | F_6_ | C_01O_ | C_36_ | 113.9(6) |
| C_2_ | C_1_ | Br_1_ | 132.5(6) |  | F_7_ | C_01O_ | F_6_ | 106.9(6) |
| C_2_ | C_1_ | O_1_ | 112.8(6) |  | F_7_ | C_01O_ | C_36_ | 112.0(6) |
| C_1_ | C_2_ | C_3_ | 105.2(6) |  | F_8_ | C_01O_ | F_6_ | 106.4(6) |
| C_4_ | C_3_ | C_2_ | 107.4(6) |  | F_8_ | C_01O_ | F_7_ | 105.4(6) |
| O_1_ | C_4_ | C_5_ | 118.2(6) |  | F_8_ | C_01O_ | C_36_ | 111.7(6) |
| C_3_ | C_4_ | O_1_ | 109.4(6) |  | C_27_ | C_26_ | C_25_ | 124.4(7) |
| C_3_ | C_4_ | C_5_ | 132.4(6) |  | C_27_ | C_26_ | C_35_ | 106.1(5) |
| C_6_ | C_5_ | C_4_ | 126.2(6) |  | C_35_ | C_26_ | C_25_ | 128.9(6) |
| C_5_ | C_6_ | C_7_ | 122.2(6) |  | C_26_ | C_27_ | C_28_ | 109.4(6) |
| N_1_ | C_7_ | C_6_ | 123.8(6) |  | N_3_ | C_28_ | C_27_ | 107.9(6) |
| N_1_ | C_7_ | C_8_ | 108.0(6) |  | N_3_ | C_28_ | C_29_ | 123.9(6) |
| C_8_ | C_7_ | C_6_ | 128.0(6) |  | C_27_ | C_28_ | C_29_ | 128.2(6) |
| C_9_ | C_8_ | C_7_ | 109.5(6) |  | C_30_ | C_29_ | C_28_ | 123.3(7) |
| C_8_ | C_9_ | C_10_ | 123.7(7) |  | C_29_ | C_30_ | C_31_ | 125.3(7) |
| C_8_ | C_9_ | C_11_ | 106.1(6) |  | O_3_ | C_31_ | C_30_ | 118.6(6) |
| C_11_ | C_9_ | C_10_ | 130.0(7) |  | C_32_ | C_31_ | O_3_ | 109.2(6) |
| N_1_ | C_11_ | C_9_ | 107.7(6) |  | C_32_ | C_31_ | C_30_ | 132.2(7) |
| N_1_ | C_11_ | C_12_ | 118.3(6) |  | C_31_ | C_32_ | C_33_ | 107.2(7) |
| C_12_ | C_11_ | C_9_ | 133.3(6) |  | C_34_ | C_33_ | C_32_ | 105.3(6) |
| C_11_ | C_12_ | C_13_ | 118.1(6) |  | O_3_ | C_34_ | Br_2_ | 115.6(5) |
| C_14_ | C_12_ | C_11_ | 122.0(6) |  | C_33_ | C_34_ | Br_2_ | 131.1(6) |
| C_14_ | C_12_ | C_13_ | 119.8(6) |  | C_33_ | C_34_ | O_3_ | 113.3(7) |
| F_1_ | C_13_ | C_12_ | 113.5(6) |  | N_3_ | C_35_ | C_26_ | 107.3(6) |
| F_2_ | C_13_ | F_1_ | 106.2(6) |  | C_36_ | C_35_ | N_3_ | 118.8(6) |
| F_2_ | C_13_ | C_12_ | 111.4(6) |  | C_36_ | C_35_ | C_26_ | 133.8(6) |
| F_3_ | C_13_ | F_1_ | 107.7(6) |  | C_35_ | C_36_ | C_01O_ | 119.5(6) |
| F_3_ | C_13_ | F_2_ | 105.9(6) |  | C_35_ | C_36_ | C_37_ | 121.8(6) |
| F_3_ | C_13_ | C_12_ | 111.7(6) |  | C_37_ | C_36_ | C_01O_ | 118.6(6) |
| N_2_ | C_14_ | C_15_ | 107.0(6) |  | N_4_ | C_37_ | C_36_ | 118.7(6) |
| C_12_ | C_14_ | N_2_ | 119.0(6) |  | N_4_ | C_37_ | C_38_ | 107.4(6) |
| C_12_ | C_14_ | C_15_ | 133.7(6) |  | C_36_ | C_37_ | C_38_ | 133.5(6) |
| C_14_ | C_15_ | C_24_ | 128.1(6) |  | C_37_ | C_38_ | C_39_ | 129.2(7) |
| C_16_ | C_15_ | C_14_ | 106.0(6) |  | C_40_ | C_38_ | C_37_ | 106.2(6) |
| C_16_ | C_15_ | C_24_ | 125.4(7) |  | C_40_ | C_38_ | C_39_ | 124.2(7) |
| C_15_ | C_16_ | C_17_ | 110.5(6) |  | C_38_ | C_40_ | C_41_ | 109.7(6) |
| N_2_ | C_17_ | C_16_ | 107.6(6) |  | N_4_ | C_41_ | C_40_ | 107.8(5) |
| N_2_ | C_17_ | C_18_ | 123.7(6) |  | N_4_ | C_41_ | C_42_ | 123.7(6) |
| C_16_ | C_17_ | C_18_ | 128.7(6) |  | C_40_ | C_41_ | C_42_ | 128.5(6) |
| C_19_ | C_18_ | C_17_ | 123.0(6) |  | C_43_ | C_42_ | C_41_ | 121.7(6) |
| C_18_ | C_19_ | C_20_ | 125.8(6) |  | C_42_ | C_43_ | C_44_ | 125.1(6) |
| O_2_ | C_20_ | C_19_ | 118.1(6) |  | O_4_ | C_44_ | C_43_ | 118.5(6) |
| C_21_ | C_20_ | O_2_ | 109.3(6) |  | C_45_ | C_44_ | O_4_ | 108.4(6) |
| C_21_ | C_20_ | C_19_ | 132.5(7) |  | C_45_ | C_44_ | C_43_ | 133.1(6) |
| C_20_ | C_21_ | C_22_ | 108.3(7) |  | C_44_ | C_45_ | C_46_ | 108.9(7) |
| C_23_ | C_22_ | C_21_ | 105.0(6) |  | C_47_ | C_46_ | C_45_ | 105.6(6) |
| O_2_ | C_23_ | Br_3_ | 116.4(5) |  | O_4_ | C_47_ | Br_4_ | 114.4(5) |
| C_22_ | C_23_ | Br_3_ | 131.3(6) |  | C_46_ | C_47_ | Br_4_ | 133.2(6) |
| C_22_ | C_23_ | O_2_ | 112.3(6) |  | C_46_ | C_47_ | O_4_ | 112.2(6) |
| F_4_ | B_1_ | N_1_ | 109.5(5) |  | F_9_ | B_2_ | F_10_ | 109.6(5) |
| F_4_ | B_1_ | N_2_ | 110.0(5) |  | F_9_ | B_2_ | N_3_ | 111.9(5) |
| F_5_ | B_1_ | F_4_ | 109.0(5) |  | F_9_ | B_2_ | N_4_ | 111.3(5) |
| F_5_ | B_1_ | N_1_ | 112.0(5) |  | F_10_ | B_2_ | N_3_ | 109.6(5) |
| F_5_ | B_1_ | N_2_ | 110.3(5) |  | F_10_ | B_2_ | N_4_ | 108.6(5) |
| N_1_ | B_1_ | N_2_ | 106.1(5) |  | N_3_ | B_2_ | N_4_ | 105.7(5) |

| Table 6 Torsion Angles for OB-BDP. | | | | | | | | | | |
| --- | --- | --- | --- | --- | --- | --- | --- | --- | --- | --- |
| A | B | C | D | Angle/˚ |  | A | B | C | D | Angle/˚ |
| Br_1_ | C_1_ | C_2_ | C_3_ | -179.5(6) |  | F_6_ | C_01O_ | C_36_ | C_35_ | 94.1(8) |
| O_1_ | C_1_ | C_2_ | C_3_ | -1.1(9) |  | F_6_ | C_01O_ | C_36_ | C_37_ | -89.1(8) |
| O_1_ | C_4_ | C_5_ | C_6_ | -3.2(11) |  | F_7_ | C_01O_ | C_36_ | C_35_ | -144.5(6) |
| O_2_ | C_20_ | C_21_ | C_22_ | 0.4(8) |  | F_7_ | C_01O_ | C_36_ | C_37_ | 32.3(9) |
| N_1_ | C_7_ | C_8_ | C_9_ | 2.8(9) |  | F_8_ | C_01O_ | C_36_ | C_35_ | -26.5(9) |
| N_1_ | C_11_ | C_12_ | C_13_ | 166.3(6) |  | F_8_ | C_01O_ | C_36_ | C_37_ | 150.3(6) |
| N_1_ | C_11_ | C_12_ | C_14_ | -9.4(10) |  | O_3_ | C_31_ | C_32_ | C_33_ | -1.0(8) |
| N_2_ | C_14_ | C_15_ | C_16_ | 0.7(7) |  | O_4_ | C_44_ | C_45_ | C_46_ | 0.3(9) |
| N_2_ | C_14_ | C_15_ | C_24_ | -171.4(6) |  | N_3_ | C_28_ | C_29_ | C_30_ | 168.8(6) |
| N_2_ | C_17_ | C_18_ | C_19_ | -167.8(6) |  | N_3_ | C_35_ | C_36_ | C_01O_ | 159.4(6) |
| C_1_ | O_1_ | C_4_ | C_3_ | -1.0(7) |  | N_3_ | C_35_ | C_36_ | C_37_ | -17.3(9) |
| C_1_ | O_1_ | C_4_ | C_5_ | 177.5(6) |  | N_4_ | C_37_ | C_38_ | C_39_ | -173.9(8) |
| C_1_ | C_2_ | C_3_ | C_4_ | 0.4(9) |  | N_4_ | C_37_ | C_38_ | C_40_ | -0.4(8) |
| C_2_ | C_3_ | C_4_ | O_1_ | 0.4(8) |  | N_4_ | C_41_ | C_42_ | C_43_ | 178.8(6) |
| C_2_ | C_3_ | C_4_ | C_5_ | -177.8(8) |  | C_01O_ | C_36_ | C_37_ | N_4_ | -165.6(6) |
| C_3_ | C_4_ | C_5_ | C_6_ | 174.9(8) |  | C_01O_ | C_36_ | C_37_ | C_38_ | 23.2(12) |
| C_4_ | O_1_ | C_1_ | Br_1_ | -180.0(4) |  | C_25_ | C_26_ | C_27_ | C_28_ | -173.1(6) |
| C_4_ | O_1_ | C_1_ | C_2_ | 1.3(8) |  | C_25_ | C_26_ | C_35_ | N_3_ | 170.9(6) |
| C_4_ | C_5_ | C_6_ | C_7_ | -174.7(7) |  | C_25_ | C_26_ | C_35_ | C_36_ | -5.2(12) |
| C_5_ | C_6_ | C_7_ | N_1_ | 178.4(6) |  | C_26_ | C_27_ | C_28_ | N_3_ | 2.9(7) |
| C_5_ | C_6_ | C_7_ | C_8_ | 4.7(11) |  | C_26_ | C_27_ | C_28_ | C_29_ | -176.2(6) |
| C_6_ | C_7_ | C_8_ | C_9_ | 177.4(7) |  | C_26_ | C_35_ | C_36_ | C_01O_ | -24.9(11) |
| C_7_ | N_1_ | C_11_ | C_9_ | 2.9(8) |  | C_26_ | C_35_ | C_36_ | C_37_ | 158.4(7) |
| C_7_ | N_1_ | C_11_ | C_12_ | 174.2(6) |  | C_27_ | C_26_ | C_35_ | N_3_ | 0.1(7) |
| C_7_ | N_1_ | B_1_ | F_4_ | 80.7(8) |  | C_27_ | C_26_ | C_35_ | C_36_ | -176.0(7) |
| C_7_ | N_1_ | B_1_ | F_5_ | -40.2(9) |  | C_27_ | C_28_ | C_29_ | C_30_ | -12.3(11) |
| C_7_ | N_1_ | B_1_ | N_2_ | -160.6(6) |  | C_28_ | N_3_ | C_35_ | C_26_ | 1.7(7) |
| C_7_ | C_8_ | C_9_ | C_10_ | -176.5(8) |  | C_28_ | N_3_ | C_35_ | C_36_ | 178.5(6) |
| C_7_ | C_8_ | C_9_ | C_11_ | -1.0(9) |  | C_28_ | N_3_ | B_2_ | F_9_ | -34.9(9) |
| C_8_ | C_9_ | C_11_ | N_1_ | -1.1(8) |  | C_28_ | N_3_ | B_2_ | F_10_ | 86.9(7) |
| C_8_ | C_9_ | C_11_ | C_12_ | -170.6(8) |  | C_28_ | N_3_ | B_2_ | N_4_ | -156.2(6) |
| C_9_ | C_11_ | C_12_ | C_13_ | -25.0(11) |  | C_28_ | C_29_ | C_30_ | C_31_ | 178.7(6) |
| C_9_ | C_11_ | C_12_ | C_14_ | 159.3(8) |  | C_29_ | C_30_ | C_31_ | O_3_ | -4.2(10) |
| C_10_ | C_9_ | C_11_ | N_1_ | 174.0(9) |  | C_29_ | C_30_ | C_31_ | C_32_ | 177.4(7) |
| C_10_ | C_9_ | C_11_ | C_12_ | 4.5(15) |  | C_30_ | C_31_ | C_32_ | C_33_ | 177.5(7) |
| C_11_ | N_1_ | C_7_ | C_6_ | -178.3(6) |  | C_31_ | O_3_ | C_34_ | Br_2_ | -179.3(5) |
| C_11_ | N_1_ | C_7_ | C_8_ | -3.5(8) |  | C_31_ | O_3_ | C_34_ | C_33_ | 0.5(8) |
| C_11_ | N_1_ | B_1_ | F_4_ | -82.2(7) |  | C_31_ | C_32_ | C_33_ | C_34_ | 1.2(8) |
| C_11_ | N_1_ | B_1_ | F_5_ | 156.8(6) |  | C_32_ | C_33_ | C_34_ | Br_2_ | 178.6(6) |
| C_11_ | N_1_ | B_1_ | N_2_ | 36.5(8) |  | C_32_ | C_33_ | C_34_ | O_3_ | -1.1(9) |
| C_11_ | C_12_ | C_13_ | F_1_ | 89.0(8) |  | C_34_ | O_3_ | C_31_ | C_30_ | -178.4(6) |
| C_11_ | C_12_ | C_13_ | F_2_ | -151.2(6) |  | C_34_ | O_3_ | C_31_ | C_32_ | 0.3(7) |
| C_11_ | C_12_ | C_13_ | F_3_ | -33.0(8) |  | C_35_ | N_3_ | C_28_ | C_27_ | -2.8(7) |
| C_11_ | C_12_ | C_14_ | N_2_ | 16.1(9) |  | C_35_ | N_3_ | C_28_ | C_29_ | 176.3(6) |
| C_11_ | C_12_ | C_14_ | C_15_ | -156.9(7) |  | C_35_ | N_3_ | B_2_ | F_9_ | 149.6(5) |
| C_12_ | C_14_ | C_15_ | C_16_ | 174.3(7) |  | C_35_ | N_3_ | B_2_ | F_10_ | -88.6(7) |
| C_12_ | C_14_ | C_15_ | C_24_ | 2.2(12) |  | C_35_ | N_3_ | B_2_ | N_4_ | 28.3(8) |
| C_13_ | C_12_ | C_14_ | N_2_ | -159.5(6) |  | C_35_ | C_26_ | C_27_ | C_28_ | -1.7(7) |
| C_13_ | C_12_ | C_14_ | C_15_ | 27.5(11) |  | C_35_ | C_36_ | C_37_ | N_4_ | 11.1(10) |
| C_14_ | N_2_ | C_17_ | C_16_ | 3.0(7) |  | C_35_ | C_36_ | C_37_ | C_38_ | -160.1(8) |
| C_14_ | N_2_ | C_17_ | C_18_ | -175.8(6) |  | C_36_ | C_37_ | C_38_ | C_39_ | -2.0(14) |
| C_14_ | N_2_ | B_1_ | F_4_ | 88.6(7) |  | C_36_ | C_37_ | C_38_ | C_40_ | 171.5(8) |
| C_14_ | N_2_ | B_1_ | F_5_ | -151.2(5) |  | C_37_ | N_4_ | C_41_ | C_40_ | 1.9(8) |
| C_14_ | N_2_ | B_1_ | N_1_ | -29.8(7) |  | C_37_ | N_4_ | C_41_ | C_42_ | -179.8(6) |
| C_14_ | C_12_ | C_13_ | F_1_ | -95.2(8) |  | C_37_ | N_4_ | B_2_ | F_9_ | -155.9(6) |
| C_14_ | C_12_ | C_13_ | F_2_ | 24.6(9) |  | C_37_ | N_4_ | B_2_ | F_10_ | 83.4(7) |
| C_14_ | C_12_ | C_13_ | F_3_ | 142.8(6) |  | C_37_ | N_4_ | B_2_ | N_3_ | -34.2(8) |
| C_14_ | C_15_ | C_16_ | C_17_ | 1.1(7) |  | C_37_ | C_38_ | C_40_ | C_41_ | 1.6(9) |
| C_15_ | C_16_ | C_17_ | N_2_ | -2.6(8) |  | C_38_ | C_40_ | C_41_ | N_4_ | -2.2(9) |
| C_15_ | C_16_ | C_17_ | C_18_ | 176.2(6) |  | C_38_ | C_40_ | C_41_ | C_42_ | 179.6(7) |
| C_16_ | C_17_ | C_18_ | C_19_ | 13.6(11) |  | C_39_ | C_38_ | C_40_ | C_41_ | 175.5(8) |
| C_17_ | N_2_ | C_14_ | C_12_ | -177.0(6) |  | C_40_ | C_41_ | C_42_ | C_43_ | -3.2(12) |
| C_17_ | N_2_ | C_14_ | C_15_ | -2.3(7) |  | C_41_ | N_4_ | C_37_ | C_36_ | -174.2(6) |
| C_17_ | N_2_ | B_1_ | F_4_ | -87.4(7) |  | C_41_ | N_4_ | C_37_ | C_38_ | -0.9(8) |
| C_17_ | N_2_ | B_1_ | F_5_ | 32.8(9) |  | C_41_ | N_4_ | B_2_ | F_9_ | 38.2(9) |
| C_17_ | N_2_ | B_1_ | N_1_ | 154.2(6) |  | C_41_ | N_4_ | B_2_ | F_10_ | -82.5(7) |
| C_17_ | C_18_ | C_19_ | C_20_ | -178.7(6) |  | C_41_ | N_4_ | B_2_ | N_3_ | 159.9(6) |
| C_18_ | C_19_ | C_20_ | O_2_ | 3.0(11) |  | C_41_ | C_42_ | C_43_ | C_44_ | 174.1(7) |
| C_18_ | C_19_ | C_20_ | C_21_ | -175.1(8) |  | C_42_ | C_43_ | C_44_ | O_4_ | 2.3(11) |
| C_19_ | C_20_ | C_21_ | C_22_ | 178.7(8) |  | C_42_ | C_43_ | C_44_ | C_45_ | -176.0(8) |
| C_20_ | O_2_ | C_23_ | Br_3_ | 179.5(5) |  | C_43_ | C_44_ | C_45_ | C_46_ | 178.7(8) |
| C_20_ | O_2_ | C_23_ | C_22_ | 0.9(8) |  | C_44_ | O_4_ | C_47_ | Br_4_ | -178.7(4) |
| C_20_ | C_21_ | C_22_ | C_23_ | 0.1(9) |  | C_44_ | O_4_ | C_47_ | C_46_ | -2.2(8) |
| C_21_ | C_22_ | C_23_ | Br_3_ | -179.0(6) |  | C_44_ | C_45_ | C_46_ | C_47_ | -1.6(9) |
| C_21_ | C_22_ | C_23_ | O_2_ | -0.6(9) |  | C_45_ | C_46_ | C_47_ | Br_4_ | 178.0(6) |
| C_23_ | O_2_ | C_20_ | C_19_ | -179.3(6) |  | C_45_ | C_46_ | C_47_ | O_4_ | 2.4(9) |
| C_23_ | O_2_ | C_20_ | C_21_ | -0.8(8) |  | C_47_ | O_4_ | C_44_ | C_43_ | -177.6(6) |
| C_24_ | C_15_ | C_16_ | C_17_ | 173.5(6) |  | C_47_ | O_4_ | C_44_ | C_45_ | 1.1(8) |
| B_1_ | N_1_ | C_7_ | C_6_ | 17.0(10) |  | B_2_ | N_3_ | C_28_ | C_27_ | -178.8(6) |
| B_1_ | N_1_ | C_7_ | C_8_ | -168.2(6) |  | B_2_ | N_3_ | C_28_ | C_29_ | 0.3(10) |
| B_1_ | N_1_ | C_11_ | C_9_ | 168.9(6) |  | B_2_ | N_3_ | C_35_ | C_26_ | 177.9(5) |
| B_1_ | N_1_ | C_11_ | C_12_ | -19.7(9) |  | B_2_ | N_3_ | C_35_ | C_36_ | -5.3(9) |
| B_1_ | N_2_ | C_14_ | C_12_ | 6.3(9) |  | B_2_ | N_4_ | C_37_ | C_36_ | 17.4(9) |
| B_1_ | N_2_ | C_14_ | C_15_ | -179.0(5) |  | B_2_ | N_4_ | C_37_ | C_38_ | -169.3(6) |
| B_1_ | N_2_ | C_17_ | C_16_ | 179.4(6) |  | B_2_ | N_4_ | C_41_ | C_40_ | 169.2(6) |
| B_1_ | N_2_ | C_17_ | C_18_ | 0.6(10) |  | B_2_ | N_4_ | C_41_ | C_42_ | -12.5(10) |

| Table 7 Hydrogen Atom Coordinates (Å×10^4^) and Isotropic Displacement Parameters (Å^2^×10^3^) for OB-BDP. | | | | |
| --- | --- | --- | --- | --- |
| Atom | *x* | *y* | *z* | U(eq) |
| H_2_ | -493.17 | -4102.85 | 4352.25 | 59 |
| H_3_ | -1783.9 | -4392.99 | 3390.68 | 57 |
| H_5_ | -1646.92 | -3014.01 | 2547.61 | 51 |
| H_6_ | 160.32 | -906.67 | 2839.59 | 43 |
| H_8_ | -2750.84 | -2484.02 | 1738.88 | 59 |
| H_10A_ | -3089.55 | -1736.79 | 495.53 | 110 |
| H_10B_ | -4373.54 | -857.26 | 706.94 | 110 |
| H_10C_ | -4316.12 | -2187.53 | 860.9 | 110 |
| H_16_ | 149.01 | 4726.1 | 1773.49 | 49 |
| H_18_ | 2371.3 | 2771.8 | 2731.3 | 45 |
| H_19_ | 1446.98 | 5101.61 | 2642.26 | 50 |
| H_21_ | 2868.19 | 6607.09 | 3505.23 | 58 |
| H_22_ | 4941.29 | 6273.04 | 4290.83 | 64 |
| H_24A_ | -1276.02 | 3786.16 | 553.55 | 81 |
| H_24B_ | -2057.43 | 4628.69 | 970.46 | 81 |
| H_24C_ | -2856.03 | 3358.68 | 774.5 | 81 |
| H_25A_ | 2124.57 | 1859.46 | 776.12 | 79 |
| H_25B_ | 3030.51 | 703.94 | 954.77 | 79 |
| H_25C_ | 3708.97 | 1455.03 | 535.68 | 79 |
| H_27_ | 5173.41 | 983.62 | 1762.81 | 50 |
| H_29_ | 7308.2 | 3394.68 | 2721.11 | 49 |
| H_30_ | 6504.62 | 984.62 | 2627.72 | 53 |
| H_32_ | 8018.21 | -142.05 | 3496.4 | 61 |
| H_33_ | 10172.31 | 591.63 | 4254.19 | 63 |
| H_39A_ | 555.19 | 7388.1 | 845.8 | 109 |
| H_39B_ | 683.85 | 6020.24 | 662.69 | 109 |
| H_39C_ | 1870.86 | 6967.31 | 482.43 | 109 |
| H_40_ | 2201.52 | 8129.72 | 1712.62 | 55 |
| H_42_ | 5158.1 | 7061.32 | 2818.16 | 50 |
| H_43_ | 3372.05 | 9033.46 | 2514.51 | 48 |
| H_45_ | 3369.62 | 10773.4 | 3362.05 | 59 |
| H_46_ | 4698.25 | 10916.38 | 4296.21 | 63 |

Experimental

Single crystals of C_24_H_16_BBr_2_F_5_N_2_O_2_ [OB-BDP] were []. A suitable crystal was selected and [] on a ROD, Synergy Custom system, HyPix-Arc 150 diffractometer. The crystal was kept at 200.03(10) K during data collection. Using Olex2 [1], the structure was solved with the SHELXS [2] structure solution program using Direct Methods and refined with the SHELXL [3] refinement package using Least Squares minimisation.

1. Dolomanov, O.V., Bourhis, L.J., Gildea, R.J, Howard, J.A.K. & Puschmann, H. (2009), J. Appl. Cryst. 42, 339-341.
2. Sheldrick, G.M. (2008). Acta Cryst. A64, 112-122.
3. Sheldrick, G.M. (2015). Acta Cryst. C71, 3-8.

Refinement model description

Number of restraints - 0, number of constraints - unknown.

Details:

1. Fixed Uiso
    At 1.2 times of:
    All C(H) groups
    At 1.5 times of:
    All C(H,H,H) groups
   2.a Aromatic/amide H refined with riding coordinates:
    C2(H2), C3(H3), C5(H5), C6(H6), C8(H8), C16(H16), C18(H18), C19(H19),
    C21(H21), C22(H22), C27(H27), C29(H29), C30(H30), C32(H32), C33(H33), C40(H40),
    C42(H42), C43(H43), C45(H45), C46(H46)
   2.b Idealised Me refined as rotating group:
    C10(H10A,H10B,H10C), C24(H24A,H24B,H24C), C25(H25A,H25B,H25C), C39(H39A,H39B,
    H39C)

**OH-BDP**

| **Table 1 Crystal data and structure refinement for GSY_O_tw.** | |
| --- | --- |
| Identification code | GSY_O_tw |
| Empirical formula | C_24_H_18_BF_5_N_2_O_2_ |
| Formula weight | 472.21 |
| Temperature/K | 120.00(10) |
| Crystal system | monoclinic |
| Space group | P2_1_/c |
| a/Å | 7.66883(14) |
| b/Å | 24.0343(5) |
| c/Å | 11.3023(3) |
| α/° | 90 |
| β/° | 90.200(2) |
| γ/° | 90 |
| Volume/Å^3^ | 2083.18(7) |
| Z | 4 |
| ρ_calc_g/cm^3^ | 1.506 |
| μ/mm^‑1^ | 1.086 |
| F(000) | 968.0 |
| Crystal size/mm^3^ | 0.15 × 0.14 × 0.12 |
| Radiation | Cu Kα (λ = 1.54184) |
| 2Θ range for data collection/° | 7.356 to 133.2 |
| Index ranges | -9 ≤ h ≤ 9, -28 ≤ k ≤ 28, 0 ≤ l ≤ 13 |
| Reflections collected | 3672 |
| Independent reflections | 3672 [R_int_ = 0.0394, R_sigma_ = 0.0238] |
| Data/restraints/parameters | 3672/0/310 |
| Goodness-of-fit on F^2^ | 1.260 |
| Final R indexes [I>=2σ (I)] | R_1_ = 0.0857, wR_2_ = 0.2362 |
| Final R indexes [all data] | R_1_ = 0.0892, wR_2_ = 0.2377 |
| Largest diff. peak/hole / e Å^-3^ | 0.49/-0.49 |

**Crystal structure determination of OH-BDP**

**Crystal Data** for C_24_H_18_BF_5_N_2_O_2_ (*M*=472.21 g/mol): monoclinic, space group P2_1_/c (no. 14), *a* = 7.66883(14) Å, *b* = 24.0343(5) Å, *c* = 11.3023(3) Å, *β* = 90.200(2)°, *V*= 2083.18(7) Å^3^, *Z* = 4, *T* = 120.00(10) K, μ(Cu Kα) = 1.086 mm^-1^, *Dcalc* = 1.506 g/cm^3^, 3672 reflections measured (7.356° ≤ 2Θ ≤ 133.2°), 3672 unique (*R*_int_ = 0.0394, R_sigma_ = 0.0238) which were used in all calculations. The final *R*_1_ was 0.0857 (I > 2σ(I)) and *wR*_2_ was 0.2377 (all data).

**Refinement model description**

| **Table 2 Fractional Atomic Coordinates (×10^4^) and Equivalent Isotropic Displacement Parameters (Å^2^×10^3^) forOH-BDP. U_eq_ is defined as 1/3 of the trace of the orthogonalised U_IJ_ tensor.** | | | | |
| --- | --- | --- | --- | --- |
| **Atom** | ***x*** | ***y*** | ***z*** | **U(eq)** |
| F1 | 2525(5) | 2003.0(15) | 4826(3) | 37.6(9) |
| F2 | 2386(5) | 2456.3(16) | 3186(3) | 34.2(9) |
| F3 | 4859(5) | 2310.8(16) | 4039(4) | 38.0(10) |
| F4 | 2543(5) | 4417.0(14) | 6783(3) | 27.8(8) |
| F5 | 3938(5) | 3738.1(16) | 7809(3) | 29.6(8) |
| O1 | -1053(6) | 4469.6(19) | 10117(4) | 31.8(10) |
| O2 | 7453(6) | 5496.8(19) | 7639(4) | 35.0(11) |
| N1 | 1576(6) | 3479(2) | 6496(4) | 22.7(11) |
| N2 | 4389(6) | 3797(2) | 5697(4) | 23.1(11) |
| C1 | -2382(8) | 4202(3) | 9543(5) | 25.9(13) |
| C2 | -3892(9) | 4296(3) | 10120(6) | 34.5(16) |
| C3 | -3531(10) | 4634(3) | 11101(7) | 39.2(17) |
| C4 | -1796(9) | 4729(3) | 11076(6) | 33.7(15) |
| C5 | -1947(8) | 3881(3) | 8517(5) | 25.4(13) |
| C6 | -369(8) | 3847(3) | 8032(5) | 24.7(13) |
| C7 | 14(8) | 3491(3) | 7055(5) | 23.4(13) |
| C8 | -1069(8) | 3078(3) | 6549(6) | 27.8(14) |
| C9 | -139(8) | 2784(3) | 5714(6) | 27.6(13) |
| C10 | -895(9) | 2325(3) | 4987(6) | 36.8(16) |
| C11 | 1559(7) | 3039(2) | 5673(5) | 21.2(12) |
| C12 | 2998(8) | 2970(3) | 4933(5) | 25.7(13) |
| C13 | 3189(8) | 2435(3) | 4236(6) | 27.2(13) |
| C14 | 4310(8) | 3380(3) | 4850(6) | 24.5(13) |
| C15 | 5568(8) | 3505(3) | 3936(6) | 28.3(14) |
| C16 | 6389(8) | 3990(3) | 4281(6) | 28.3(14) |
| C17 | 5700(7) | 4157(3) | 5382(5) | 24.2(13) |
| C18 | 6243(8) | 4611(2) | 6128(6) | 24.9(13) |
| C19 | 7444(8) | 4992(3) | 5792(6) | 26.7(13) |
| C20 | 8038(8) | 5449(3) | 6497(6) | 27.3(14) |
| C21 | 9078(9) | 5886(3) | 6240(6) | 31.9(15) |
| C22 | 9150(9) | 6225(3) | 7264(7) | 37.8(17) |
| C23 | 8146(9) | 5979(3) | 8082(7) | 39.5(17) |
| C24 | 5919(9) | 3227(3) | 2771(6) | 35.2(16) |
| B1 | 3124(9) | 3868(3) | 6738(6) | 22.3(14) |

| **Table 3 Anisotropic Displacement Parameters (Å^2^×10^3^) for OH-BDP. The Anisotropic displacement factor exponent takes the form: -2π^2^[h^2^a*^2^U_11_+2hka*b*U_12_+…].** | | | | | | |
| --- | --- | --- | --- | --- | --- | --- |
| **Atom** | **U_11_** | **U_22_** | **U_33_** | **U_23_** | **U_13_** | **U_12_** |
| F1 | 43(2) | 26.8(19) | 43(2) | -3.5(17) | -0.2(19) | 0.2(17) |
| F2 | 32.1(19) | 41(2) | 29.4(19) | -11.8(16) | -3.3(16) | 1.3(18) |
| F3 | 29.5(19) | 36(2) | 48(2) | -11.2(19) | 2.4(18) | 4.9(17) |
| F4 | 23.0(17) | 27.4(18) | 33.0(19) | -2.3(15) | 5.9(15) | -0.7(15) |
| F5 | 23.5(18) | 43(2) | 21.9(18) | 2.3(15) | -1.7(14) | -6.3(16) |
| O1 | 25(2) | 42(3) | 29(2) | -6(2) | 9.8(18) | -5(2) |
| O2 | 30(2) | 40(3) | 36(2) | -5(2) | 0(2) | -4(2) |
| N1 | 20(2) | 24(3) | 23(2) | 1(2) | -4(2) | 1(2) |
| N2 | 20(2) | 26(3) | 24(3) | 0(2) | -2(2) | -1(2) |
| C1 | 22(3) | 29(3) | 26(3) | 1(2) | -2(3) | 1(3) |
| C2 | 19(3) | 50(4) | 34(4) | 1(3) | 11(3) | -1(3) |
| C3 | 36(4) | 40(4) | 42(4) | -5(3) | 11(3) | 5(3) |
| C4 | 38(4) | 38(4) | 25(3) | -6(3) | 7(3) | 0(3) |
| C5 | 22(3) | 27(3) | 27(3) | 6(3) | -8(2) | 1(2) |
| C6 | 23(3) | 26(3) | 25(3) | 7(3) | 2(2) | -1(2) |
| C7 | 20(3) | 28(3) | 22(3) | 4(2) | -1(2) | 2(2) |
| C8 | 18(3) | 32(3) | 33(3) | 2(3) | -1(3) | -4(3) |
| C9 | 21(3) | 33(3) | 29(3) | 1(3) | -1(2) | -3(3) |
| C10 | 29(3) | 42(4) | 39(4) | -9(3) | 2(3) | -3(3) |
| C11 | 17(3) | 25(3) | 22(3) | 2(2) | 2(2) | 0(2) |
| C12 | 27(3) | 28(3) | 22(3) | 1(2) | -1(2) | 1(3) |
| C13 | 20(3) | 30(3) | 32(3) | -3(3) | -7(3) | 2(3) |
| C14 | 18(3) | 26(3) | 29(3) | -2(3) | -4(2) | 4(2) |
| C15 | 28(3) | 32(3) | 26(3) | 0(3) | -4(3) | 9(3) |
| C16 | 20(3) | 34(3) | 31(3) | 2(3) | 10(3) | 1(3) |
| C17 | 16(3) | 30(3) | 27(3) | 3(3) | 3(2) | 3(2) |
| C18 | 19(3) | 27(3) | 28(3) | 2(3) | -4(2) | 4(3) |
| C19 | 22(3) | 30(3) | 29(3) | 2(3) | 1(3) | 4(3) |
| C20 | 22(3) | 29(3) | 31(3) | 3(3) | -5(3) | 2(3) |
| C21 | 29(3) | 34(4) | 33(4) | 7(3) | -2(3) | 0(3) |
| C22 | 27(3) | 31(4) | 55(5) | -4(3) | -10(3) | -5(3) |
| C23 | 28(3) | 41(4) | 50(4) | -11(3) | 3(3) | 0(3) |
| C24 | 32(4) | 43(4) | 31(4) | -6(3) | 3(3) | 5(3) |
| B1 | 18(3) | 26(3) | 23(3) | 0(3) | -1(3) | -6(3) |

| **Table 4 Bond Lengths for OH-BDP**  **.** | | | | | | |
| --- | --- | --- | --- | --- | --- | --- |
| **Atom** | **Atom** | **Length/Å** |  | **Atom** | **Atom** | **Length/Å** |
| F1 | C13 | 1.336(8) |  | C5 | C6 | 1.333(9) |
| F2 | C13 | 1.336(7) |  | C6 | C7 | 1.428(9) |
| F3 | C13 | 1.335(7) |  | C7 | C8 | 1.414(9) |
| F4 | B1 | 1.392(8) |  | C8 | C9 | 1.380(9) |
| F5 | B1 | 1.396(8) |  | C9 | C10 | 1.491(9) |
| O1 | C1 | 1.367(8) |  | C9 | C11 | 1.440(8) |
| O1 | C4 | 1.375(8) |  | C11 | C12 | 1.397(8) |
| O2 | C20 | 1.373(8) |  | C12 | C13 | 1.515(9) |
| O2 | C23 | 1.369(9) |  | C12 | C14 | 1.412(9) |
| N1 | C7 | 1.356(8) |  | C14 | C15 | 1.447(9) |
| N1 | C11 | 1.408(8) |  | C15 | C16 | 1.380(9) |
| N1 | B1 | 1.536(8) |  | C15 | C24 | 1.503(9) |
| N2 | C14 | 1.386(8) |  | C16 | C17 | 1.412(9) |
| N2 | C17 | 1.374(8) |  | C17 | C18 | 1.439(9) |
| N2 | B1 | 1.538(8) |  | C18 | C19 | 1.354(9) |
| C1 | C2 | 1.350(9) |  | C19 | C20 | 1.431(9) |
| C1 | C5 | 1.433(9) |  | C20 | C21 | 1.351(9) |
| C2 | C3 | 1.401(10) |  | C21 | C22 | 1.415(10) |
| C3 | C4 | 1.351(10) |  | C22 | C23 | 1.342(11) |

| **Table 5 Bond Angles for OH-BDP**  **.** | | | | | | | | |
| --- | --- | --- | --- | --- | --- | --- | --- | --- |
| **Atom** | **Atom** | **Atom** | **Angle/˚** |  | **Atom** | **Atom** | **Atom** | **Angle/˚** |
| C1 | O1 | C4 | 106.1(5) |  | F1 | C13 | C12 | 111.3(5) |
| C23 | O2 | C20 | 106.7(5) |  | F2 | C13 | C12 | 112.6(5) |
| C7 | N1 | C11 | 108.6(5) |  | F3 | C13 | F1 | 106.1(5) |
| C7 | N1 | B1 | 125.9(5) |  | F3 | C13 | F2 | 107.4(5) |
| C11 | N1 | B1 | 125.5(5) |  | F3 | C13 | C12 | 111.8(5) |
| C14 | N2 | B1 | 125.6(5) |  | N2 | C14 | C12 | 119.2(5) |
| C17 | N2 | C14 | 107.9(5) |  | N2 | C14 | C15 | 108.4(5) |
| C17 | N2 | B1 | 126.3(5) |  | C12 | C14 | C15 | 132.0(6) |
| O1 | C1 | C5 | 117.5(5) |  | C14 | C15 | C24 | 130.9(6) |
| C2 | C1 | O1 | 109.4(5) |  | C16 | C15 | C14 | 106.1(5) |
| C2 | C1 | C5 | 133.1(6) |  | C16 | C15 | C24 | 122.7(6) |
| C1 | C2 | C3 | 108.2(6) |  | C15 | C16 | C17 | 108.5(6) |
| C4 | C3 | C2 | 105.8(6) |  | N2 | C17 | C16 | 108.9(5) |
| C3 | C4 | O1 | 110.6(6) |  | N2 | C17 | C18 | 122.4(5) |
| C6 | C5 | C1 | 125.4(6) |  | C16 | C17 | C18 | 128.6(6) |
| C5 | C6 | C7 | 123.0(6) |  | C19 | C18 | C17 | 122.9(6) |
| N1 | C7 | C6 | 123.9(6) |  | C18 | C19 | C20 | 125.4(6) |
| N1 | C7 | C8 | 108.3(5) |  | O2 | C20 | C19 | 118.9(6) |
| C8 | C7 | C6 | 127.7(6) |  | C21 | C20 | O2 | 109.4(6) |
| C9 | C8 | C7 | 109.4(5) |  | C21 | C20 | C19 | 131.7(6) |
| C8 | C9 | C10 | 123.7(6) |  | C20 | C21 | C22 | 107.0(6) |
| C8 | C9 | C11 | 105.9(5) |  | C23 | C22 | C21 | 106.8(6) |
| C11 | C9 | C10 | 130.3(6) |  | C22 | C23 | O2 | 110.1(7) |
| N1 | C11 | C9 | 107.7(5) |  | F4 | B1 | F5 | 108.9(5) |
| C12 | C11 | N1 | 118.6(5) |  | F4 | B1 | N1 | 109.7(5) |
| C12 | C11 | C9 | 133.2(6) |  | F4 | B1 | N2 | 109.7(5) |
| C11 | C12 | C13 | 119.3(5) |  | F5 | B1 | N1 | 111.1(5) |
| C11 | C12 | C14 | 121.4(6) |  | F5 | B1 | N2 | 111.0(5) |
| C14 | C12 | C13 | 119.2(5) |  | N1 | B1 | N2 | 106.6(5) |
| F1 | C13 | F2 | 107.3(5) |  |  |  |  |  |

| **Table 6 Torsion Angles for OH-BDP**  **.** | | | | | | | | | | |
| --- | --- | --- | --- | --- | --- | --- | --- | --- | --- | --- |
| **A** | **B** | **C** | **D** | **Angle/˚** |  | **A** | **B** | **C** | **D** | **Angle/˚** |
| O1 | C1 | C2 | C3 | -0.1(8) |  | C11 | C12 | C14 | N2 | 14.6(9) |
| O1 | C1 | C5 | C6 | -4.2(9) |  | C11 | C12 | C14 | C15 | -157.0(6) |
| O2 | C20 | C21 | C22 | -0.1(7) |  | C12 | C14 | C15 | C16 | 173.5(6) |
| N1 | C7 | C8 | C9 | 3.5(7) |  | C12 | C14 | C15 | C24 | -1.6(11) |
| N1 | C11 | C12 | C13 | 166.8(5) |  | C13 | C12 | C14 | N2 | -163.3(5) |
| N1 | C11 | C12 | C14 | -11.0(9) |  | C13 | C12 | C14 | C15 | 25.2(10) |
| N2 | C14 | C15 | C16 | 1.2(7) |  | C14 | N2 | C17 | C16 | 3.9(7) |
| N2 | C14 | C15 | C24 | -173.8(6) |  | C14 | N2 | C17 | C18 | -174.1(5) |
| N2 | C17 | C18 | C19 | -175.1(6) |  | C14 | N2 | B1 | F4 | -130.8(6) |
| C1 | O1 | C4 | C3 | -0.5(8) |  | C14 | N2 | B1 | F5 | 109.0(6) |
| C1 | C2 | C3 | C4 | -0.2(9) |  | C14 | N2 | B1 | N1 | -12.1(8) |
| C1 | C5 | C6 | C7 | 175.7(6) |  | C14 | C12 | C13 | F1 | 145.7(5) |
| C2 | C1 | C5 | C6 | 177.7(7) |  | C14 | C12 | C13 | F2 | -93.8(7) |
| C2 | C3 | C4 | O1 | 0.5(9) |  | C14 | C12 | C13 | F3 | 27.3(8) |
| C4 | O1 | C1 | C2 | 0.4(7) |  | C14 | C15 | C16 | C17 | 1.1(7) |
| C4 | O1 | C1 | C5 | -178.2(5) |  | C15 | C16 | C17 | N2 | -3.1(7) |
| C5 | C1 | C2 | C3 | 178.2(7) |  | C15 | C16 | C17 | C18 | 174.7(6) |
| C5 | C6 | C7 | N1 | 175.2(6) |  | C16 | C17 | C18 | C19 | 7.3(10) |
| C5 | C6 | C7 | C8 | -8.8(10) |  | C17 | N2 | C14 | C12 | -176.6(5) |
| C6 | C7 | C8 | C9 | -173.0(6) |  | C17 | N2 | C14 | C15 | -3.2(6) |
| C7 | N1 | C11 | C9 | 2.0(6) |  | C17 | N2 | B1 | F4 | 43.4(8) |
| C7 | N1 | C11 | C12 | 175.7(5) |  | C17 | N2 | B1 | F5 | -76.9(7) |
| C7 | N1 | B1 | F4 | -47.1(8) |  | C17 | N2 | B1 | N1 | 162.0(5) |
| C7 | N1 | B1 | F5 | 73.3(7) |  | C17 | C18 | C19 | C20 | -179.7(6) |
| C7 | N1 | B1 | N2 | -165.8(5) |  | C18 | C19 | C20 | O2 | 4.5(9) |
| C7 | C8 | C9 | C10 | -178.5(6) |  | C18 | C19 | C20 | C21 | -172.4(7) |
| C7 | C8 | C9 | C11 | -2.2(7) |  | C19 | C20 | C21 | C22 | 177.1(7) |
| C8 | C9 | C11 | N1 | 0.2(7) |  | C20 | O2 | C23 | C22 | -0.9(8) |
| C8 | C9 | C11 | C12 | -172.2(7) |  | C20 | C21 | C22 | C23 | -0.4(8) |
| C9 | C11 | C12 | C13 | -21.5(10) |  | C21 | C22 | C23 | O2 | 0.8(8) |
| C9 | C11 | C12 | C14 | 160.7(6) |  | C23 | O2 | C20 | C19 | -177.0(6) |
| C10 | C9 | C11 | N1 | 176.2(6) |  | C23 | O2 | C20 | C21 | 0.6(7) |
| C10 | C9 | C11 | C12 | 3.8(12) |  | C24 | C15 | C16 | C17 | 176.7(6) |
| C11 | N1 | C7 | C6 | 173.3(5) |  | B1 | N1 | C7 | C6 | -5.3(9) |
| C11 | N1 | C7 | C8 | -3.3(7) |  | B1 | N1 | C7 | C8 | 178.1(5) |
| C11 | N1 | B1 | F4 | 134.5(5) |  | B1 | N1 | C11 | C9 | -179.4(5) |
| C11 | N1 | B1 | F5 | -105.2(6) |  | B1 | N1 | C11 | C12 | -5.7(8) |
| C11 | N1 | B1 | N2 | 15.8(8) |  | B1 | N2 | C14 | C12 | -1.5(9) |
| C11 | C12 | C13 | F1 | -32.2(8) |  | B1 | N2 | C14 | C15 | 171.9(5) |
| C11 | C12 | C13 | F2 | 88.3(7) |  | B1 | N2 | C17 | C16 | -171.1(5) |
| C11 | C12 | C13 | F3 | -150.6(6) |  | B1 | N2 | C17 | C18 | 10.9(9) |

| **Table 7 Hydrogen Atom Coordinates (Å×10^4^) and Isotropic Displacement Parameters (Å^2^×10^3^) for OH-BDP**  **.** | | | | |
| --- | --- | --- | --- | --- |
| **Atom** | ***x*** | ***y*** | ***z*** | **U(eq)** |
| H2 | -5006.51 | 4157.01 | 9899.42 | 41 |
| H3 | -4340.38 | 4768.21 | 11668.1 | 47 |
| H4 | -1174.91 | 4945.03 | 11640.86 | 40 |
| H5 | -2860.39 | 3675.96 | 8151.51 | 30 |
| H6 | 542.23 | 4070 | 8350.83 | 30 |
| H8 | -2252.7 | 3013.2 | 6751.96 | 33 |
| H10A | -459.86 | 1967.17 | 5277.66 | 55 |
| H10B | -553.57 | 2374.1 | 4158.52 | 55 |
| H10C | -2169.52 | 2333.73 | 5046.5 | 55 |
| H16 | 7271 | 4178.67 | 3850.79 | 34 |
| H18 | 5735.84 | 4644.76 | 6890.2 | 30 |
| H19 | 7932.12 | 4952.28 | 5025.21 | 32 |
| H21 | 9652.29 | 5953.58 | 5510.93 | 38 |
| H22 | 9787.54 | 6560.89 | 7354.06 | 45 |
| H23 | 7945.38 | 6119.31 | 8855.45 | 47 |
| H24A | 6592.46 | 3477.81 | 2264.72 | 53 |
| H24B | 4810.13 | 3136.24 | 2382.2 | 53 |
| H24C | 6583.64 | 2883.93 | 2904.98 | 53 |

**Experimental**

Single crystals of C_24_H_18_BF_5_N_2_O_2_ **OH-BDP**

 were **[]**. A suitable crystal was selected and **[]** on a **ROD, Synergy Custom system, HyPix** diffractometer. The crystal was kept at 120.00(10) K during data collection. Using Olex2 [1], the structure was solved with the SHELXS [2] structure solution program using Direct Methods and refined with the SHELXL [3] refinement package using Least Squares minimisation.

1. Dolomanov, O.V., Bourhis, L.J., Gildea, R.J, Howard, J.A.K. & Puschmann, H. (2009), J. Appl. Cryst. 42, 339-341.
2. Sheldrick, G.M. (2008). Acta Cryst. A64, 112-122.
3. Sheldrick, G.M. (2015). Acta Cryst. C71, 3-8.

Number of restraints - 0, number of constraints - unknown.

Details:

1. Twinned data refinement
 Scales: 0.723(3)
 0.277(3)
2. Fixed Uiso
 At 1.2 times of:
 All C(H) groups
 At 1.5 times of:
 All C(H,H,H) groups
3.a Aromatic/amide H refined with riding coordinates:
 C2(H2), C3(H3), C4(H4), C5(H5), C6(H6), C8(H8), C16(H16), C18(H18), C19(H19),
 C21(H21), C22(H22), C23(H23)
3.b Idealised Me refined as rotating group:
 C10(H10A,H10B,H10C), C24(H24A,H24B,H24C)

**SB-BDP**

| **Table 1 Crystal data and structure refinement for SB-BDP.** | |
| --- | --- |
| Identification code | SB-BDP |
| Empirical formula | C_24_H_16_BBr_2_F_5_N_2_S_2_ |
| Formula weight | 662.14 |
| Temperature/K | 100.03(10) |
| Crystal system | monoclinic |
| Space group | P2_1_/c |
| a/Å | 7.53231(11) |
| b/Å | 22.4881(3) |
| c/Å | 14.11510(20) |
| α/° | 90 |
| β/° | 95.3957(13) |
| γ/° | 90 |
| Volume/Å^3^ | 2380.33(6) |
| Z | 4 |
| ρ_calc_g/cm^3^ | 1.848 |
| μ/mm^‑1^ | 6.484 |
| F(000) | 1304.0 |
| Crystal size/mm^3^ | 0.13 × 0.1 × 0.08 |
| Radiation | Cu Kα (λ = 1.54184) |
| 2Θ range for data collection/° | 7.418 to 146.016 |
| Index ranges | -9 ≤ h ≤ 8, -27 ≤ k ≤ 26, -15 ≤ l ≤ 16 |
| Reflections collected | 18194 |
| Independent reflections | 4595 [R_int_ = 0.0304, R_sigma_ = 0.0233] |
| Data/restraints/parameters | 4595/0/327 |
| Goodness-of-fit on F^2^ | 1.040 |
| Final R indexes [I>=2σ (I)] | R_1_ = 0.0292, wR_2_ = 0.0774 |
| Final R indexes [all data] | R_1_ = 0.0335, wR_2_ = 0.0796 |
| Largest diff. peak/hole / e Å^-3^ | 0.67/-0.69 |

Crystal structure determination of [SB-BDP]

Crystal Data for C_24_H_16_BBr_2_F_5_N_2_S_2_ (*M* =662.14 g/mol): monoclinic, space group P2_1_/c (no. 14), *a* = 7.53231(11) Å, *b* = 22.4881(3) Å, *c* = 14.11510(20) Å, *β* = 95.3957(13)°, *V* = 2380.33(6) Å^3^, *Z* = 4, *T* = 100.03(10) K, μ(Cu Kα) = 6.484 mm^-1^, *Dcalc* = 1.848 g/cm^3^, 18194 reflections measured (7.418° ≤ 2Θ ≤ 146.016°), 4595 unique (*R*_int_ = 0.0304, R_sigma_ = 0.0233) which were used in all calculations. The final *R*_1_ was 0.0292 (I > 2σ(I)) and *wR*_2_ was 0.0796 (all data).

| Table 2 Fractional Atomic Coordinates (×10^4^) and Equivalent Isotropic Displacement Parameters (Å^2^×10^3^) for SB-BDP. U_eq_ is defined as 1/3 of the trace of the orthogonalised U_IJ_ tensor. | | | | |
| --- | --- | --- | --- | --- |
| Atom | *x* | *y* | *z* | U(eq) |
| Br_1_ | 875.0(4) | 6388.5(2) | 8497.3(2) | 29.27(9) |
| Br_2_ | 12517.4(4) | 4926.0(2) | 11717.3(2) | 26.34(9) |
| S_1_ | 2452.0(8) | 5368.7(3) | 7340.7(4) | 23.02(14) |
| S_2_ | 11493.9(8) | 4412.6(3) | 9676.5(4) | 21.45(13) |
| F_1_ | 6130.1(19) | 2217.1(6) | 3820.2(11) | 26.6(3) |
| F_2_ | 8583(2) | 2523.1(6) | 3303.8(10) | 25.6(3) |
| F_3_ | 8629(2) | 1908.8(6) | 4478.5(10) | 25.7(3) |
| F_4_ | 6596.8(19) | 3517.5(7) | 7179.1(10) | 26.9(3) |
| F_5_ | 7896.5(19) | 4323.0(6) | 6561.5(10) | 23.5(3) |
| N_1_ | 6159(3) | 3681.7(9) | 5478.6(14) | 18.2(4) |
| N_2_ | 9102(3) | 3365.2(9) | 6239.6(14) | 18.5(4) |
| C_1_ | 1083(3) | 5981.4(11) | 7359.7(18) | 22.1(5) |
| C_2_ | 279(3) | 6124.9(11) | 6491.0(18) | 20.6(5) |
| C_3_ | 787(3) | 5734.2(10) | 5778.3(17) | 19.6(5) |
| C_4_ | 1981(3) | 5304.0(11) | 6118.2(17) | 18.8(5) |
| C_5_ | 2804(3) | 4870.5(10) | 5552.3(17) | 19.3(5) |
| C_6_ | 4079(3) | 4475.8(10) | 5858.8(17) | 19.5(5) |
| C_7_ | 4768(3) | 4054.4(10) | 5224.8(17) | 18.8(5) |
| C_8_ | 4125(3) | 3929.5(11) | 4277.2(17) | 20.7(5) |
| C_9_ | 5062(3) | 3454.7(11) | 3954.9(17) | 19.8(5) |
| C_10_ | 6359(3) | 3296.5(10) | 4726.5(16) | 18.8(5) |
| C_11_ | 7815(3) | 2901.6(10) | 4795.7(17) | 19.4(5) |
| C_12_ | 7804(3) | 2392.4(11) | 4094.0(17) | 21.6(5) |
| C_13_ | 9255(3) | 2965.8(10) | 5495.7(17) | 18.6(5) |
| C_14_ | 11077(3) | 2754.2(10) | 5571.5(17) | 20.0(5) |
| C_15_ | 12018(4) | 2350.5(11) | 4937.4(19) | 25.5(5) |
| C_16_ | 11931(3) | 3029.0(11) | 6352.4(17) | 21.0(5) |
| C_17_ | 10706(3) | 3399.6(10) | 6774.7(17) | 18.9(5) |
| C_18_ | 10994(3) | 3735.8(10) | 7643.8(17) | 19.1(5) |
| C_19_ | 12621(3) | 3779.1(10) | 8127.5(17) | 19.5(5) |
| C_20_ | 13053(3) | 4064.1(10) | 9032.2(17) | 19.6(5) |
| C_21_ | 14701(3) | 4060.4(11) | 9540.5(19) | 24.7(5) |
| C_22_ | 14718(4) | 4326.0(12) | 10442.4(19) | 29.2(6) |
| C_23_ | 13080(3) | 4528.8(11) | 10616.8(18) | 24.6(5) |
| C_24_ | 4727(3) | 3221.8(12) | 2953.7(17) | 25.3(5) |
| B_1_ | 7419(4) | 3729.4(12) | 6405.3(19) | 19.5(5) |

| Table 3 Anisotropic Displacement Parameters (Å^2^×10^3^) for SB-BDP. The Anisotropic displacement factor exponent takes the form: -2π^2^[h^2^a*^2^U_11_+2hka*b*U_12_+…]. | | | | | | |
| --- | --- | --- | --- | --- | --- | --- |
| Atom | U_11_ | U_22_ | U_33_ | U_23_ | U_13_ | U_12_ |
| Br_1_ | 34.73(17) | 32.93(16) | 21.62(16) | -6.24(10) | 10.39(11) | 3.64(11) |
| Br_2_ | 28.96(15) | 31.79(16) | 18.32(15) | -6.16(10) | 2.52(11) | 3.23(10) |
| S_1_ | 27.0(3) | 26.7(3) | 15.6(3) | -0.5(2) | 3.4(2) | 6.1(2) |
| S_2_ | 21.6(3) | 27.4(3) | 15.7(3) | -2.0(2) | 4.0(2) | 2.6(2) |
| F_1_ | 30.1(8) | 23.5(7) | 26.8(8) | -7.2(6) | 6.6(6) | -5.7(6) |
| F_2_ | 36.4(8) | 25.0(7) | 17.5(7) | -3.7(6) | 13.0(6) | -3.5(6) |
| F_3_ | 37.0(8) | 17.3(7) | 23.7(8) | -0.6(6) | 7.9(6) | 0.8(6) |
| F_4_ | 27.1(8) | 37.9(8) | 17.3(7) | 5.9(6) | 10.5(6) | 7.1(6) |
| F_5_ | 27.2(7) | 21.4(7) | 21.4(7) | -5.4(6) | -0.3(6) | 4.6(6) |
| N_1_ | 21.9(10) | 18.0(10) | 15.5(10) | -1.1(7) | 5.8(8) | -1.0(8) |
| N_2_ | 22.0(10) | 20.2(10) | 14.3(10) | -0.9(8) | 6.8(8) | 1.3(8) |
| C_1_ | 22.4(12) | 24.4(12) | 20.6(12) | -3.1(10) | 8.5(10) | -0.2(10) |
| C_2_ | 18.1(11) | 19.3(11) | 24.8(13) | 1.0(10) | 5.0(9) | -0.7(9) |
| C_3_ | 19.7(11) | 20.8(12) | 18.6(12) | 1.4(9) | 3.4(9) | -3.9(9) |
| C_4_ | 20.6(12) | 21.0(12) | 15.5(11) | -0.1(9) | 4.6(9) | -1.5(9) |
| C_5_ | 19.9(12) | 21.7(12) | 17.1(12) | -1.1(9) | 5.8(9) | -3.7(9) |
| C_6_ | 21.7(12) | 21.5(12) | 15.9(11) | -1.3(9) | 5.4(9) | -3.6(9) |
| C_7_ | 18.8(11) | 19.9(11) | 18.3(12) | 1.0(9) | 4.8(9) | -2.8(9) |
| C_8_ | 20.9(12) | 23.9(12) | 17.8(12) | -1.7(9) | 4.2(9) | -3.3(9) |
| C_9_ | 21.1(12) | 22.7(12) | 16.3(12) | -1.6(9) | 6.0(9) | -3.7(9) |
| C_10_ | 23.9(12) | 19.2(11) | 14.6(11) | -0.7(9) | 9.0(9) | -4.1(9) |
| C_11_ | 25.9(13) | 17.6(11) | 16.3(11) | 0.3(9) | 9.9(9) | -3.2(9) |
| C_12_ | 26.7(13) | 19.6(12) | 19.9(12) | -0.5(9) | 8.9(10) | -3.2(10) |
| C_13_ | 24.8(12) | 16.5(11) | 15.7(11) | 0.0(9) | 8.9(9) | -0.3(9) |
| C_14_ | 24.1(12) | 17.3(11) | 20.1(12) | 1.4(9) | 10.2(10) | 0.8(9) |
| C_15_ | 30.7(14) | 24.4(13) | 23.4(13) | -3.7(10) | 12.1(11) | 3.0(10) |
| C_16_ | 23.7(12) | 21.8(12) | 18.6(12) | 1.8(9) | 7.0(10) | 2.1(9) |
| C_17_ | 22.0(12) | 18.7(11) | 16.4(12) | 2.2(9) | 4.6(9) | 0.7(9) |
| C_18_ | 22.4(12) | 19.6(11) | 16.1(12) | 1.5(9) | 6.6(9) | 2.2(9) |
| C_19_ | 23.7(12) | 17.5(11) | 18.3(12) | 0.0(9) | 7.4(9) | 1.5(9) |
| C_20_ | 21.6(12) | 18.8(11) | 19.2(12) | 0.1(9) | 6.2(9) | 0.8(9) |
| C_21_ | 22.4(12) | 24.6(13) | 27.3(14) | -3.0(10) | 3.2(10) | 2.5(10) |
| C_22_ | 27.3(13) | 32.6(14) | 26.3(14) | -6.9(11) | -4.2(11) | 6.0(11) |
| C_23_ | 26.3(13) | 27.0(13) | 20.4(13) | -1.9(10) | 1.7(10) | 2.0(10) |
| C_24_ | 27.7(13) | 29.6(13) | 18.8(12) | -5.2(10) | 2.8(10) | -2.0(11) |
| B_1_ | 24.0(14) | 20.7(13) | 14.6(13) | 0.5(10) | 6.2(10) | 3.5(11) |

| Table 4 Bond Lengths for SB-BDP. | | | | | | |
| --- | --- | --- | --- | --- | --- | --- |
| Atom | Atom | Length/Å |  | Atom | Atom | Length/Å |
| Br_1_ | C_1_ | 1.868(2) |  | C_4_ | C_5_ | 1.437(3) |
| Br_2_ | C_23_ | 1.875(3) |  | C_5_ | C_6_ | 1.349(3) |
| S_1_ | C_1_ | 1.722(3) |  | C_6_ | C_7_ | 1.434(3) |
| S_1_ | C_4_ | 1.735(2) |  | C_7_ | C_8_ | 1.407(3) |
| S_2_ | C_20_ | 1.738(2) |  | C_8_ | C_9_ | 1.381(3) |
| S_2_ | C_23_ | 1.720(3) |  | C_9_ | C_10_ | 1.438(3) |
| F_1_ | C_12_ | 1.343(3) |  | C_9_ | C_24_ | 1.506(3) |
| F_2_ | C_12_ | 1.341(3) |  | C_10_ | C_11_ | 1.408(3) |
| F_3_ | C_12_ | 1.342(3) |  | C_11_ | C_12_ | 1.513(3) |
| F_4_ | B_1_ | 1.390(3) |  | C_11_ | C_13_ | 1.404(3) |
| F_5_ | B_1_ | 1.395(3) |  | C_13_ | C_14_ | 1.447(3) |
| N_1_ | C_7_ | 1.364(3) |  | C_14_ | C_15_ | 1.499(3) |
| N_1_ | C_10_ | 1.389(3) |  | C_14_ | C_16_ | 1.370(4) |
| N_1_ | B_1_ | 1.546(3) |  | C_16_ | C_17_ | 1.416(3) |
| N_2_ | C_13_ | 1.395(3) |  | C_17_ | C_18_ | 1.440(3) |
| N_2_ | C_17_ | 1.366(3) |  | C_18_ | C_19_ | 1.349(3) |
| N_2_ | B_1_ | 1.545(3) |  | C_19_ | C_20_ | 1.439(3) |
| C_1_ | C_2_ | 1.355(4) |  | C_20_ | C_21_ | 1.374(3) |
| C_2_ | C_3_ | 1.415(3) |  | C_21_ | C_22_ | 1.405(4) |
| C_3_ | C_4_ | 1.376(3) |  | C_22_ | C_23_ | 1.359(4) |

| Table 5 Bond Angles for SB-BDP. | | | | | | | | |
| --- | --- | --- | --- | --- | --- | --- | --- | --- |
| Atom | Atom | Atom | Angle/˚ |  | Atom | Atom | Atom | Angle/˚ |
| C_1_ | S_1_ | C_4_ | 90.91(12) |  | F_2_ | C_12_ | F_1_ | 107.30(19) |
| C_23_ | S_2_ | C_20_ | 91.02(12) |  | F_2_ | C_12_ | F_3_ | 106.98(19) |
| C_7_ | N_1_ | C_10_ | 108.6(2) |  | F_2_ | C_12_ | C_11_ | 113.75(19) |
| C_7_ | N_1_ | B_1_ | 125.1(2) |  | F_3_ | C_12_ | F_1_ | 105.37(18) |
| C_10_ | N_1_ | B_1_ | 126.0(2) |  | F_3_ | C_12_ | C_11_ | 112.0(2) |
| C_13_ | N_2_ | B_1_ | 125.6(2) |  | N_2_ | C_13_ | C_11_ | 118.9(2) |
| C_17_ | N_2_ | C_13_ | 108.75(19) |  | N_2_ | C_13_ | C_14_ | 107.5(2) |
| C_17_ | N_2_ | B_1_ | 125.6(2) |  | C_11_ | C_13_ | C_14_ | 133.1(2) |
| S_1_ | C_1_ | Br_1_ | 120.28(14) |  | C_13_ | C_14_ | C_15_ | 130.8(2) |
| C_2_ | C_1_ | Br_1_ | 126.50(19) |  | C_16_ | C_14_ | C_13_ | 106.3(2) |
| C_2_ | C_1_ | S_1_ | 113.20(19) |  | C_16_ | C_14_ | C_15_ | 122.7(2) |
| C_1_ | C_2_ | C_3_ | 111.6(2) |  | C_14_ | C_16_ | C_17_ | 109.2(2) |
| C_4_ | C_3_ | C_2_ | 113.6(2) |  | N_2_ | C_17_ | C_16_ | 108.1(2) |
| C_3_ | C_4_ | S_1_ | 110.71(18) |  | N_2_ | C_17_ | C_18_ | 123.7(2) |
| C_3_ | C_4_ | C_5_ | 125.8(2) |  | C_16_ | C_17_ | C_18_ | 128.0(2) |
| C_5_ | C_4_ | S_1_ | 123.45(18) |  | C_19_ | C_18_ | C_17_ | 121.7(2) |
| C_6_ | C_5_ | C_4_ | 126.8(2) |  | C_18_ | C_19_ | C_20_ | 126.9(2) |
| C_5_ | C_6_ | C_7_ | 121.6(2) |  | C_19_ | C_20_ | S_2_ | 123.90(18) |
| N_1_ | C_7_ | C_6_ | 123.8(2) |  | C_21_ | C_20_ | S_2_ | 110.55(18) |
| N_1_ | C_7_ | C_8_ | 108.3(2) |  | C_21_ | C_20_ | C_19_ | 125.4(2) |
| C_8_ | C_7_ | C_6_ | 127.8(2) |  | C_20_ | C_21_ | C_22_ | 113.7(2) |
| C_9_ | C_8_ | C_7_ | 109.1(2) |  | C_23_ | C_22_ | C_21_ | 112.1(2) |
| C_8_ | C_9_ | C_10_ | 105.9(2) |  | S_2_ | C_23_ | Br_2_ | 121.14(14) |
| C_8_ | C_9_ | C_24_ | 122.2(2) |  | C_22_ | C_23_ | Br_2_ | 126.1(2) |
| C_10_ | C_9_ | C_24_ | 131.8(2) |  | C_22_ | C_23_ | S_2_ | 112.7(2) |
| N_1_ | C_10_ | C_9_ | 108.1(2) |  | F_4_ | B_1_ | F_5_ | 109.4(2) |
| N_1_ | C_10_ | C_11_ | 118.7(2) |  | F_4_ | B_1_ | N_1_ | 111.0(2) |
| C_11_ | C_10_ | C_9_ | 132.7(2) |  | F_4_ | B_1_ | N_2_ | 111.7(2) |
| C_10_ | C_11_ | C_12_ | 118.4(2) |  | F_5_ | B_1_ | N_1_ | 109.2(2) |
| C_13_ | C_11_ | C_10_ | 121.9(2) |  | F_5_ | B_1_ | N_2_ | 109.2(2) |
| C_13_ | C_11_ | C_12_ | 119.6(2) |  | N_2_ | B_1_ | N_1_ | 106.38(19) |
| F_1_ | C_12_ | C_11_ | 110.9(2) |  |  |  |  |  |

| Table 6 Torsion Angles for SB-BDP. | | | | | | | | | | |
| --- | --- | --- | --- | --- | --- | --- | --- | --- | --- | --- |
| A | B | C | D | Angle/˚ |  | A | B | C | D | Angle/˚ |
| Br_1_ | C_1_ | C_2_ | C_3_ | 177.64(18) |  | C_11_ | C_13_ | C_14_ | C_15_ | -4.8(4) |
| S_1_ | C_1_ | C_2_ | C_3_ | -0.6(3) |  | C_11_ | C_13_ | C_14_ | C_16_ | 171.5(3) |
| S_1_ | C_4_ | C_5_ | C_6_ | -2.9(4) |  | C_12_ | C_11_ | C_13_ | N_2_ | -168.3(2) |
| S_2_ | C_20_ | C_21_ | C_22_ | 0.8(3) |  | C_12_ | C_11_ | C_13_ | C_14_ | 21.4(4) |
| N_1_ | C_7_ | C_8_ | C_9_ | 3.1(3) |  | C_13_ | N_2_ | C_17_ | C_16_ | 1.7(3) |
| N_1_ | C_10_ | C_11_ | C_12_ | 166.1(2) |  | C_13_ | N_2_ | C_17_ | C_18_ | -175.0(2) |
| N_1_ | C_10_ | C_11_ | C_13_ | -13.4(3) |  | C_13_ | N_2_ | B_1_ | F_4_ | 107.7(3) |
| N_2_ | C_13_ | C_14_ | C_15_ | -175.9(2) |  | C_13_ | N_2_ | B_1_ | F_5_ | -131.2(2) |
| N_2_ | C_13_ | C_14_ | C_16_ | 0.4(3) |  | C_13_ | N_2_ | B_1_ | N_1_ | -13.5(3) |
| N_2_ | C_17_ | C_18_ | C_19_ | -177.0(2) |  | C_13_ | C_11_ | C_12_ | F_1_ | 149.3(2) |
| C_1_ | S_1_ | C_4_ | C_3_ | -1.47(19) |  | C_13_ | C_11_ | C_12_ | F_2_ | -89.6(3) |
| C_1_ | S_1_ | C_4_ | C_5_ | 176.2(2) |  | C_13_ | C_11_ | C_12_ | F_3_ | 31.9(3) |
| C_1_ | C_2_ | C_3_ | C_4_ | -0.5(3) |  | C_13_ | C_14_ | C_16_ | C_17_ | 0.6(3) |
| C_2_ | C_3_ | C_4_ | S_1_ | 1.4(3) |  | C_14_ | C_16_ | C_17_ | N_2_ | -1.5(3) |
| C_2_ | C_3_ | C_4_ | C_5_ | -176.2(2) |  | C_14_ | C_16_ | C_17_ | C_18_ | 175.1(2) |
| C_3_ | C_4_ | C_5_ | C_6_ | 174.4(2) |  | C_15_ | C_14_ | C_16_ | C_17_ | 177.3(2) |
| C_4_ | S_1_ | C_1_ | Br_1_ | -177.17(15) |  | C_16_ | C_17_ | C_18_ | C_19_ | 7.0(4) |
| C_4_ | S_1_ | C_1_ | C_2_ | 1.2(2) |  | C_17_ | N_2_ | C_13_ | C_11_ | -173.9(2) |
| C_4_ | C_5_ | C_6_ | C_7_ | 178.5(2) |  | C_17_ | N_2_ | C_13_ | C_14_ | -1.3(3) |
| C_5_ | C_6_ | C_7_ | N_1_ | 174.1(2) |  | C_17_ | N_2_ | B_1_ | F_4_ | -75.2(3) |
| C_5_ | C_6_ | C_7_ | C_8_ | -9.0(4) |  | C_17_ | N_2_ | B_1_ | F_5_ | 45.9(3) |
| C_6_ | C_7_ | C_8_ | C_9_ | -174.2(2) |  | C_17_ | N_2_ | B_1_ | N_1_ | 163.6(2) |
| C_7_ | N_1_ | C_10_ | C_9_ | 2.2(3) |  | C_17_ | C_18_ | C_19_ | C_20_ | -175.3(2) |
| C_7_ | N_1_ | C_10_ | C_11_ | 174.7(2) |  | C_18_ | C_19_ | C_20_ | S_2_ | 0.0(4) |
| C_7_ | N_1_ | B_1_ | F_4_ | 76.6(3) |  | C_18_ | C_19_ | C_20_ | C_21_ | 174.6(3) |
| C_7_ | N_1_ | B_1_ | F_5_ | -44.0(3) |  | C_19_ | C_20_ | C_21_ | C_22_ | -174.4(2) |
| C_7_ | N_1_ | B_1_ | N_2_ | -161.7(2) |  | C_20_ | S_2_ | C_23_ | Br_2_ | 178.91(17) |
| C_7_ | C_8_ | C_9_ | C_10_ | -1.7(3) |  | C_20_ | S_2_ | C_23_ | C_22_ | 1.4(2) |
| C_7_ | C_8_ | C_9_ | C_24_ | -178.2(2) |  | C_20_ | C_21_ | C_22_ | C_23_ | 0.2(4) |
| C_8_ | C_9_ | C_10_ | N_1_ | -0.3(3) |  | C_21_ | C_22_ | C_23_ | Br_2_ | -178.6(2) |
| C_8_ | C_9_ | C_10_ | C_11_ | -171.4(2) |  | C_21_ | C_22_ | C_23_ | S_2_ | -1.1(3) |
| C_9_ | C_10_ | C_11_ | C_12_ | -23.5(4) |  | C_23_ | S_2_ | C_20_ | C_19_ | 174.1(2) |
| C_9_ | C_10_ | C_11_ | C_13_ | 156.9(2) |  | C_23_ | S_2_ | C_20_ | C_21_ | -1.2(2) |
| C_10_ | N_1_ | C_7_ | C_6_ | 174.2(2) |  | C_24_ | C_9_ | C_10_ | N_1_ | 175.8(2) |
| C_10_ | N_1_ | C_7_ | C_8_ | -3.3(3) |  | C_24_ | C_9_ | C_10_ | C_11_ | 4.7(4) |
| C_10_ | N_1_ | B_1_ | F_4_ | -110.5(2) |  | B_1_ | N_1_ | C_7_ | C_6_ | -11.9(4) |
| C_10_ | N_1_ | B_1_ | F_5_ | 128.9(2) |  | B_1_ | N_1_ | C_7_ | C_8_ | 170.7(2) |
| C_10_ | N_1_ | B_1_ | N_2_ | 11.2(3) |  | B_1_ | N_1_ | C_10_ | C_9_ | -171.7(2) |
| C_10_ | C_11_ | C_12_ | F_1_ | -30.3(3) |  | B_1_ | N_1_ | C_10_ | C_11_ | 0.9(3) |
| C_10_ | C_11_ | C_12_ | F_2_ | 90.8(3) |  | B_1_ | N_2_ | C_13_ | C_11_ | 3.7(3) |
| C_10_ | C_11_ | C_12_ | F_3_ | -147.7(2) |  | B_1_ | N_2_ | C_13_ | C_14_ | 176.2(2) |
| C_10_ | C_11_ | C_13_ | N_2_ | 11.3(3) |  | B_1_ | N_2_ | C_17_ | C_16_ | -175.8(2) |
| C_10_ | C_11_ | C_13_ | C_14_ | -159.0(2) |  | B_1_ | N_2_ | C_17_ | C_18_ | 7.4(4) |

| Table 7 Hydrogen Atom Coordinates (Å×10^4^) and Isotropic Displacement Parameters (Å^2^×10^3^) for SB-BDP. | | | | |
| --- | --- | --- | --- | --- |
| Atom | *x* | *y* | *z* | U(eq) |
| H_2_ | -526.44 | 6447.53 | 6374.71 | 25 |
| H_3_ | 345.3 | 5765.8 | 5127.27 | 24 |
| H_5_ | 2407.8 | 4861.02 | 4893.31 | 23 |
| H_6_ | 4530.59 | 4476.98 | 6510.25 | 23 |
| H_8_ | 3195.24 | 4138.36 | 3917.09 | 25 |
| H_15A_ | 11744.69 | 1935.85 | 5079.9 | 38 |
| H_15B_ | 13308.04 | 2415.2 | 5047.08 | 38 |
| H_15C_ | 11617.95 | 2436.65 | 4270.85 | 38 |
| H_16_ | 13151.34 | 2978.76 | 6575.27 | 25 |
| H_18_ | 10012.45 | 3932.19 | 7882.73 | 23 |
| H_19_ | 13582.87 | 3602.61 | 7840.25 | 23 |
| H_21_ | 15731.69 | 3894 | 9304.46 | 30 |
| H_22_ | 15752.4 | 4359.88 | 10878.96 | 35 |
| H_24A_ | 5869 | 3143.35 | 2697.43 | 38 |
| H_24B_ | 4056.88 | 3517.88 | 2555.82 | 38 |
| H_24C_ | 4036.88 | 2852.44 | 2956.52 | 38 |

Experimental

Single crystals of C_24_H_16_BBr_2_F_5_N_2_S_2_ [SB-BDP] were []. A suitable crystal was selected and [] on a ROD, Synergy Custom system, HyPix-Arc 150 diffractometer. The crystal was kept at 100.03(10) K during data collection. Using Olex2 [1], the structure was solved with the SHELXS [2] structure solution program using Direct Methods and refined with the SHELXL [3] refinement package using Least Squares minimisation.

1. Dolomanov, O.V., Bourhis, L.J., Gildea, R.J, Howard, J.A.K. & Puschmann, H. (2009), J. Appl. Cryst. 42, 339-341.
2. Sheldrick, G.M. (2008). Acta Cryst. A64, 112-122.
3. Sheldrick, G.M. (2015). Acta Cryst. C71, 3-8.

Refinement model description

Number of restraints - 0, number of constraints - unknown.

Details:

1. Fixed Uiso
 At 1.2 times of:
 All C(H) groups
 At 1.5 times of:
 All C(H,H,H) groups
2.a Aromatic/amide H refined with riding coordinates:
 C2(H2), C3(H3), C5(H5), C6(H6), C8(H8), C16(H16), C18(H18), C19(H19),
 C21(H21), C22(H22)
2.b Idealised Me refined as rotating group:
 C15(H15A,H15B,H15C), C24(H24A,H24B,H24C)

**SH-BDP**

| **Table 1 Crystal data and structure refinement for SH-BDP.** | |
| --- | --- |
| Identification code | SH-BDP |
| Empirical formula | C_24_H_18_BF_5_N_2_S_2_ |
| Formula weight | 504.33 |
| Temperature/K | 100.00(10) |
| Crystal system | monoclinic |
| Space group | P2_1_/c |
| a/Å | 11.6200(6) |
| b/Å | 24.5194(7) |
| c/Å | 8.1610(3) |
| α/° | 90 |
| β/° | 102.248(4) |
| γ/° | 90 |
| Volume/Å^3^ | 2272.27(16) |
| Z | 4 |
| ρ_calc_g/cm^3^ | 1.474 |
| μ/mm^‑1^ | 2.638 |
| F(000) | 1032.0 |
| Crystal size/mm^3^ | 0.15 × 0.13 × 0.12 |
| Radiation | Cu Kα (λ = 1.54184) |
| 2Θ range for data collection/° | 7.21 to 149.404 |
| Index ranges | -14 ≤ h ≤ 14, -19 ≤ k ≤ 30, -10 ≤ l ≤ 10 |
| Reflections collected | 17183 |
| Independent reflections | 4432 [R_int_ = 0.0308, R_sigma_ = 0.0178] |
| Data/restraints/parameters | 4432/0/309 |
| Goodness-of-fit on F^2^ | 0.990 |
| Final R indexes [I>=2σ (I)] | R_1_ = 0.0600, wR_2_ = 0.1433 |
| Final R indexes [all data] | R_1_ = 0.0652, wR_2_ = 0.1457 |
| Largest diff. peak/hole / e Å^-3^ | 0.43/-0.52 |

Crystal structure determination of [SH-BDP]

Crystal Data for C_24_H_18_BF_5_N_2_S_2_ (*M* =504.33 g/mol): monoclinic, space group P2_1_/c (no. 14), *a* = 11.6200(6) Å, *b* = 24.5194(7) Å, *c* = 8.1610(3) Å, *β* = 102.248(4)°, *V* = 2272.27(16) Å^3^, *Z* = 4, *T* = 100.00(10) K, μ(Cu Kα) = 2.638 mm^-1^, *Dcalc* = 1.474 g/cm^3^, 17183 reflections measured (7.21° ≤ 2Θ ≤ 149.404°), 4432 unique (*R*_int_ = 0.0308, R_sigma_ = 0.0178) which were used in all calculations. The final *R*_1_ was 0.0600 (I > 2σ(I)) and *wR*_2_ was 0.1457 (all data).

Refinement model description

| Table 2 Fractional Atomic Coordinates (×10^4^) and Equivalent Isotropic Displacement Parameters (Å^2^×10^3^) for SH-BDP. U_eq_ is defined as 1/3 of the trace of the orthogonalised U_IJ_ tensor. | | | | |
| --- | --- | --- | --- | --- |
| Atom | *x* | *y* | *z* | U(eq) |
| S_1_ | 2437.3(8) | 5132.9(4) | 8885.7(11) | 32.0(2) |
| S_2_ | 2217.3(9) | 2239.6(4) | 7072.3(14) | 41.3(3) |
| F_1_ | 9748.9(17) | 4262.0(8) | 7217(2) | 27.6(4) |
| F_2_ | 9506.1(18) | 3929.7(8) | 4722(2) | 30.0(5) |
| F_3_ | 9696.1(17) | 3399.6(8) | 6877(3) | 30.2(5) |
| F_4_ | 4355.0(16) | 3854.8(7) | 5684(2) | 26.0(4) |
| F_5_ | 5304.1(17) | 3698.1(7) | 8366(2) | 24.6(4) |
| N_2_ | 6145(2) | 3333.3(10) | 6116(3) | 19.1(5) |
| N_4_ | 6190(2) | 4324.3(10) | 6699(3) | 18.8(5) |
| C_1_ | 7317(3) | 4350.9(12) | 6374(4) | 20.0(6) |
| C_2_ | 7627(3) | 4922.1(13) | 6322(4) | 21.8(7) |
| C_3_ | 6699(3) | 5210.2(13) | 6664(4) | 21.6(6) |
| C_4_ | 5820(3) | 4840.1(12) | 6935(4) | 20.2(6) |
| C_5_ | 4731(3) | 4955.8(13) | 7427(4) | 22.5(7) |
| C_6_ | 4356(3) | 5470.3(13) | 7605(4) | 23.5(7) |
| C_7_ | 3311(3) | 5611.0(14) | 8192(4) | 25.6(7) |
| C_8_ | 2883(3) | 6142.0(14) | 8385(4) | 26.3(7) |
| C_9_ | 1884(4) | 6133.7(17) | 9087(5) | 40.5(10) |
| C_10_ | 1536(4) | 5624.6(18) | 9418(5) | 38.5(9) |
| C_11_ | 9190(3) | 3865.1(13) | 6203(4) | 23.7(7) |
| C_12_ | 7884(3) | 3865.6(13) | 6094(4) | 20.4(6) |
| C_13_ | 7275(3) | 3365.9(13) | 5814(4) | 20.1(6) |
| C_14_ | 7533(3) | 2851.6(13) | 5116(4) | 21.5(7) |
| C_15_ | 6557(3) | 2528.1(13) | 5048(4) | 21.3(7) |
| C_16_ | 5706(3) | 2821.2(13) | 5702(4) | 21.6(7) |
| C_17_ | 4599(3) | 2640.1(13) | 6015(4) | 22.1(7) |
| C_18_ | 4190(3) | 2130.0(13) | 5649(4) | 24.0(7) |
| C_19_ | 3116(3) | 1904.9(14) | 5968(4) | 24.4(7) |
| C_20_ | 2650(3) | 1388.8(14) | 5442(5) | 30.6(8) |
| C_21_ | 1602(4) | 1285.5(17) | 5997(5) | 40.9(10) |
| C_22_ | 1272(3) | 1700.6(18) | 6878(5) | 41.1(10) |
| C_23_ | 8578(3) | 2676.5(14) | 4432(4) | 26.7(7) |
| C_24_ | 8660(3) | 5188.6(14) | 5807(5) | 27.5(7) |
| B_1_ | 5452(3) | 3800.5(14) | 6743(5) | 19.6(7) |

| Table 3 Anisotropic Displacement Parameters (Å^2^×10^3^) for SH-BDP. The Anisotropic displacement factor exponent takes the form: -2π^2^[h^2^a*^2^U_11_+2hka*b*U_12_+…]. | | | | | | |
| --- | --- | --- | --- | --- | --- | --- |
| Atom | U_11_ | U_22_ | U_33_ | U_23_ | U_13_ | U_12_ |
| S_1_ | 32.0(5) | 37.0(5) | 28.8(5) | 2.1(4) | 10.5(4) | 3.9(4) |
| S_2_ | 31.3(5) | 47.8(6) | 49.3(6) | -12.8(5) | 18.9(4) | -8.8(4) |
| F_1_ | 22.3(10) | 31.4(10) | 28.4(10) | -6.7(8) | 4.2(8) | -5.3(8) |
| F_2_ | 30.4(11) | 38.7(11) | 24.8(10) | -2.8(8) | 14.6(8) | -6.7(9) |
| F_3_ | 24.5(10) | 27.4(10) | 37.4(11) | 2.4(9) | 3.8(9) | 2.2(8) |
| F_4_ | 22.0(10) | 20.9(9) | 32.8(11) | 1.2(8) | 0.8(8) | 0.2(7) |
| F_5_ | 33.1(11) | 21.5(9) | 22.3(9) | 1.8(7) | 13.0(8) | -1.5(8) |
| N_2_ | 21.8(14) | 16.4(12) | 19.4(13) | -0.8(10) | 5.3(11) | -0.6(10) |
| N_4_ | 25.3(14) | 14.9(12) | 16.4(12) | 0.1(10) | 4.6(10) | -1.8(10) |
| C_1_ | 25.0(17) | 19.3(15) | 15.3(14) | 0.8(11) | 3.2(12) | -2.6(12) |
| C_2_ | 27.3(17) | 21.2(16) | 16.3(14) | 1.4(12) | 3.6(13) | -4.3(13) |
| C_3_ | 28.3(17) | 18.7(15) | 17.4(15) | -0.3(12) | 3.9(13) | -2.7(12) |
| C_4_ | 26.8(17) | 18.5(15) | 13.8(14) | 0.0(11) | 1.2(12) | 0.0(12) |
| C_5_ | 28.1(17) | 19.8(15) | 18.4(15) | 0.0(12) | 2.6(13) | -2.4(13) |
| C_6_ | 28.3(18) | 22.9(16) | 18.3(15) | -1.2(12) | 3.0(13) | -0.5(13) |
| C_7_ | 31.3(19) | 25.7(17) | 18.0(16) | -1.4(13) | 1.0(13) | 3.7(14) |
| C_8_ | 31.3(19) | 24.5(17) | 18.6(15) | -3.6(13) | -4.9(14) | 4.6(14) |
| C_9_ | 45(2) | 44(2) | 28.0(19) | -9.8(17) | -1.4(17) | 20.7(19) |
| C_10_ | 31(2) | 60(3) | 23.3(18) | -4.8(17) | 4.1(15) | 12.3(18) |
| C_11_ | 30.7(18) | 21.4(16) | 19.5(15) | -1.2(12) | 6.7(13) | -2.0(13) |
| C_12_ | 25.5(17) | 21.4(16) | 15.4(14) | 1.5(12) | 6.6(12) | -2.1(12) |
| C_13_ | 25.4(17) | 19.4(15) | 15.8(14) | 1.5(12) | 5.1(12) | -0.8(12) |
| C_14_ | 25.1(17) | 21.4(16) | 18.2(15) | 1.7(12) | 5.0(13) | 1.3(12) |
| C_15_ | 22.7(16) | 20.5(15) | 21.2(16) | -1.2(12) | 6.0(13) | 0.0(12) |
| C_16_ | 29.4(18) | 17.2(15) | 18.2(15) | 0.2(12) | 5.1(13) | -1.5(13) |
| C_17_ | 23.8(17) | 23.6(16) | 20.0(16) | -1.9(12) | 6.9(13) | 0.0(13) |
| C_18_ | 28.3(18) | 22.0(16) | 23.2(16) | -1.8(13) | 8.4(14) | -0.9(13) |
| C_19_ | 24.9(17) | 25.6(17) | 21.5(16) | 0.6(13) | 2.5(13) | -2.8(13) |
| C_20_ | 29.7(19) | 19.9(16) | 43(2) | 5.0(15) | 8.4(16) | -4.9(14) |
| C_21_ | 37(2) | 33(2) | 47(2) | 10.3(18) | -2.5(18) | -12.4(17) |
| C_22_ | 20.9(19) | 58(3) | 46(2) | 10(2) | 10.1(17) | -7.5(17) |
| C_23_ | 29.7(19) | 23.8(17) | 29.3(18) | -2.5(14) | 12.4(15) | 0.5(13) |
| C_24_ | 29.3(18) | 25.2(17) | 30.1(18) | 0.0(14) | 10.5(15) | -6.6(14) |
| B_1_ | 21.4(18) | 16.9(16) | 22.0(17) | 0.5(13) | 8.0(14) | 0.7(13) |

| Table 4 Bond Lengths for SH-BDP. | | | | | | |
| --- | --- | --- | --- | --- | --- | --- |
| Atom | Atom | Length/Å |  | Atom | Atom | Length/Å |
| S_1_ | C_7_ | 1.723(4) |  | C_3_ | C_4_ | 1.419(4) |
| S_1_ | C_10_ | 1.712(4) |  | C_4_ | C_5_ | 1.434(5) |
| S_2_ | C_19_ | 1.725(4) |  | C_5_ | C_6_ | 1.352(5) |
| S_2_ | C_22_ | 1.705(4) |  | C_6_ | C_7_ | 1.439(5) |
| F_1_ | C_11_ | 1.351(4) |  | C_7_ | C_8_ | 1.414(5) |
| F_2_ | C_11_ | 1.346(4) |  | C_8_ | C_9_ | 1.399(6) |
| F_3_ | C_11_ | 1.347(4) |  | C_9_ | C_10_ | 1.357(6) |
| F_4_ | B_1_ | 1.387(4) |  | C_11_ | C_12_ | 1.501(5) |
| F_5_ | B_1_ | 1.394(4) |  | C_12_ | C_13_ | 1.409(4) |
| N_2_ | C_13_ | 1.388(4) |  | C_13_ | C_14_ | 1.442(4) |
| N_2_ | C_16_ | 1.371(4) |  | C_14_ | C_15_ | 1.376(5) |
| N_2_ | B_1_ | 1.548(4) |  | C_14_ | C_23_ | 1.503(5) |
| N_4_ | C_1_ | 1.391(4) |  | C_15_ | C_16_ | 1.415(4) |
| N_4_ | C_4_ | 1.363(4) |  | C_16_ | C_17_ | 1.434(5) |
| N_4_ | B_1_ | 1.549(4) |  | C_17_ | C_18_ | 1.349(5) |
| C_1_ | C_2_ | 1.449(4) |  | C_18_ | C_19_ | 1.438(5) |
| C_1_ | C_12_ | 1.402(4) |  | C_19_ | C_20_ | 1.407(5) |
| C_2_ | C_3_ | 1.366(5) |  | C_20_ | C_21_ | 1.409(6) |
| C_2_ | C_24_ | 1.503(4) |  | C_21_ | C_22_ | 1.348(6) |

| Table 5 Bond Angles for SH-BDP. | | | | | | | | |
| --- | --- | --- | --- | --- | --- | --- | --- | --- |
| Atom | Atom | Atom | Angle/˚ |  | Atom | Atom | Atom | Angle/˚ |
| C_10_ | S_1_ | C_7_ | 92.2(2) |  | F_3_ | C_11_ | F_1_ | 104.4(3) |
| C_22_ | S_2_ | C_19_ | 91.83(19) |  | F_3_ | C_11_ | C_12_ | 111.9(3) |
| C_13_ | N_2_ | B_1_ | 126.4(3) |  | C_1_ | C_12_ | C_11_ | 119.7(3) |
| C_16_ | N_2_ | C_13_ | 109.0(3) |  | C_1_ | C_12_ | C_13_ | 121.8(3) |
| C_16_ | N_2_ | B_1_ | 124.5(3) |  | C_13_ | C_12_ | C_11_ | 118.4(3) |
| C_1_ | N_4_ | B_1_ | 126.2(3) |  | N_2_ | C_13_ | C_12_ | 118.9(3) |
| C_4_ | N_4_ | C_1_ | 108.8(3) |  | N_2_ | C_13_ | C_14_ | 107.7(3) |
| C_4_ | N_4_ | B_1_ | 125.0(3) |  | C_12_ | C_13_ | C_14_ | 133.2(3) |
| N_4_ | C_1_ | C_2_ | 107.5(3) |  | C_13_ | C_14_ | C_23_ | 130.5(3) |
| N_4_ | C_1_ | C_12_ | 119.0(3) |  | C_15_ | C_14_ | C_13_ | 106.3(3) |
| C_12_ | C_1_ | C_2_ | 133.4(3) |  | C_15_ | C_14_ | C_23_ | 123.0(3) |
| C_1_ | C_2_ | C_24_ | 130.3(3) |  | C_14_ | C_15_ | C_16_ | 109.2(3) |
| C_3_ | C_2_ | C_1_ | 106.4(3) |  | N_2_ | C_16_ | C_15_ | 107.7(3) |
| C_3_ | C_2_ | C_24_ | 123.0(3) |  | N_2_ | C_16_ | C_17_ | 122.8(3) |
| C_2_ | C_3_ | C_4_ | 109.1(3) |  | C_15_ | C_16_ | C_17_ | 129.4(3) |
| N_4_ | C_4_ | C_3_ | 108.2(3) |  | C_18_ | C_17_ | C_16_ | 122.4(3) |
| N_4_ | C_4_ | C_5_ | 123.2(3) |  | C_17_ | C_18_ | C_19_ | 126.5(3) |
| C_3_ | C_4_ | C_5_ | 128.6(3) |  | C_18_ | C_19_ | S_2_ | 123.8(3) |
| C_6_ | C_5_ | C_4_ | 122.6(3) |  | C_20_ | C_19_ | S_2_ | 110.6(3) |
| C_5_ | C_6_ | C_7_ | 125.0(3) |  | C_20_ | C_19_ | C_18_ | 125.6(3) |
| C_6_ | C_7_ | S_1_ | 122.9(3) |  | C_19_ | C_20_ | C_21_ | 111.5(4) |
| C_8_ | C_7_ | S_1_ | 110.1(3) |  | C_22_ | C_21_ | C_20_ | 113.5(4) |
| C_8_ | C_7_ | C_6_ | 126.8(3) |  | C_21_ | C_22_ | S_2_ | 112.6(3) |
| C_9_ | C_8_ | C_7_ | 112.0(3) |  | F_4_ | B_1_ | F_5_ | 109.1(3) |
| C_10_ | C_9_ | C_8_ | 113.7(3) |  | F_4_ | B_1_ | N_2_ | 109.8(3) |
| C_9_ | C_10_ | S_1_ | 111.9(3) |  | F_4_ | B_1_ | N_4_ | 110.3(3) |
| F_1_ | C_11_ | C_12_ | 112.6(3) |  | F_5_ | B_1_ | N_2_ | 111.0(3) |
| F_2_ | C_11_ | F_1_ | 106.1(3) |  | F_5_ | B_1_ | N_4_ | 110.6(3) |
| F_2_ | C_11_ | F_3_ | 106.7(3) |  | N_2_ | B_1_ | N_4_ | 106.0(3) |
| F_2_ | C_11_ | C_12_ | 114.3(3) |  |  |  |  |  |

| Table 6 Torsion Angles for SH-BDP. | | | | | | | | | | |
| --- | --- | --- | --- | --- | --- | --- | --- | --- | --- | --- |
| A | B | C | D | Angle/˚ |  | A | B | C | D | Angle/˚ |
| S_1_ | C_7_ | C_8_ | C_9_ | 0.4(4) |  | C_10_ | S_1_ | C_7_ | C_6_ | 176.5(3) |
| S_2_ | C_19_ | C_20_ | C_21_ | -1.4(4) |  | C_10_ | S_1_ | C_7_ | C_8_ | -0.1(3) |
| F_1_ | C_11_ | C_12_ | C_1_ | 26.2(4) |  | C_11_ | C_12_ | C_13_ | N_2_ | 164.3(3) |
| F_1_ | C_11_ | C_12_ | C_13_ | -149.9(3) |  | C_11_ | C_12_ | C_13_ | C_14_ | -21.6(5) |
| F_2_ | C_11_ | C_12_ | C_1_ | -95.1(3) |  | C_12_ | C_1_ | C_2_ | C_3_ | 178.3(3) |
| F_2_ | C_11_ | C_12_ | C_13_ | 88.9(4) |  | C_12_ | C_1_ | C_2_ | C_24_ | 5.3(6) |
| F_3_ | C_11_ | C_12_ | C_1_ | 143.5(3) |  | C_12_ | C_13_ | C_14_ | C_15_ | -175.4(3) |
| F_3_ | C_11_ | C_12_ | C_13_ | -32.6(4) |  | C_12_ | C_13_ | C_14_ | C_23_ | -0.1(6) |
| N_2_ | C_13_ | C_14_ | C_15_ | -0.8(3) |  | C_13_ | N_2_ | C_16_ | C_15_ | -2.7(3) |
| N_2_ | C_13_ | C_14_ | C_23_ | 174.5(3) |  | C_13_ | N_2_ | C_16_ | C_17_ | 174.0(3) |
| N_2_ | C_16_ | C_17_ | C_18_ | -178.0(3) |  | C_13_ | N_2_ | B_1_ | F_4_ | 127.6(3) |
| N_4_ | C_1_ | C_2_ | C_3_ | 1.7(3) |  | C_13_ | N_2_ | B_1_ | F_5_ | -111.7(3) |
| N_4_ | C_1_ | C_2_ | C_24_ | -171.2(3) |  | C_13_ | N_2_ | B_1_ | N_4_ | 8.4(4) |
| N_4_ | C_1_ | C_12_ | C_11_ | -163.5(3) |  | C_13_ | C_14_ | C_15_ | C_16_ | -0.8(4) |
| N_4_ | C_1_ | C_12_ | C_13_ | 12.4(5) |  | C_14_ | C_15_ | C_16_ | N_2_ | 2.2(4) |
| N_4_ | C_4_ | C_5_ | C_6_ | -177.8(3) |  | C_14_ | C_15_ | C_16_ | C_17_ | -174.3(3) |
| C_1_ | N_4_ | C_4_ | C_3_ | 3.2(3) |  | C_15_ | C_16_ | C_17_ | C_18_ | -2.0(5) |
| C_1_ | N_4_ | C_4_ | C_5_ | -174.9(3) |  | C_16_ | N_2_ | C_13_ | C_12_ | 177.7(3) |
| C_1_ | N_4_ | B_1_ | F_4_ | -126.4(3) |  | C_16_ | N_2_ | C_13_ | C_14_ | 2.2(3) |
| C_1_ | N_4_ | B_1_ | F_5_ | 112.8(3) |  | C_16_ | N_2_ | B_1_ | F_4_ | -49.5(4) |
| C_1_ | N_4_ | B_1_ | N_2_ | -7.6(4) |  | C_16_ | N_2_ | B_1_ | F_5_ | 71.2(4) |
| C_1_ | C_2_ | C_3_ | C_4_ | 0.2(4) |  | C_16_ | N_2_ | B_1_ | N_4_ | -168.7(3) |
| C_1_ | C_12_ | C_13_ | N_2_ | -11.6(5) |  | C_16_ | C_17_ | C_18_ | C_19_ | 177.7(3) |
| C_1_ | C_12_ | C_13_ | C_14_ | 162.4(3) |  | C_17_ | C_18_ | C_19_ | S_2_ | -6.3(5) |
| C_2_ | C_1_ | C_12_ | C_11_ | 20.2(5) |  | C_17_ | C_18_ | C_19_ | C_20_ | 173.9(4) |
| C_2_ | C_1_ | C_12_ | C_13_ | -163.9(3) |  | C_18_ | C_19_ | C_20_ | C_21_ | 178.4(3) |
| C_2_ | C_3_ | C_4_ | N_4_ | -2.2(4) |  | C_19_ | S_2_ | C_22_ | C_21_ | -0.9(3) |
| C_2_ | C_3_ | C_4_ | C_5_ | 175.9(3) |  | C_19_ | C_20_ | C_21_ | C_22_ | 0.8(5) |
| C_3_ | C_4_ | C_5_ | C_6_ | 4.4(5) |  | C_20_ | C_21_ | C_22_ | S_2_ | 0.2(5) |
| C_4_ | N_4_ | C_1_ | C_2_ | -3.1(3) |  | C_22_ | S_2_ | C_19_ | C_18_ | -178.6(3) |
| C_4_ | N_4_ | C_1_ | C_12_ | 179.8(3) |  | C_22_ | S_2_ | C_19_ | C_20_ | 1.3(3) |
| C_4_ | N_4_ | B_1_ | F_4_ | 51.8(4) |  | C_23_ | C_14_ | C_15_ | C_16_ | -176.5(3) |
| C_4_ | N_4_ | B_1_ | F_5_ | -69.0(4) |  | C_24_ | C_2_ | C_3_ | C_4_ | 173.8(3) |
| C_4_ | N_4_ | B_1_ | N_2_ | 170.6(3) |  | B_1_ | N_2_ | C_13_ | C_12_ | 0.2(5) |
| C_4_ | C_5_ | C_6_ | C_7_ | -176.0(3) |  | B_1_ | N_2_ | C_13_ | C_14_ | -175.3(3) |
| C_5_ | C_6_ | C_7_ | S_1_ | 4.4(5) |  | B_1_ | N_2_ | C_16_ | C_15_ | 174.8(3) |
| C_5_ | C_6_ | C_7_ | C_8_ | -179.5(3) |  | B_1_ | N_2_ | C_16_ | C_17_ | -8.4(5) |
| C_6_ | C_7_ | C_8_ | C_9_ | -176.2(3) |  | B_1_ | N_4_ | C_1_ | C_2_ | 175.4(3) |
| C_7_ | S_1_ | C_10_ | C_9_ | -0.1(3) |  | B_1_ | N_4_ | C_1_ | C_12_ | -1.8(5) |
| C_7_ | C_8_ | C_9_ | C_10_ | -0.5(5) |  | B_1_ | N_4_ | C_4_ | C_3_ | -175.2(3) |
| C_8_ | C_9_ | C_10_ | S_1_ | 0.4(4) |  | B_1_ | N_4_ | C_4_ | C_5_ | 6.6(5) |

| Table 7 Hydrogen Atom Coordinates (Å×10^4^) and Isotropic Displacement Parameters (Å^2^×10^3^) for SH-BDP. | | | | |
| --- | --- | --- | --- | --- |
| Atom | *x* | *y* | *z* | U(eq) |
| H_3_ | 6651.1 | 5596.39 | 6713.05 | 26 |
| H_5_ | 4255.5 | 4659.59 | 7635.71 | 27 |
| H_6_ | 4817.72 | 5760.92 | 7319.95 | 28 |
| H_8_ | 3231.81 | 6465.8 | 8074.76 | 32 |
| H_9_ | 1485.73 | 6454.7 | 9310.66 | 49 |
| H_10_ | 872.38 | 5549.82 | 9888.98 | 46 |
| H_15_ | 6467.89 | 2165.65 | 4628.21 | 26 |
| H_17_ | 4137.62 | 2888.24 | 6497.53 | 27 |
| H_18_ | 4657.31 | 1896.26 | 5127.82 | 29 |
| H_20_ | 2998.4 | 1141.12 | 4794.87 | 37 |
| H_21_ | 1169.7 | 955.25 | 5771.58 | 49 |
| H_22_ | 586.95 | 1693.47 | 7339.22 | 49 |
| H_23A_ | 8827.63 | 2977.69 | 3798.42 | 40 |
| H_23B_ | 8362.62 | 2361.66 | 3690.67 | 40 |
| H_23C_ | 9226.25 | 2575.88 | 5361.16 | 40 |
| H_24A_ | 9330.99 | 5197.32 | 6764.93 | 41 |
| H_24B_ | 8450.12 | 5561.89 | 5429.62 | 41 |
| H_24C_ | 8873.57 | 4980.33 | 4890.32 | 41 |

Experimental

Single crystals of C_24_H_18_BF_5_N_2_S_2_ [SH-BDP] were []. A suitable crystal was selected and [] on a ROD, Synergy Custom system, HyPix diffractometer. The crystal was kept at 100.00(10) K during data collection. Using Olex2 [1], the structure was solved with the SHELXS [2] structure solution program using Direct Methods and refined with the SHELXL [3] refinement package using Least Squares minimisation.

1. Dolomanov, O.V., Bourhis, L.J., Gildea, R.J, Howard, J.A.K. & Puschmann, H. (2009), J. Appl. Cryst. 42, 339-341.
2. Sheldrick, G.M. (2008). Acta Cryst. A64, 112-122.
3. Sheldrick, G.M. (2015). Acta Cryst. C71, 3-8.

Number of restraints - 0, number of constraints - unknown.

Details:

1. Fixed Uiso
 At 1.2 times of:
 All C(H) groups
 At 1.5 times of:
 All C(H,H,H) groups
2.a Aromatic/amide H refined with riding coordinates:
 C3(H3), C5(H5), C6(H6), C8(H8), C9(H9), C10(H10), C15(H15), C17(H17),
 C18(H18), C20(H20), C21(H21), C22(H22)
2.b Idealised Me refined as rotating group:
 C23(H23A,H23B,H23C), C24(H24A,H24B,H24C)
